# Targeting cancer-specific metabolic pathways for developing novel cancer therapeutics

**DOI:** 10.3389/fimmu.2022.955476

**Published:** 2022-12-22

**Authors:** Soumik Pal, Amit Sharma, Sam Padalumavunkal Mathew, Bithiah Grace Jaganathan

**Affiliations:** ^1^ Stem Cells and Cancer Biology Research Group, Department of Biosciences and Bioengineering, Indian Institute of Technology Guwahati, Guwahati, Assam, India; ^2^ Jyoti and Bhupat Mehta School of Health Sciences and Technology, Indian Institute of Technology Guwahati, Guwahati, Assam, India

**Keywords:** cancer, cancer metabolism, metabolic reprogramming, cancer microenvironment, targeted therapy

## Abstract

Cancer is a heterogeneous disease characterized by various genetic and phenotypic aberrations. Cancer cells undergo genetic modifications that promote their proliferation, survival, and dissemination as the disease progresses. The unabated proliferation of cancer cells incurs an enormous energy demand that is supplied by metabolic reprogramming. Cancer cells undergo metabolic alterations to provide for increased energy and metabolite requirement; these alterations also help drive the tumor progression. Dysregulation in glucose uptake and increased lactate production *via* “aerobic glycolysis” were described more than 100 years ago, and since then, the metabolic signature of various cancers has been extensively studied. However, the extensive research in this field has failed to translate into significant therapeutic intervention, except for treating childhood-ALL with amino acid metabolism inhibitor L-asparaginase. Despite the growing understanding of novel metabolic alterations in tumors, the therapeutic targeting of these tumor-specific dysregulations has largely been ineffective in clinical trials. This chapter discusses the major pathways involved in the metabolism of glucose, amino acids, and lipids and highlights the inter-twined nature of metabolic aberrations that promote tumorigenesis in different types of cancer. Finally, we summarise the therapeutic interventions which can be used as a combinational therapy to target metabolic dysregulations that are unique or common in blood, breast, colorectal, lung, and prostate cancer.

## Introduction

**Figure 1:**
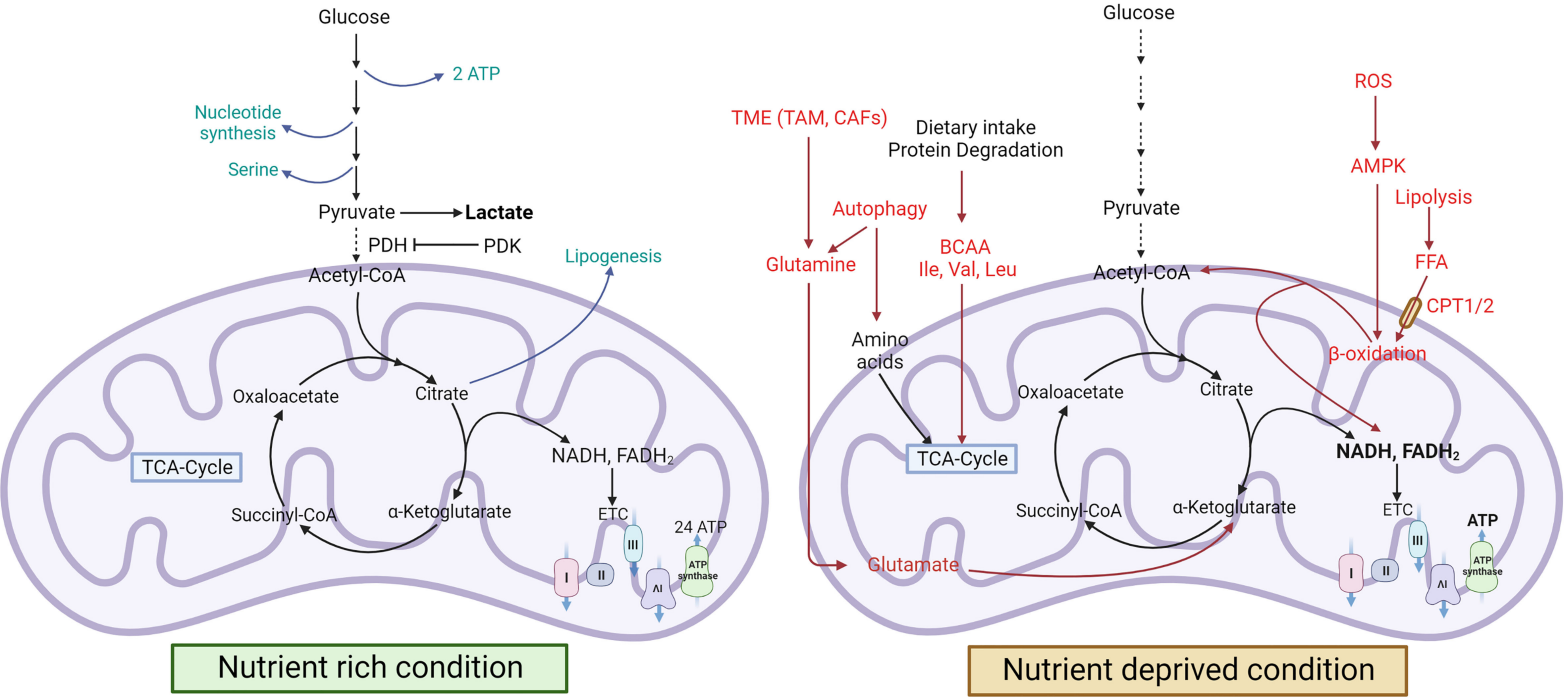
Cancer metabolism during nutrient-rich and stressed conditions. In nutrient-rich conditions, cancer cells use glucose to produce lactate, serine, and nucleotides, but in nutrient-deprived conditions, they induce FAO and autophagy for energy production (8, 9). Green colour indicates a nutrient-rich condition, and the red colour indicates a nutrient-deprived condition [FA, ,Fatty acid; Ile, Isoleucine; Val, Valine; Leu, Leucine; mTOR, Mammalian target of rapamycin].

Cancer is a multifactorial disease and one of the leading causes of death globally. According to the World Health Organization (WHO), cancer was responsible for approximately 10 million deaths in 2020 (1). Disruption in normal cellular functions is a feature of all cancer cells (2). Dysregulated cellular metabolism is one of the important hallmarks, and cancer cells alter their metabolism to overcome the cancer-associated cellular stress, thereby leading to “metabolic reprogramming” (3, 4). Metabolic reprogramming refers to the alteration in catabolic, anabolic, and redox pathways of the cells supported by the tumor microenvironment. Otto Warburg first described metabolic alteration in cancer cells; while normal cells convert glucose to pyruvate in the presence of oxygen (aerobic glycolysis) and redirect the pyruvate towards the tricarboxylic acid (TCA) cycle (5), cancer cells abnormally preferred to convert glucose to lactate irrespective of oxygen availability (6). Under normal circumstances, the pathway intermediates from glycolysis are used in different anabolic pathways; glyceraldehyde-3-phosphate is redirected towards fatty acid synthesis, while glucose-6-phosphate and fructose-6-phosphate are used for nucleotide biosynthesis through oxidative Pentose phosphate pathway (PPP) and non-oxidative PPP, respectively (7). Such anabolic pathways are activated only in nutrient-rich conditions. However, a metabolic shift occurs in the nutrient-deprived state, wherein the cells activate different catabolic pathways that can supply the required glycolytic and TCA cycle intermediates for energy production (**Figure 1**) (8).

The amino acid metabolic pathway is the major contributor to these intermediates. Additionally, cancer cells also show upregulation of lipogenesis and harness fatty acid oxidation to meet the energy requirements during nutrient-deprived conditions. These metabolic changes vary during different stages of cancer progression, from tumor initiation to metastasis. Pavlova et al. have discussed the six hallmarks of cancer metabolism, typical to all types of cancers (10).

The metabolic shift from TCA cycle mediated energy generation to glucose fermentation allows cancer cells to accumulate mutations in the involved enzymes. Some of these enzymes also function as tumor suppressors and their loss of function mutations can drive tumor progression as in the case of Isocitrate dehydrogenase (IDH) 1 and 2 (11), Fumarate hydratase (FH) (12), and succinate dehydrogenase (SDH) (13).

Due to the high metabolic activity, cancer cells produce copious amounts of reactive oxygen species (ROS) (14). Low levels of ROS can promote cellular proliferation, whereas high ROS levels can damage DNA and activate apoptotic pathways (15). Cancer cells circumvent these deleterious effects of high ROS by upregulating the synthesis of various ROS scavenging proteins and NADPH (7).

Further metabolomic studies in cancer have revealed that cancer cell metabolism is heterogeneous and may vary depending on the site and origin of cancer (3). One such instance is the diverse effect of MYC on glutamine metabolism, wherein MYC induces glutamine synthesis in liver cancer but drives glutamine catabolism in lung cancer (16). The metabolic interactions between the cancer cells and tumor microenvironment (TME) introduces further intricacies in the role of these alterations (17). The symbiotic relationship between the cancer cells and various component of the TME creates a hospitable niche for tumor progression which suppresses the immune system and promotes survival and metastasis of cancer cells. Furthermore, availability of new positron emission tomography (PET) probes will enable characterisation of tumors on the basis of their active metabolic state, opening new possibilities of precision therapy (3). This chapter discusses the different metabolic signatures associated with various cancer types and how they may be targeted therapeutically.

## Alterations in glucose metabolism

Glucose is the primary source for ATP production and synthesis of various macromolecules. Under physiological conditions, glucose is catabolized into pyruvate *via* the Embden-Meyerhof pathway, producing ATP and NADH (18). The fate of pyruvate is further dependent on oxygen availability, wherein aerobic (oxygen-rich) conditions facilitate the conversion of pyruvate into acetyl-CoA by pyruvate dehydrogenase (PDH). In contrast, anaerobic conditions (lack of oxygen) facilitate production of lactate from pyruvate by lactate dehydrogenase (LDH). In normal cells, acetyl-CoA enters the mitochondria and partakes in the tricarboxylic acid (TCA) cycle, also known as the citric acid cycle, to generate NADPH, FADH2, and GTP. These reducing equivalents (NADH and FADH2) enter the electron transport chain (ETC) to produce ATP with the help of ATP synthase through oxidative phosphorylation (OXPHOS). However, this metabolic pathway is altered in cancer cells to aid in cancer progression. The rapidly dividing cancer cells circumvent the time-consuming route of energy generation *via* the TCA cycle and instead undergo aerobic glycolysis even under an oxygen-rich state (6). The increased lactate production even under normoxic conditions, often dubbed as Warburg effect or aerobic glycolysis, is a common feature of all types of cancers, including brain, breast, lung, colorectal, hepatocellular, gastric, bladder, and blood cancer (19–25), with the exception of prostate cancer, wherein aerobic glycolysis is observed only in the later stages of tumor progression (26–28). By limiting energy generation *via* TCA cycle, cancer cells control their high ROS levels, thereby limiting oxidative stress induced apoptosis (29).

NADPH and FADH2 produced in the TCA cycle are used to produce adenosine triphosphate (ATP) through oxidative phosphorylation (OXPHOS) in the electron transport chain under physiological conditions. However, cancer cells exhibit dysregulation of OXPHOS, perhaps to further reduce the oxidative stress. In breast cancer (BC), gastric cancer (GC), hepatocellular carcinoma (HCC), and non-small cell lung carcinoma (NSCLC), various mutations have been reported in the mitochondrial DNA, which codes for 13 subunits involved in the ETC. Furthermore, Hepatitis C virus (HCV) infection and dysregulation of mitochondrial microRNAs downregulate the expression of OXPHOS-associated proteins in HCC (30). In the HCV-induced HCC mice model, upregulation of stem cell homeobox transcription factor NANOG suppresses OXPHOS to promote self-renewal and drug resistance (31). Ras-like GTPase, Rab3A, and TGF-β have also been reported to downregulate OXPHOS and induce migration and invasion in HCC (31–33). However, recent studies have shown that some cancer cells also rely on mitochondrial respiration. For instance, high expression of mitochondrial biogenesis gene, Peroxisome proliferator-activated receptor-gamma coactivator-1α (PGC-1α), promotes aerobic respiration in invasive circulating breast cancer cells (34). Intriguingly, differentiating enterocytes also show increased expression of PGC-1α as they migrate from the intestinal crypts towards the surface (35). However, unlike other tissue types wherein increased expression of PGC-1α also upregulates antioxidant genes like superoxide dismutase 2 (SOD2) and catalase (CAT) to cope with the ROS burden coupled with mitochondrial respiration, PGC-1α does not induce the expression of antioxidant genes in intestinal cells which results in ROS-induced apoptosis, thereby maintains the intestinal tissue homeostasis (35). Colorectal cancer (CRC) cells predominantly express PGC-1β, which can induce the expression of antioxidant genes, thereby maintain low ROS levels and normal glycolytic pathway, resulting in prolonged lifespan and accumulation of these cells (36). Similarly, several other cancer types maintain their dependence on OXPHOS and show increased mitochondrial content, such as in leukaemia, and prostate cancer (37, 38).

Lactate dehydrogenase (LDH) is overexpressed in cancer cells and promotes the conversion of pyruvate into lactate (39). Overexpression of lactate transporters, monocarboxylate transporter (MCT) 1 and 4 is a common feature of cancer cells that allows efflux of high amounts of lactate produced by oxygen-deprived cells. The normoxic cancer cells uptake this lactate from the microenvironment owing to their high expressions of MCT1 and utilise it for energy production *via* the TCA cycle (40).

In colorectal cancer (CRC) cells, an increase in aerobic glycolysis and downregulation of the TCA cycle can be attributed to the reduction in mitochondrial import of pyruvate due to low expression of mitochondrial pyruvate carrier 1 (MPC1) (41). Furthermore, in CRC and bladder cancer, the mitochondrial pyruvate fails to undergo the TCA cycle due to the inhibition of pyruvate dehydrogenase (PDH) by the overexpressed pyruvate dehydrogenase kinase 4 (PDK4) (42–44). On the other hand, energy generation without the involvement of the TCA cycle allows cancer cells to utilise the TCA cycle components for proliferation and invasion (45). A study of human lung biopsies using stable isotope resolved metabolomics (SIRM) revealed changes in glycolysis and mitochondrial function in non-small cell lung cancer (NSCLC), wherein cancer cells showed higher levels of TCA cycle intermediates than the surrounding normal tissue cells (46). In HCC, downregulation of PDK4 promotes the conversion of pyruvate into acetyl-CoA by PDH (47). Enhanced production of oxaloacetate from pyruvate due to overexpression of pyruvate carboxylase is reported in LC cells, which results in increased production of downstream TCA cycle intermediates (48). Many of the TCA cycle moonlighting enzymes, such as Isocitrate dehydrogenase (IDH) 1 and 2, succinate dehydrogenase (SDH), and fumarate hydratase (FH), which function as tumor suppressors under physiological conditions, have a loss of function mutations in cancer cells. For instance, IDH1 and IDH2 convert isocitrate to α-ketoglutarate and 2-hydroxyglutarate, an inhibitor of histone demethylase and ten-eleven translocation (TET) proteins. In NSCLC, mutations in IDH1 and IDH2 result in various tumor-promoting epigenetic alterations (11).

Prostate cells pose another intriguing exception, where the TCA cycle is inhibited under the physiological condition to produce high amounts of citrate as a component of semen (49). The accumulation of citrate is attributed to high zinc concentration in the prostate cells, which inhibits the enzyme m-Aconitase required to convert citrate to isocitrate in the first step of the TCA cycle (49). Also, zinc acts as an anti-tumor/pro-apoptotic regulator by inducing the release of cytochrome-c from mitochondria (50, 51). This high zinc concentration is maintained by the upregulation of an ion channel called zinc or iron-regulated transporter like-protein 1 (ZIP1) in prostate cells (52). However, prostate cancer (PC) cells have atypical low zinc and citrate levels. In PC cell lines DU-145 and LNCaP, ZIP 1 channel is downregulated due to hypermethylation of its promoter at the binding site of transcription factor AP-1 (53). The resulting low zinc concentration allows completion of the TCA cycle by oxidising the citrate, increasing the energy output from 14 ATPs to 24 ATPs per glucose molecule. Therefore, unlike other cancers, the Warburg effect is not observed in the initial stages of PC.

Anaerobic glycolysis produces approximately 16-fold less energy per glucose molecule than its aerobic counterpart. To make up for this inefficiency, cancer cells have 15 times higher glycolytic flux than normal cells to meet their energy requirements (6, 24). The increased glucose demand is met by the upregulation of glucose transporters, such as GLUT2 in gastric and hepatocellular cancer and GLUT1 and GLUT3 in other cancer types (39). Upregulation of GLUT1 activity in CRC cells is attributed to dysregulation of RAS/MAPK pathway due to KRAS mutation (54). Similarly, CD147 upregulates GLUT1 in hepatocellular carcinoma (55). Furthermore, upregulation of hexokinase (HK) in cancer cells ensures retention of glucose by converting it into membrane impermeable glucose-6-phosphate (G6P) (39). Other key regulatory enzymes of the glycolytic pathway, such as phosphofructokinase (PFK), which converts G6P to fructose-6-phosphate (F6P), and pyruvate kinase (PK), which converts phosphoenolpyruvate to pyruvate are upregulated in cancer cells resulting in high glycolytic turnover (39, 56). Downregulation/deletion of sirtuin 6 (SIRT6), a known repressor of tumor driver MYC is associated with adenoma formation and increased glycolysis during all stages of colorectal adenomas (57, 58).

The increased glycolytic turnover is coupled with upregulation of the Pentose phosphate pathway (PPP) in many cancers. PPP provides pentose phosphate required for nucleic acid synthesis and NADPH needed for fatty acid synthesis and survival (59). Glucose-6-phosphate dehydrogenase (G6PD), which converts G6P to 6-phosphogluconate (6-PG), is the key regulatory enzyme for PPP and is upregulated in most cancers. Mammalian target of rapamycin 1 (mTORC1) and p21 activated kinase 4 (PAK4) modulates transcriptional and post-transcriptional regulation of G6PD and enhances Mdm2-mediated p53 ubiquitination and degradation. Furthermore, mutations in P53 and overexpression of Polo-like kinase 1 (Plk1) promote the dimerization and activation of G6PD in cancer cells. TP53-inducible glycolysis and apoptosis (TIGAR), which regulates the flux of glycolysis intermediates into PPP in normal injured tissue, is upregulated in CRC cells regardless of their P53 status and associated with the formation of adenomas (60). Similarly, the downstream PPP enzyme, 6-phosphogluconate hydrogenase (6-PGD), which converts 6-PG into ribulose-5-phosphate (Ru5P), is also upregulated in breast, lung, ovarian and blood cancers. Accumulation of PPP intermediates ribose-5-phosphate (R5P) and xylulose-5-phosphate is observed in various cancer types, particularly in HER2 positive BC. However, HCC is an exception where low levels of Ru5P and R5P are observed despite the upregulation of PPP enzymes (61, 62).

## Molecular basis of glucose metabolic reprogramming

The metabolic state of the cell dictates its fate and functions. Under physiological conditions, the cellular metabolism is tightly regulated by various growth factors, which signal the cells to uptake nutrients from the extracellular space. But cancer cells carry oncogenic mutations that render their signal transduction independent of growth factor-mediated stimulation.

PI3K/AKT/mTOR pathway is one of the prominent signaling pathways which contributes to the import of nutrients to the cell. PI3K/AKT/mTOR pathway is among the commonly dysregulated pathway and is actively involved in the regulation of cancer cell survival, proliferation, growth and metabolism (63). Tumor suppressor PTEN antagonizes this signaling pathway by dephosphorylating PIP3 which leads to inhibition of downstream proteins phosphoinositide-dependent protein kinase (PDK1), AKT1 and mTOR. In normal cells, activation of PI3K/AKT/mTOR pathway induces glycolytic flux upon stimulation by growth factors like insulin. This is frequently altered (mutation in components of PI3K complex or by hyperactivation of RTKs) in cancer cells leading to a dysregulation of glucose metabolism (64–66). Loss of PTEN is observed in glioblastoma, melanoma, endometrial and prostate cancer. This results in activation of the downstream target proteins of PI3K signaling pathway inducing expression of glycolytic enzymes like HK2, PFK1, and glucose transporter GLUT1 (67, 68). HK2 prevents release of apoptotic protein, cytochrome-c, by interacting with the mitochondrial pore to enable cell survival. Furthermore, AKT itself activates FOXO3a which promotes mitochondrial biogenesis (69, 70).

Oncogenes involved in these pathways are responsible for regulating the expression of glycolytic enzymes (71). HIF and c-Myc particularly, coordinate to promote glycolysis *via* activation of several glycolytic enzymes like hexokinase II (HK2), phosphopfructokinase I (PFK1), glyceraldehyde-3-phosphate dehydrogenase, enolase 1, pyruvate kinase, and LDH-A, and are known as master inducers of glycolysis (71–74). In normal cells, c-Myc is induced by growth factor stimulation, however, in cancer cells, it is aberrantly activated by gene mutations (single nucleotide polymorphism, chromosomal translocation) and induces energy production and anabolic processes even in absence of growth factor stimulation. To accomplish this, c-Myc induces expression of key glycolytic enzymes; it also increases the ratio of pyruvate kinase M2 (PK-M2) to pyruvate kinase M1 (PK-M1) by indirectly modulating exon splicing, thereby enforcing a shift toward lactic acid production, a prominent marker of cancer progression (75). c-Myc also induces NADPH production which further supports cancer proliferation (76).

**Figure 2:**
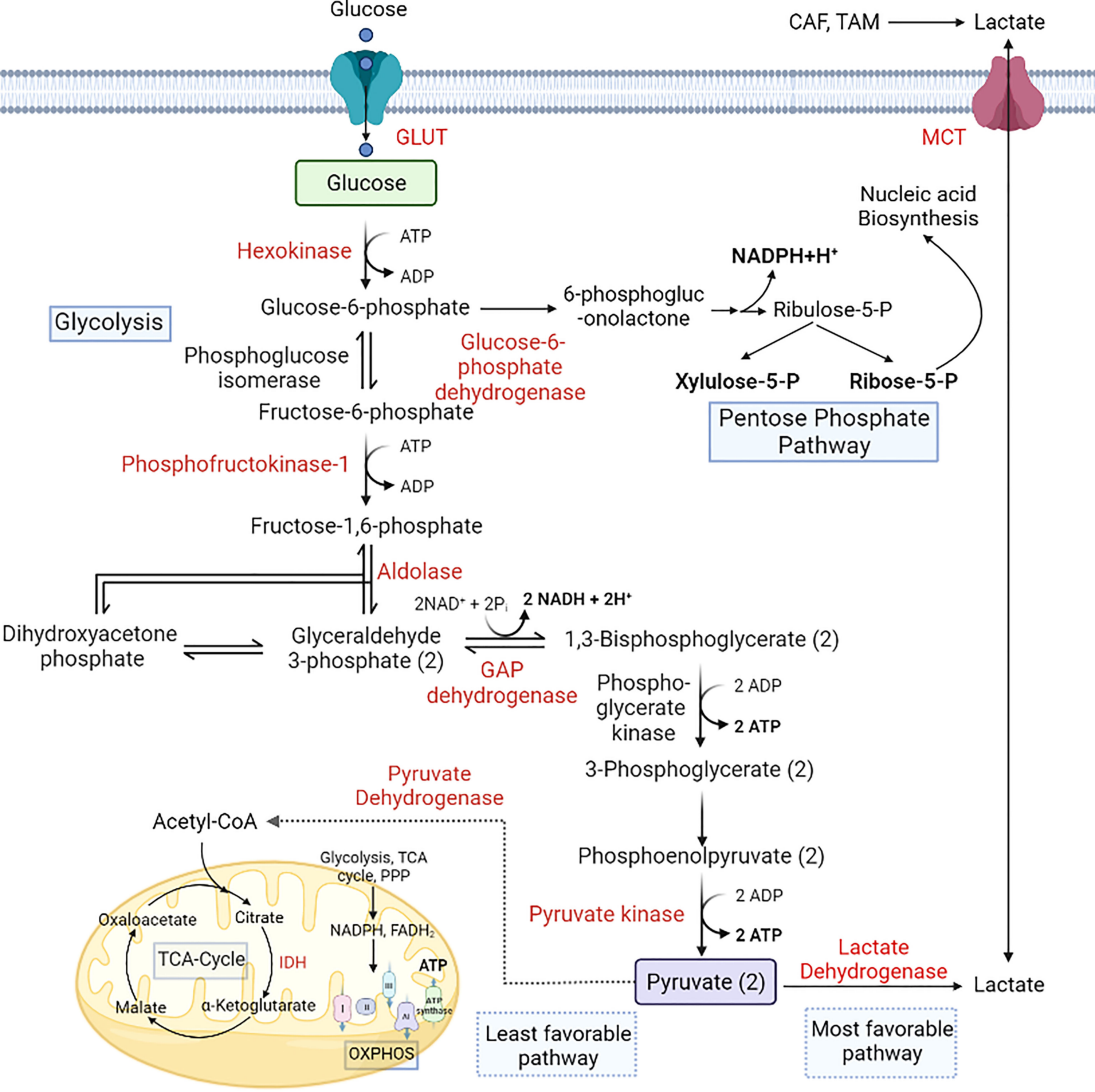
Alterations in glucose metabolism during cancer progression. Cancer cells utilize glucose as a primary energy source. GLUT overexpression helps the cells to uptake more glucose from the microenvironment, which is converted to lactate-by-lactate dehydrogenase. Lactate produced by the cells is secreted into the microenvironment *via* the upregulation of MCT1. PPP is also upregulated in cancer cells to produce NADPH and pentose phosphate for nucleic acid synthesis (18, 96, 97). [GLUT, Glucose transporter; IDH, Isocitrate dehydrogenase; MCT, Monocarboxylate transporter; OXPHOS, Oxidative phosphorylation].

Expression of hypoxia inducing factor-1 α (HIF-1α) is upregulated by PI3K and RAS/RAF/MEK/ERK kinase cascade (77–80). HIF-1α is stabilized by CREB binding protein (CBP)/p300 through ERK-mediated phosphorylation and also by ROS generated from ETC complex II and III (81–84). It induces the less efficient mode of glycolysis i.e., aerobic glycolysis, and also upregulates GLUT1 and GLUT3 expression resulting in uptake of glucose from the environment thereby increasing the rate of glycolysis in cancer cells (72, 85). It also upregulates PDK1 and LDH-A to prevent glucose flux into the TCA cycle thereby inhibiting OXPHOS and making pyruvate available for conversion into lactate by LDH-A (86, 87). Wnt signalling also acts as a driver of cellular proliferation in CRC by modulating the expression of PDK and inhibiting TCA cycle (88, 89). It is also a known driver for the upregulation of MCT1 in CRC (90). In tumor cells, elevated levels of c-Myc and HIF-1α, coupled with loss-of-function mutations in P53 leads to uncontrolled cell division, inhibition of apoptosis and cancer progression. P53, in normal cells, suppresses Warburg effect by decreasing glycolysis through repression of HK2 and glucose transporter (GLUT1, GLUT3) expression (91, 92). However, in cancer, P53 not only loses its function but it gains an oncogenic function (mutp53) which can inhibit AMPK and upregulate glucose transporters, GLUT1, GLUT3, and GLUT4, to proliferate under energy deficient conditions (93, 94). HIF-1α and HIF-2α are overexpressed in many types of cancers. In BC and high-grade bladder cancer, HIF-1α upregulates the expression of 6-phosphofructokinase/fructose-2,6-bisphosphatase (PFKB) 3 and 4, and controls the overall glycolysis rate by modulating the activity of phosphofructokinase 1 (PFK1) (95). This highlights the centrality of HIF-1α and c-Myc in the metabolic landscape of the tumor cells and their progression. c-Myc also plays a key role in glutamine metabolism and is responsible for glutamine addiction in cancer cells (**Figure 2**).

## Dysregulations in amino acid metabolism

Amino acid metabolism is closely intertwined with the glycolytic pathway, where the amino acid pools can generate various components of the TCA cycle *via* anaplerotic pathways, along with other metabolites such as glucose, lipids, and precursors of purines and pyrimidines (98). Cancer cells utilize their amino acid pools during glucose deprivation to meet their energy requirements. Glutamine is the primary amino acid crucial for cancer proliferation and provides carbon and nitrogen that supports biosynthesis and cellular homeostasis in cancer cells (98). Cancer cells maintain high pools of glutamine by upregulating the expression of glutamine transporters, such as Alanine/Serine/Cysteine/Threonine Transporter 2 (ASCT2; SLC1A5) in PC and SLC7A5 in HC. Furthermore, glutamine synthetase (GS) that converts glutamate to glutamine is overexpressed in HCC and acts as a diagnostic biomarker which is correlated with more aggressive disease (99). Chronic hepatitis B (CHB) also leads to HCC and high expression of GS is also observed in CHB stage 1-4 (100). Glutamine synthase produced by glial cells also converts ammonia to glutamine in glioblastoma tissues thus providing an alternate source of glutamine (101). Glutamine depletion *via* shRNA-mediated silencing of ASCT2 inhibits tumor formation (102) and is a candidate for targeting amino acid dependence in cancer cells. In many cancers, including BC, LC, CRC, PC, GBM, HCC, GC, and leukaemia, Glutamine is used to produce glutamate by the process of glutaminolysis, which is later converted into TCA component α-KG by glutamate dehydrogenase (GDH) (62, 98, 103–107).

The upregulation of glutaminolysis in cancer cells can be attributed to increased activity of glutaminase 1 (GLS1) enzyme. Overexpression of TGF-β increases the levels of GLS1 in HCC (32) and in T-cell acute lymphoblastic leukaemia (T-ALL), increased glutaminolysis induces NOTCH1 signaling, promoting growth and survival of cancer cells (6, 107). N-methyl D-aspartate-associated protein 1a (GRINA), a glutamate receptor in GC cells, modulates aerobic glycolysis and is also involved in lipid and sterol synthesis, which ultimately promotes tumor progression (108, 109). A higher glutamine level further activates mTORC1, which helps in protein translation and nucleic acid biosynthesis required for cell growth and proliferation (110). On the other hand, amino acid deprivation in tumor cells promotes autophagy and cell survival *via* the mTOR pathway (111). In glutamine-deprived conditions, upregulation of KRAS and asparagine synthetase (ASNS) induces asparagine synthesis from aspartate, thereby increasing the growth and proliferation of CRC (112). Asparagine can serve as an antiporter for the influx of other amino acids and induces the mTOR pathway during amino acid deprivation (113). Leukaemia and HCC have low levels of ASNS leading to asparagine deficiency, which rationalised the use of L-asparaginase, which converts asparagine into aspartate as an adjunct therapy (114, 115). Karpet-Massler et al. identified that L-asparaginase derived from E. coli decreases the cell growth in glioblastoma (116). However, as reviewed by Jiang et al., asparagine depletion further reduces the transcription of ASNS in a feedforward manner *via* p53 activation in tumor cells, warranting further studies on the role of p53 mutations in determining the efficacy of L-asparaginase therapy (113).

Serine and glycine are also noteworthy as they provide essential precursors for synthesising proteins, nucleic acids, and fats required by the cancer cells (117). In LC, upregulation of Na^+^-dependent transporter, ASCT1 leads to increased serine uptake (98). Serine acts as a precursor for nonessential amino acids, glycine, and cysteine. It is also involved in the production of sphingolipids and supplies carbon to the one-carbon pool required for folate metabolism. The folate-methionine route is responsible for a variety of processes involving volatile carbons, such as the interconversion of serine and glycine and the creation of thymidine. Tetrahydrofolate, generated from folic acid, is a flexible carbon donor that can transport a range of one-carbon functional groups, such as methyl, methylene, and formyl groups. This property makes it a versatile cofactor in biosynthetic pathways. S-adenosylmethionine, a derivative of methionine, serves as another methyl donor. This metabolic route establishes a strong functional link between cellular metabolism and epigenetic regulation, which is essential for DNA methylation. The growth of glioma cells is restricted in the absence of methionine (118). Methylation by histone methyltransferase (HMT) and DNA methyltransferase (DNMT) is associated with poor prognosis in CRC. Puccini et al. reported overexpression of S-adenosylmethionine in all stages of CRC, which acts as a co-substrate for HMT and DNMT (119). Upregulation of the serine-glycine biosynthetic pathway due to overexpression of phosphoserine aminotransferase 1 (PSAT1) is associated with higher tumor proliferation and poorer prognosis in CRC and BC (120, 121). Furthermore, downregulation of ζ isotype of protein kinase C (PKCζ) expression promotes the serine biosynthesis *via* increased activity of enzymes phosphoglycerate dehydrogenase (PHGDH) and PSAT1, leading to higher intestinal tumorigenesis. In addition, low expression of PKCζ in intestinal tumors correlates with poorer prognosis (122).

Similarly, arginine is a semi-essential amino acid therapeutically relevant in PC, HCC, and leukaemia, owing to the arginine auxotrophy of cancer cells. This dependency is due to the downregulation of key enzymes involved in arginine synthesis, such as arginosuccinate synthetase 1 (ASS1), ornithine transcarbamylase (OTC) and carbamoyl-phosphate synthetase 1 (CPS1) (114, 123, 124), which catalyses the conversion of citrulline to arginine *via* the ornithine cycle in normal cells. Arginine depletion using arginase (125) or pegylated arginine deiminase (126) induces cytotoxicity in prostate cancer cell lines. Arginine depletion also increases the efficacy of drugs such as docetaxel to treat prostate cancer (127).

**Figure 3:**
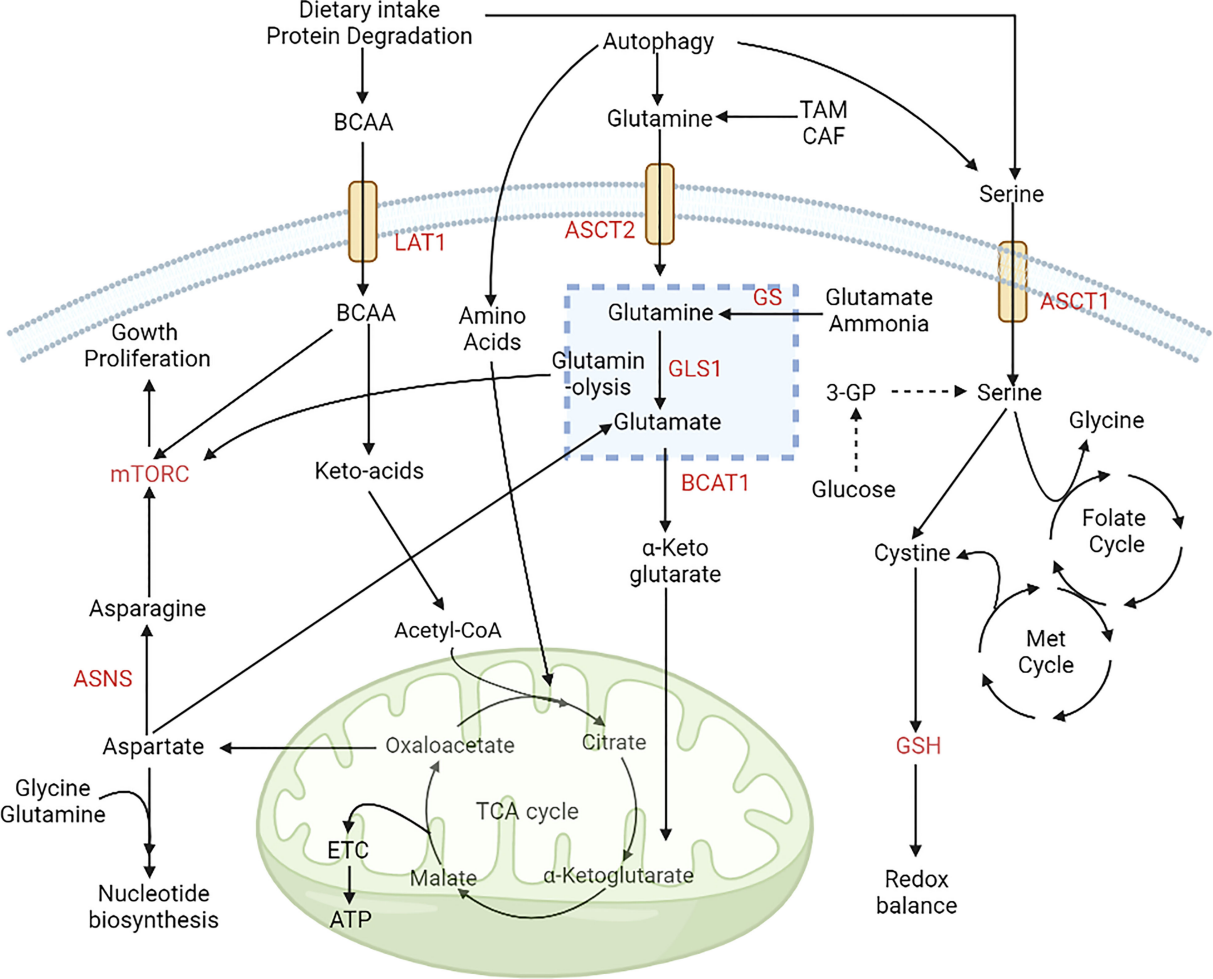
Alterations in amino acid metabolism during cancer progression. Amino acids are used by cancer cells as a source of carbon and nitrogen. The expression of ASCT2 increases glutamine uptake, and it is converted to glutamate *via* glutaminase and provides a TCA-cycle intermediate for energy production. Intake of BCAAs is also upregulated by the expression of LAT1. The folate methionine cycle is fuelled by serine, which helps maintain redox balance in the microenvironment. These alterations aid the growth and proliferation of cancer cells *via* upregulation of the mTOR signaling pathway (98). [LAT1, Large amino acid transporter 1; ASCT2, Alanine serine cysteine transporter 2; BCAA, Branch chain amino acids; BCAT1, Branch chain aminotransferase 1; ASNS, Asparagine synthetase; ETC, Electron transport chain; GSH, Glutathione; Met, Methionine; 3PG, Glyceraldehyde 3 phosphate].

Moreover, large neutral amino acid transporter 1 (LAT1 or SLC7A5), the primary transporter of branched-chain amino acids (BCAAs) that are not synthesised in the body, is also overexpressed in lung cancer, glioblastoma and the blast phase of chronic myeloid leukaemia (98), providing the nutrients necessary for tumor growth (101). Furthermore, the upregulation of branched-chain aminotransferase 1 (BCAT1) expression by H3K9 demethylation drives cancer progression by increasing the synthesis of branched-chain amino acids like valine, leucine, and isoleucine (128) and increases ROS scavengers and imparts chemoresistance against tyrosine kinase inhibitors (129). High cysteine levels increase the proliferation and chemoresistance in CML cells by maintaining cellular redox balance (114, 130). In CRC cells, upregulation of cystathionine-β-synthase (CBS) promotes proliferation (by upregulating glycolysis, PPP, and lipogenesis), invasion, and anoikis resistance by catalysing the condensation of homocysteine and cysteine to produce hydrogen sulphide (131). The CRC-specific biomarker CD110 also has an essential role in lysine catabolism and activates the Wnt signaling pathway by upregulating acetylated low-density lipoprotein receptor related-protein 6 (LRP6) and glutathione (132, 133). Tryptophan is an essential amino acid which is used by the immune cells, and tryptophan level decreased in the TME due to higher expression of indoleamine 2,3-dioxygenase 1 (IDO1). Lack of tryptophan leads to T-cell apoptosis or inhibit immune cell proliferation *via* downregulating mTOR signaling pathway and activating nonderepressible 2 (GCN2) (134, 135). GCN2 not only downregulate protein synthesis and T cell proliferation it can also induce differentiation of naïve T cells to T regulatory cells which ultimately create an immunosuppressive microenvironment (136). BC patients, particularly TNBC, show upregulation of tryptophan pathway metabolite Kynurenine, which promotes cancer progression by creating an immunosuppressive microenvironment (137, 138) (**Figure 3**).

## Amino acid metabolism and autophagy

The inhibition of mTORC1 under nutrient-deprived conditions allows the cells to undergo autophagy, wherein macromolecules are recycled *via* autophagosome mediated lysosomal degradation (139). The Amino acid sensor proteins play a major role in regulation of mTORC1 activity which further modulates cellular metabolism, growth and survival (140). Rag GTPase is a regulator of mTOR that is controlled by different amino acid sensor proteins. Sestrin is the first identified amino acid sensor (response to cytosolic leucine levels) that regulates Rag GTPase, hence downregulating mTORC1 activity (141). Since, macropinocytosis is inhibited by mTORC1, sestrin-mediated inhibition of mTORC1 leads to increased import of extracellular macromolecules and therefore, increases survival of cells under low-nutrient conditions (142). Leucyl-tRNA synthetase (LARS) is an intracellular leucine sensor which interacts with Rag GTPase and activates mTORC1 activity (143). SLC38A9, a lysosomal transmembrane protein, which acts as an intra-lysosomal amino acid sensor for leucine, glutamine, tyrosine, and phenylalanine, also activates mTOR by Rag-GTPase (144–146). CASTOR 1/2, a GATOR2 binding protein, inhibits mTORC1 activity by forming homo- or hetero-dimer upon sensing arginine (147). s-adenosyl methionine (SAM), upon methionine sensing, induces S-adenosylmethionine sensor for the mTORC1 (SAMTOR) and GATOR1 dimerization leading to downregulation of mTORC1 (148). Intracellular amino acid-activated mTORC1 itself regulates amino acid availability and also phosphorylates S6 kinase 1 (S6K1) and eukaryotic translation initiation factor 4E binding protein 1 (4EBP1) which ultimately increase translation of metabolic enzymes and transcription factors (149).

Thus, the dysregulated amino acid metabolism and the amino acid auxotrophy in several cancer cells can be exploited for therapeutic purpose.

## Alterations in lipid metabolism

Lipid metabolism is instrumental for synthesising structural and functional lipids and generating energy during nutrient deficiency. Cancer cells generate fatty acids (FA) by lipogenesis during nutrient-rich conditions and use these stores as an alternate energy source during nutrient deprivation (150, 151). Fatty acid, cholesterol and lipids are primarily obtained from dietary intake, and *de-novo* synthesis of these molecules are restricted to the liver and adipocytes. However, the cancer cells have cholesterol *de-novo* synthesis, making them independent of extrinsic sources. In CRC, the expression of FASN increases in later stages (stage III, IV> stage I), which upregulates lipogenesis, mitochondrial respiration, and FA oxidation (152). For this, high amounts of FA are generated by the upregulation of one carbon metabolism resulting in increased proliferation of cancer cells (153).

Lipid metabolism is controlled by different oncogenes and tumor suppressors such as EGFR, PI3K, MAPK, Myc, and P53 (154, 155). 3-hydroxy-3-methylglutaryl-CoA reductase (HMGCR), a key regulatory enzyme in cholesterol biosynthesis (mevalonate pathway), is also upregulated in BC, GC, and HCC (156, 157). Enzymes involved in mevalonate pathway are upregulated in BC leading to the malignant transformation of benign epithelium (158–160). HMGCR is also responsible for the upregulation of Hedgehog signaling pathway - which plays a key role in tumor growth and proliferation - in GC (161). Additionally, HMGCR and not HIF, was observed to modulate YAP activation leading to chemoresistance in HCC under hypoxic conditions (158). Sterol regulatory element binding protein (SREBP) is a transcription factor which regulates genes involved in lipid synthesis. It has two variants: sterol regulatory element binding protein (SREBP1) which helps in lipid and fatty acid synthesis and energy generation, and sterol regulatory element binding protein 2 (SREBP2) which helps in cholesterol regulation (162). SREBP1 is the master regulator of fatty acid synthesis and controls the expression of FASN; both are regulated by PI3K-AKT and MAPK pathway (163, 164). SREBP1 is upregulated in PC, HCC and glioblastoma. Normally, SREBP1 activity depends on the intracellular cholesterol level; under high cholesterol conditions, SREBP1 remains attached to the endoplasmic reticulum (ER), but when intracellular cholesterol is low, SREBP1 is translocated to the Golgi apparatus and is activated. Activated SREBP1 upregulates lipogenic enzymes under the control of PI3K/AKT/mTOR signaling (156, 157, 165, 166). Brain is a cholesterol rich organ and consists of 20-25% of total body cholesterol, all of which is synthesised *de novo* by the astrocytes (167, 168). This characteristic is utilised by GBM cells where upregulation of lipogenesis due to high SREBP1 expression promotes their survival under hypoxic and lipid-deprived conditions and is associated with poor prognosis (169). Furthermore, dysregulated cholesterol synthesis in GBM cells also leads to high cholesterol availability for the invasive cells (89, 170). This dependence on lipid synthesis by SREBP1 for cellular growth in GBM is demonstrated by silencing sterol o-acyltransferase 1 (SOAT1) which converts ER cholesterol to cholesterol esters leading to inhibition of SREBP1 activation and therefore suppressed growth (166).

Fatty acids are the primary source of energy production for glioblastoma cells, and their ability to cross the blood-brain barrier is an advantage for invasive cancer cells (171). In hypoxic conditions, glioblastoma cancer stem cells increase their FA uptake *via* upregulation of fatty acid transporter CD36 (172). Grube et al. and Lancaster et al. identified that inhibition of FA synthesis or β-oxidation decreases glioblastoma and neural stem cell proliferation and can be a target for treatment (173, 174).

Fatty acid synthase (FASN), the key regulatory enzyme of lipogenesis, and acetyl-CoA carboxylase (ACC), are upregulated in many cancer types. Expression of FASN is also controlled by Sp/KLF family transcription factor (in prostate cancer), p53 and lipogenesis related nuclear protein SPOT14 (175–177). Post-translational regulation of FASN is observed in prostate cancer by the isopeptidase, ubiquitin-specific protease-2a (USP2a), which removes ubiquitin from FASN preventing its degradation (178). FASN and ACCα expression are regulated at the translational level by PI3K-mTOR signaling pathway in HER2 overexpressing breast cancer cells (BT-474, SK-BR-3) (179). In lung adenocarcinoma, it was observed that ACC expression is regulated by LKB1-AMPK pathway (140). ACCα expression is induced by IGF-1 in colon cancer cells, but it is suppressed by ERK1/2 dependent signalling pathway (180). Stearoyl-CoA-desaturase (SCD) is another factor that is upregulated in PC and GC, which helps convert saturated fatty acids to unsaturated fatty acids (165, 181).

In nutrient-deprived conditions, cancer cells use free fatty acids for energy generation *via* fatty acid oxidation (FAO). Carnitine palmitoyl transferase (CPT) helps transport these free fatty acids to the mitochondria and undergo FAO. Peroxisome proliferator activator γ (PPARγ) is upregulated during cancer progression, which induces fatty acid trafficking and energy generation. In low-nutrient conditions, CRC cells upregulate the AMPK pathway to promote autophagy and mitochondrial FA oxidation (FAO). AMPK pathway activation inhibits lipogenesis *via* downregulation of acetyl CoA-carboxylase (ACC) and upregulates carnitine palmitoyl transferase 1 (CPT1) (182). Carnitine palmitoyl transferase 2 (CPT2), an isoform of CPT1, is also upregulated in leukaemia, PC, CRC, and HCC. Upregulation of FAO in CRC *via* overexpression of carnitine palmitoyl transferase 1A (CPT1A) decreases the ROS level and provides anoikis resistance (183) [Cell death due to loss of adhesion, called anoikis, is a major hurdle for cancer cells during metastasis]. ATP-citrate lyase (ACLY), which converts citrate to acetyl-CoA, is overexpressed in LC and HCC cells and acts as a bridge between glycolysis and fatty acid metabolism (184).

**Figure 4:**
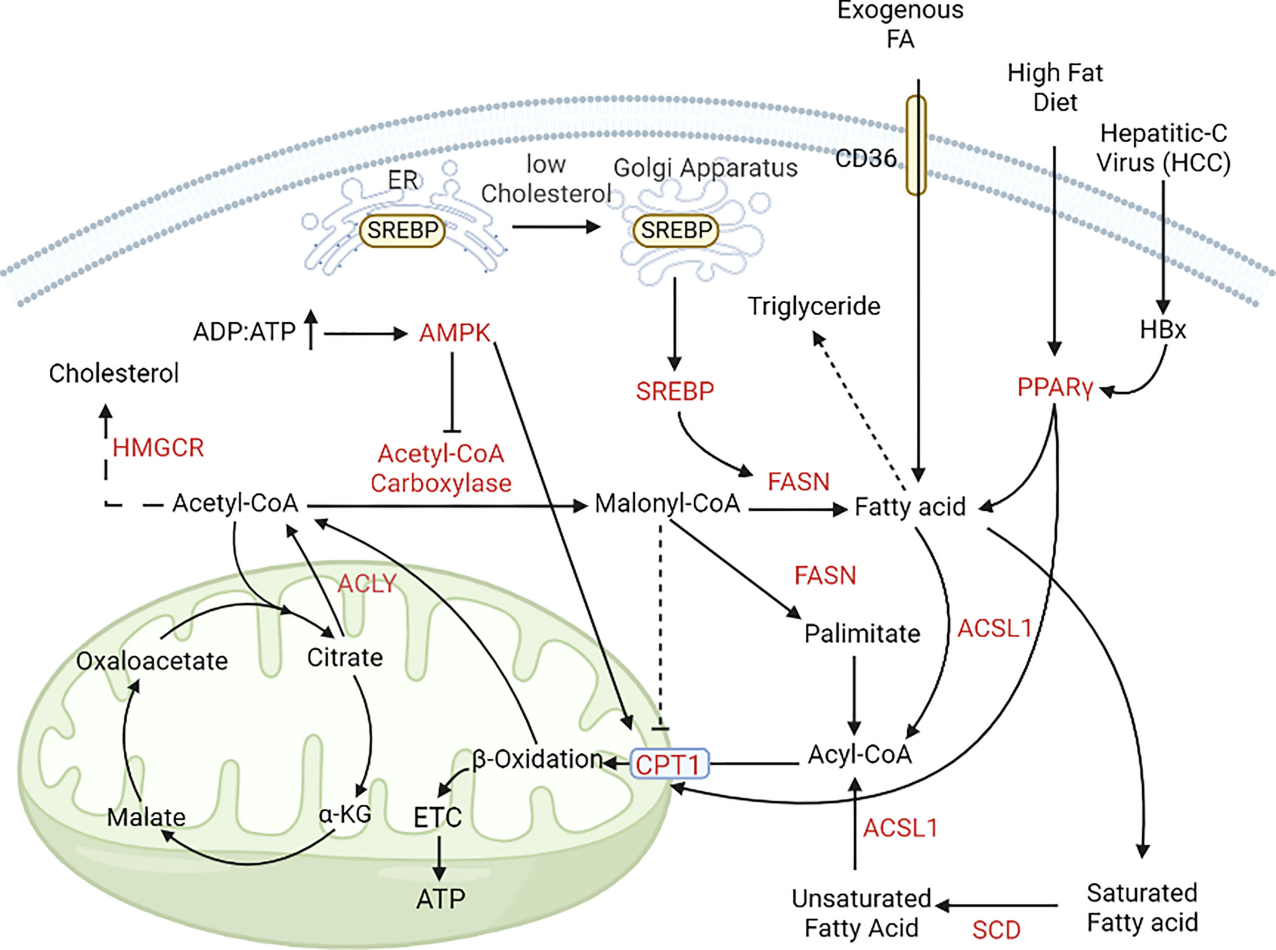
Alterations in lipid metabolism during cancer progression. In cancer cells, lipogenesis and lipolysis is controlled by the pool of ADP and ATP. In nutrient-rich conditions (ADP, ATP ratio is low); acetyl, CoA is converted to free fatty acids with the help of acetyl, CoA carboxylase and FASN. In nutrient-deprived conditions (ADP, ATP ratio is high), free fatty acids enter the mitochondria with the help of CPT1 and CPT1A and help in energy generation (195, 196). [FASN, Fatty acid synthase; CPT1, Carnitine palmitoyl transferase 1; CPT1A, Carnitine palmitoyl transferase 1A; GLUT, Glucose transporter; PPARγ, peroxisome proliferator activator receptor γ].

Dietary consumption of fats can also affect CRC tumorigenesis. A high-fat diet (HFD) induces PPAR-δ mediated activation of β-catenin target genes, which results in increased proliferation of intestinal stem cells (ISC) and expansion of TIC (tumor-initiating cells) (185). HCC caused by hepatitis-B virus (HBV) infection has higher levels of HBV protein HBx, which induces lipid accumulation in both mouse model and liver cell lines due to the increased expression of SREBP1 and PPARγ (186, 187). Breast cancer shows alterations in lipid metabolism depending on the type of BC. BC cells have increased catabolism of triglycerides and anabolism of linoleate, palmitate, and oleate. In the basal subtype BC, increased accumulation of monoacylglycerols *via* upregulation of monoacylglycerol lipase (MGAL) is crucial for epithelial to mesenchymal transition (EMT) and cancer progression (188, 189). Whereas, in the case of HER2, basal and luminal B subtypes, there is an accumulation of free fatty acids, palmitoyl carnitine, stearolycarnitine, and oleoyl carnitine. Additionally, accumulation of fatty acid oxidation product 3-hydroxybutyrate (3-HBA) is also reported in BC. The overall increase in FA anabolism promotes BC progression and cell survival. A high level of FA alters phospholipid biosynthesis and metabolism, which helps in the progression of HER2 and triple-negative breast cancer (TNBC) subtypes. Phosphatidylinositol 4-phosphate 5-kinase (PIPKIN) and phosphatidylcholine-specific phospholipase-C (PC-PLC) regulate FA metabolism and are overexpressed in all types of breast cancer, prominently in the TNBC (190–192). In GC, lysophosphatidic acid is converted to phosphatidic acid with the help of lysophosphatidylcholine acyltransferase 1 (LPCAT1) which correlates with tumor depth and lymph node metastasis (193). Nuclease receptor subfamily 1 group D member 1 protein (Rev-erbα) regulates lipid metabolism and decreases GC progression by augmenting glycolysis in GC (194) (**Figure 4**).

## Alterations in nucleotide metabolism

Purine and pyrimidine metabolism are also altered in cancer cells. There are mainly two pathways by which purines and pyrimidines are synthesized – *de novo* synthesis which is the main source of nucleotides *in vivo*, and the salvage pathway which is a shortcut used by those cells which do not possess all of the enzyme machinery necessary for purine nucleotide synthesis from scratch (brain and bone marrow). Salvage pathway is primarily regulated *via* negative feedback (197–202). Key regulatory enzymes which control purine nucleotide metabolism are 5’-phosphoribosyl-1’-phosphate synthetase (PRS), glutamine phosphoribosyl pyrophosphate amido-transferase (GPRATase), IMP hypoxanthine dehydrogenase (IMPDH), adenyl succinate synthetase (ADSS) and key enzymes for pyrimidine nucleotide metabolism are carbamoyl phosphate synthetase II (CPSII) and dihydroorotate dehydrogenase (DHODH) (203–208). Pavlova et al. reported that increased nitrogen demand is one of the hallmarks of cancer cell proliferation which is fulfilled by synthesizing essential nitrogen-containing molecules like nucleotides (4, 10). Purine and pyrimidine nucleotides are raw materials that support cellular proliferation which is dysregulated in cancer cells to enhance the proliferation and progression of cancer cells (209). c-Myc induces increased expression of nucleotide synthesis pathway genes like carbamoyl phosphate synthetase/aspartyl transcarbamylase/dihydroorotase (CAD), thymidylate synthase (TS), inosine-5’-monophosphate dehydrogenase (IMPDH) expression (210–212). p53 mutation and pTEN loss result in mTORC1 activation which induces one-carbon metabolism and purine and pyrimidine synthesis *via* phosphorylation of S6K and transcription factor E2F1 (213, 214). S6K can activate CAD *via* phosphorylation on ser^1859^, and E2F1 can induce the expression of TYSM which codes for TS (Pyrimidine anabolic), TK, and DPYD (Pyrimidine catabolic) (215, 216). Santana-Codina et al. identified that in pancreatic cancer KRAS drives tumor growth by activating pyrimidine nucleotide synthesis (217). Overexpression of CAD leads to poor clinical outcomes in BC, liver cancer, and CRC (218). Yu et al. identified higher expression of DHODH in Myc-amplified neuroblastoma and its inhibition led to suppressed neuroblastoma growth in animal models (219). Kollareddy et al. reported that mutant p53 (mutP53) can promote nucleotide metabolism genes, IMPDH and GMPS (220). mutP53 is stabilized by ubiquitin-specific protease 7 (USP7) which is regulated by nucleotide biosynthetic enzyme, guanosine 5’-monophosphate synthase thereby forming a feedback loop (GMPS) (220). Therefore, targeting different pathways of purine and pyrimidine nucleotide synthesis could be an effective strategy to increase the efficacy of cancer treatment.

## Crosstalk between metabolic pathways in cancer cells

Glucose, amino acid, lipid, and nucleotide metabolism are linked to each other which is utilized by the cancer cells to support unlimited growth and progression. During initiation phase cancer cells mainly use glucose as a primary source of energy and produce high amount of lactate. Anaerobic glycolysis produces less amount of energy instead of aerobic respiration but cancer cells increase the rate of anaerobic glycolysis by upregulating glycolytic enzymes HK, PFK, LDH and inhibit aerobic glycolysis by upregulating PDK4 which inhibits the conversion of pyruvate to acetyl-CoA and push the pyruvate to produce more lactate. This transition from OXPHOS to aerobic glycolysis happens mainly due to hypoxic conditions resulting from less vascularization (221). Altered expression of P53, Myc, HIF-1 and activation of PI3K/AKT/mTOR in cancer are the key drivers of aerobic glycolysis (222). Here, HIF-1 act as a master regulator which can sense the oxygen concentration in the microenvironment and crosstalk with other signaling pathways (223, 224). During cancer progression when glucose is deficient in the environment, cancer cells use other sources of carbon and nitrogen (amino acid, lipid, nucleotides) to produce TCA cycle intermediates and use OXPHOS for energy production. Therefore, metabolic plasticity of cancer cells allows them to switch or simultaneously use OXPHOS and glycolysis as per the need (223, 225, 226). OXPHOS is also observed in normoxic cancer cells which use lactate produced by the oxygen deprived cells, also known as reverse Warburg effect (23, 24).

Amino acids and lipids are the secondary source of energy which is used by the cancer cells in nutrient deprived condition. Glutamine, serine, and branch chain amino acids (leucine, valine, and isoleucine) produce TCA cycle intermediates α-ketoglutarate and pyruvate respectively, which produce NADH and FADH2 and undergo OXPHOS to produce ATP. In nutrient deprived condition, Myc binds to promoter of glutamine transporter SLC1A5 to increase glutamine uptake from the microenvironment, and increases GLS1 expression by suppressing the expression of microRNA miR-23a/b, leading to increased glutaminolysis (227, 228).

Flux of glucose to PPP pathway depends on glucose-6-phosphtae dehydrogenase (G6PD) which converts glucose-6-phospgate (a glycolytic intermediate) to 6-phosphogluconate (229). Expression of G6PD depends on the expression of PTEN, P53, AMPK which are mutated in most of the cancer types and increase flux of glucoe-6-phosphate to PPP and production of ribose-5-phosphate (substrate for purine metabolism) (59, 230). Nucleotide metabolism is also linked to PPP (ribose-5-phosphate) and amino acid metabolism (glutamine) which is required by the cancer cells to support DNA synthesis required proliferation and survival (231).

In nutrient replete conditions cancer cells undergo lipogenesis to produce lipid molecules from acetyl-CoA generated from acetate, glucose and glutamine, but during nutrient deprived condition lipolysis occurs to produce free fatty acids by breaking down lipid droplets which then undergo β-oxidation to produce energy for cancer cell survival and proliferation (232–234).

**Figure 5:**
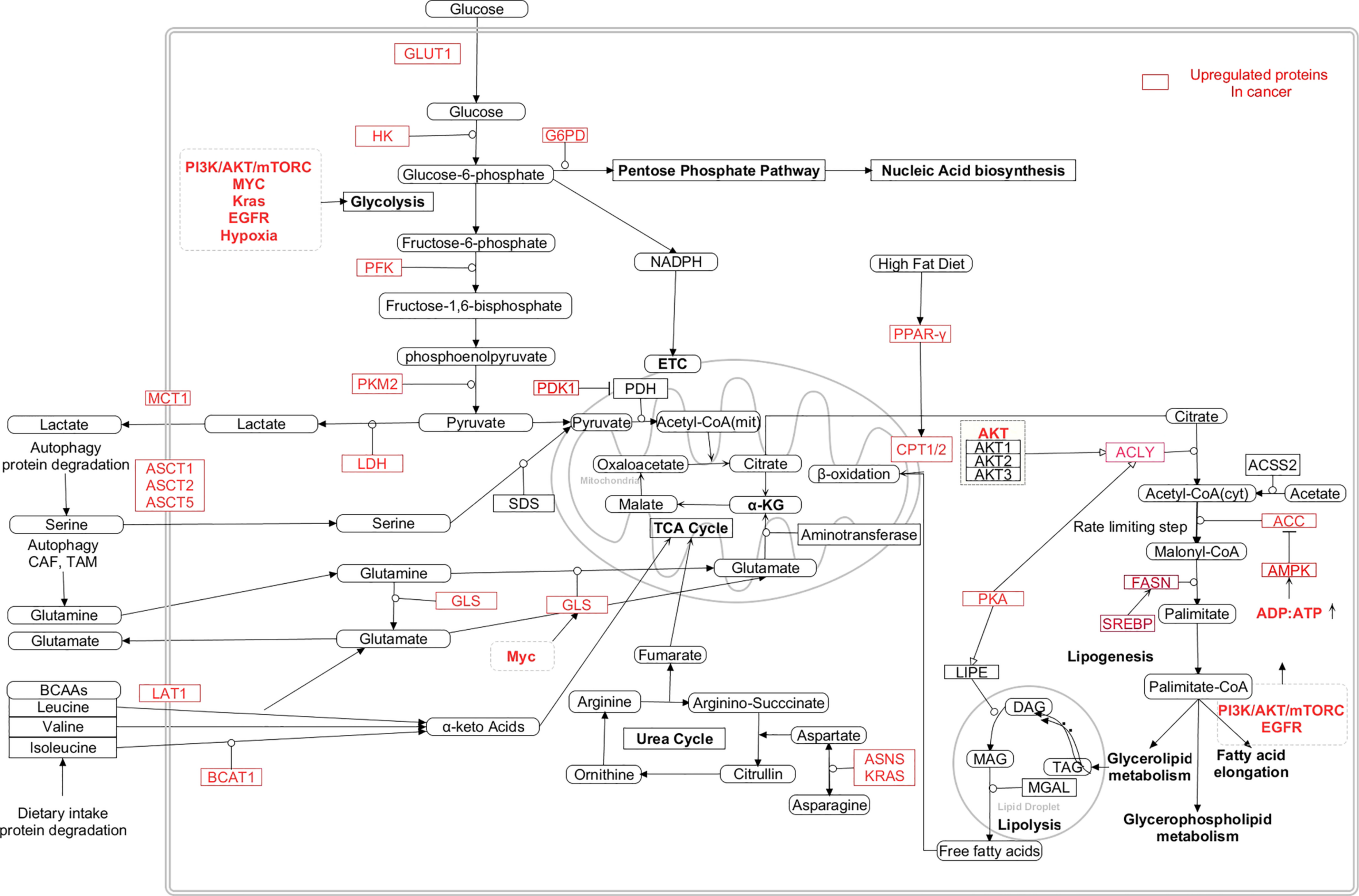
Interaction between the metabolic pathways and the dysregulated metabolic intermediates in cancer cells (24, 235–241). [HK: Hexokinase, PFK: Phosphofructokinase, PKM2: Pyruvate kinase M2, LDH: Lactate dehydrogenase, PDK1:Pyruvate dehydrogenase kinase 1, PDH: Pyruvate dehydrogenase, GLUT1: Glucossetransporter1, MCT1: Monocarboxylate transporter 1, ASCT: Alanine/Serine/Cysteine/Threonine transporter, LAT1: Large neutral amino acid transporter 1, GLS: Glutaminase, ASNS: Asparagine synthase, BCAT1: Branch chain aminotransferase 1, α-KG: α-ketoglutarate, BCAA: Branch chain amino acids, FASN: Fatty acid synthase, ACYL: ATP citrate lyase, SREBP: Sterol regulatory element binding protein, PKA: Protein kinase A, ACC: Acetyl-CoA carboxylase, TAG: Triacylglycerol, DAG: Diacylglycerol, Mag: Monoacylglycerol, PPAR-γ: Peroxisome proliferator activator receptor gamma, ASNS: Asparagine synthetase, CAD- Carbamoyl phosphate synthetase/aspartyl transcarbamylase/dihydroorotase, DHODH- Dihydroorotate dehydrogenase, PRPP- Phosphoribosyl diphosphate, IMPDH- IMP hypoxanthine dehydrogenase, GMPS- GMP synthetase, IMP- Inosine monophosphate, XMP-Xanthopsin monophosphate, GMP-Guanosine monophosphate, AMP-Adenosine monophosphate, UMP-uracil monophosphate, TMP-Thymine monophosphate].

Due to these interlinking nodes between glucose, amino acid, lipid, and nucleotide metabolism, when one metabolic pathway is interrupted due to nutrient deficiency or other external stress, cancer cells compensate by upregulating other metabolic pathways to support their growth and proliferation. Therefore, targeting single metabolic pathway cannot be an effective therapeutic option for cancer treatment and focus should be shifted on combination therapy wherein multiple metabolic pathways which are upregulated in cancer cells are targeted simultaneously (**Figure 5**).

## Metabolic reprogramming in tumor-microenvironment

### Stromal cells

The tumor microenvironment plays a critical role in tumor progression as the cancer cells evolve beyond the initial proliferative stage into metastatic stages. The bidirectional crosstalk modulates the metabolic reprogramming of cancer cells and various stromal and immune cells present in their microenvironment. Cancer-associated fibroblasts (CAFs), adipocytes, and immune cells in the CRC microenvironment are involved in tumorigenesis (242). Cancer cells are known to induce an “activated” state in fibroblasts, either by secretion of various growth factors and cytokines or *via* direct cell-cell contact mediated by Notch1. This transformation of fibroblasts into cancer associated fibroblasts (CAFs) is akin to activation of quiescent fibroblasts into proliferative myofibroblasts in the advent of tissue injury (243). CAFs can constitute a major fraction of the tumor population, however their origin and role can vary vastly at different stages of cancer progression (244). Quiescent fibroblasts are reported to have anti-tumorigenic properties, however, it is believed that CAFs are the central architecture of tumorigenesis and are responsible for metabolic reprogramming *via* secretion of growth factors such as EGF, transforming growth factor 1 (TGF1), PGE2 and exosomes (242, 245). CAFs create a catabolic microenvironment that promotes tumor-initiating cells (CD133^+^/CXCR4^+^/EpCAM^+^) and induces stemness in cancer cells by activating sonic hedgehog and GLI signaling (246, 247). Gorchs et al. found that CAFs isolated from NSCLC tissue maintain their immunosuppressive effects *via* secretion of various immunomodulatory cytokines such as TGF-β, IL-6, and PGE2, even after high dose irradiation (248). Furthermore, CAFs can support the metabolic requirements of the tumor cells by providing various metabolites like lactate, amino acids and fatty acids (249, 250). Pavlides et al. proposed the reverse Warburg effect, where they observed that lactate produced by CAFs, due to upregulation of glycolytic enzymes, can be used by the cancer cells for respiratory metabolism (251). ROS generated by CRC cells stimulates lactate secretion by CAFs, and this lactate is utilized by CRC cells, which have high expression of lactate transporter MCT1 (252). Adipocytes in the cancer microenvironment regulate the switching between glucose and FA metabolism in cancer cells. Wen et al. reported that adipocytes transfer free fatty acids to the CRC cells and promote their survival by upregulating the AMPK pathway (250). Such metabolic coupling with the tumor-associated stroma promotes ATP generation and survival of cancer cells and can serve as a novel biomarker of cancer progression (253). In BC and GBM, a metabolic symbiosis also exists between tumor subpopulations in the oxygenated and hypoxic regions of the tumor, wherein hypoxic cells metabolise glucose to secrete lactate (through lactate transporter MCT4) which in turn is internalized by the oxygenated cells *via* MCT1 to produce energy through OXPHOS (254–259). This symbiosis permits the survival of heterogenic populations within a tumor while circumventing any competition over resources. Cancer progression of solid tumors requires the formation of new blood vessels to meet the oxygen and nutrient requirements of the tumor mass. Stromal cells secrete and induce the expression of proangiogenic factors VEGF and PGE2 in CRC cells (260). Additionally, CAFs can also modulate the extracellular matrix composition at tumor site to promote cancer cell motility (261).

The human gut microbiome also plays a significant role in reprogramming CRC metabolism. The gut microbiome expresses approximately 9.9 million genes, which is 150 times greater than the human transcriptome (262). CRC patients show alterations in the diversity of gut flora as harmful microbiome populations like- Fusobacterium and Prevotella increase, and populations of good bacteria like- butyrate-producing bacteria plummet (263).

### Immune cells

Along with CAFs and adipocytes, the metabolic signature of cancer cells is also influenced by surrounding immune cells, which include tumor-associated macrophages (TAM), tumor-infiltrating lymphocytes (TIL), and myeloid-derived suppressor cells (MDSC) (264). Macrophage infiltration is associated with poor prognosis in BC. Metabolic reprogramming in BC cells influences TAM differentiation, and increases glycolysis mediated by upregulation of hexokinase-2, PFK2 (ATP-dependent-6-phosphofructokinase), and enolase-1. These observations indicate that metabolically altered cancer cells can also reprogram the metabolism of associated macrophages to favour cancer progression (265).

Moreover, the accumulated lactate stabilizes the oxygen-regulated protein NDGR3, which can bind to c-Raf and promotes angiogenesis *via* the Raf-ERK signaling cascade (266). Lactate also activates breast cancer-associated macrophages and upregulates CCL5 and CCR5 expression by activating the NOTCH, TGFβ, and AMPK pathways, promoting EMT, migration, and aerobic glycolysis in BC cells through a positive feedback loop (267). BC cells induce the expression of hypoxia and stress response protein REDD1 in TAMs, which inhibits glycolysis *via* mTOR inhibition (268). Inhibition of glycolysis hinders the secretion of angiogenic factors by TAMs, leading to leaky, abnormal blood vessels that allow tumor metastasis (269). Coculture with TNBC cells induces the differentiation of monocytes into M2-like macrophages (M2-TAMs) and downregulates citrulline metabolism, nitric oxide synthase (iNOS), and nitric oxide (NO) (270, 271). The anti-inflammatory nature of M2-TAMs enables the cancer cells to evade immune recognition and promotes their survival. Increased glucose uptake and subsequent lactate accumulation by BC cells acidify the tumor microenvironment and impair the cytolytic activity of T-lymphocytes, thereby promoting tumorigenesis. The NK cell function is also hampered under high lactate conditions enabling cancer cells to evade immune surveillance (272, 273). MDSCs in the BC microenvironment promote immune evasion and upregulate glycolysis and phosphoenolpyruvate (PEP) accumulation, protecting BC cells from ROS-induced apoptosis (274). Furthermore, a higher number of MDSCs in the BC microenvironment is associated with increased metastasis (275).

Similarly, lung cancer progression is modulated by stromal and immune cells found in the lung microenvironment (276). Tumor-associated macrophages (TAMs) crosstalk with LC cells *via* the C-C chemokine receptor type-2 (CCR2) and CX3C chemokine receptor 1 (CX3CR1). LC cells also promote the conversion of TAMs into anti-inflammatory, tumor-supporting M2 phenotype, promoting cancer cell migration and survival (277). TAMs exhibit a glycolytic phenotype in the early stages of lung cancer, and tumor extract stimulates the expression of aerobic glycolysis and glycolytic enzymes HK2 and ENO1 in macrophages (265). The pro-tumoral TAMs secrete arginase II (ARG 2), which converts arginine to ornithine and urea and impairs T-cell response (278). TAMs also degrade tryptophan in the tumor microenvironment in lung cancer by upregulating indoleamine 2,3-dioxygenase (IDO) (279). Lack of arginine and tryptophan in the TME impairs T-cell response, whereas, the downstream metabolic product, Kynurenine, produced by tryptophan degradation increases the number of immunosuppressive regulatory T cells (Tregs) (280). Similarly, glutamine addiction of tumor cells deprives T cells of the glutamine supply, which is necessary for T-cell activation (281).

**Figure 6:**
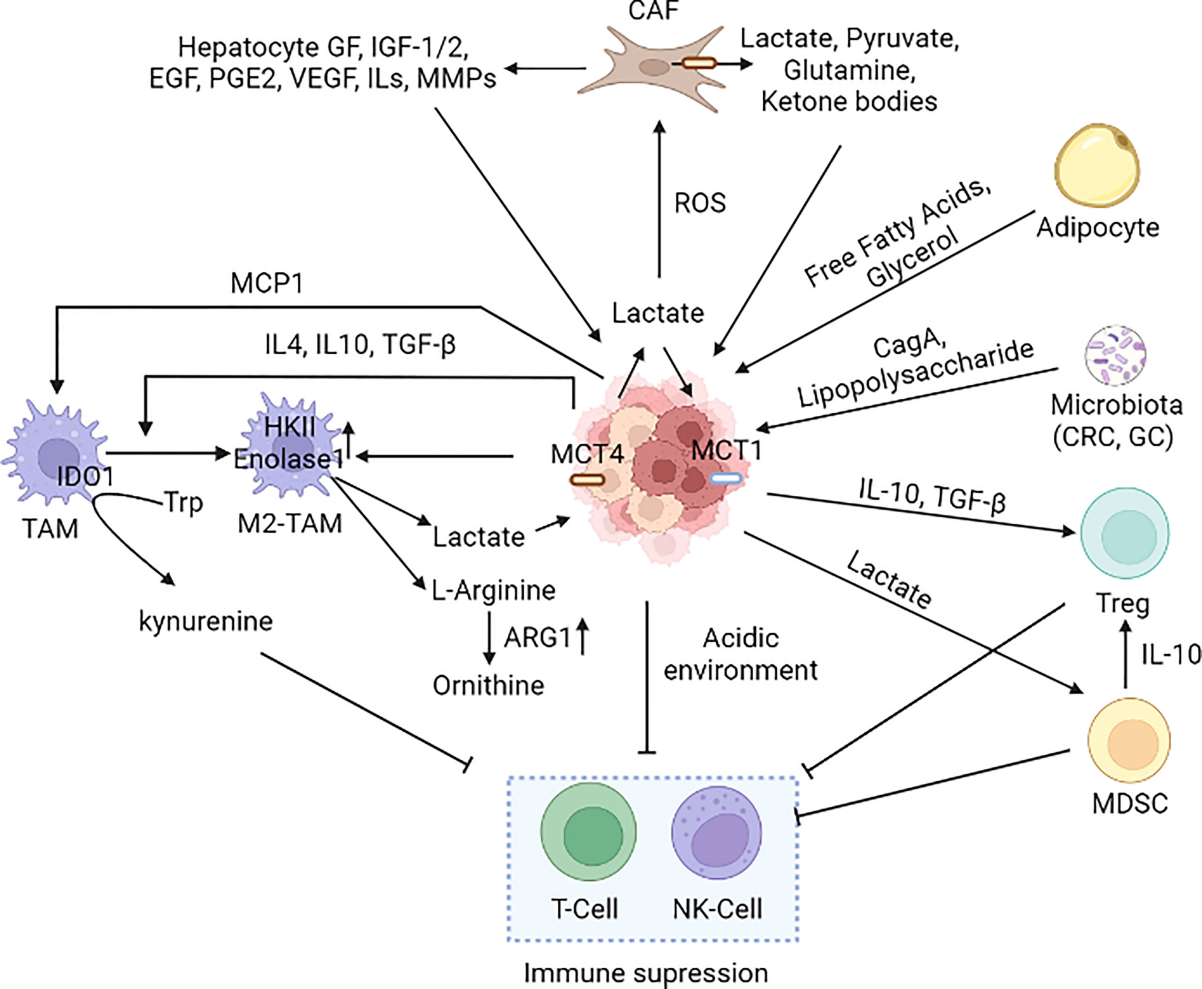
Cancer microenvironment in metabolic reprogramming. Several cell types such as macrophages, neutrophils, MDSCs, Treg, DC, T-cell, NK-cell, adipocytes, and CAFs present in the tumor microenvironment alter the metabolic reprogramming of cancer cells. Immune cells present in the TME have both pro and anti-tumor effects; however, the cancer cells induce the pro-tumorigenic phenotype (M2-TAM and M2-TAN). Cancer cells induce CAFs to secrete lactate in the TME leading to an acidic environment that suppresses the immune cells (242, 250, 252, 291–301). [MCT1: Monocarboxylate transporter 1, MCT4, Monocarboxylate transporter 4; CAF, Cancer-associated fibroblast; M2-TAM, M2 Type Tumor-associated macrophages; NK cell, Natural killer cell; MCP1/CCL2, Monocyte chemoattractant protein-1/Chemokine (C-C motif) ligand 2; IDO, Indoleamine-pyrrole 2,3-dioxygenase; MDSC, Myeloid-derived suppressor cells; Treg, T regulatory cells; IGF, Insulin-like Growth Factor; EGF, Epidermal growth factor; VEGF, Vascular endothelial growth factor; IL-Interleukin, PGE2-Prostaglandin E2; TGF-β, Transforming growth factor beta, MMP: Matrix metalloproteases, CRC, Colorectal cancer; GC, Gastric cancer].

M2 polarization of TAMs is also observed in HCC, inducing an immunosuppressive microenvironment (282). M2 macrophages secrete IL-1β, which induces FAO and increases the proliferation and metastatic potential of HCC cells. M2-like TAMs in HCC-TME also show a reduction in glycolysis and PPP with enhanced OXPHOS and FAO (283). Metabolic modifications in tumor-associated neutrophils (TANs) also play a key role in tumor growth, invasion, and metastasis. Neutrophils derive energy from glycolysis to maintain their function at low oxygen levels (284, 285). However, lack of glucose in the TME obligates neutrophils to utilize mitochondrial FAO and produce NOX-2-dependent ROS, which further increases immune tolerance in the TME (286). Like TANs, the dependence of regulatory T cells on FAO for their survival in TME also plays an immunosuppressive role (287, 288). Of note, the glycolytic nature of the TME inhibits the functions of effector T-cells, but maintains the proliferation and function of regulatory T cells (289, 290). Thus, tumor-associated stromal cells and immune cells play a crucial role in tumor progression in several cancers, and effective strategies should be derived to inhibit the metabolic crosstalk between the tumor cells and microenvironment cells (**Figure 6**).

## Metabolic targets for cancer therapy

Metabolic targeting of cancer is a new era of precision therapy that aims to exploit the dependence of malignancies on specific metabolic alterations. The excess glycolysis, the first described metabolic dysregulation in cancers, has been targeted in multiple ways to induce growth arrest and apoptosis in tumor cells. Using 2-deoxyglucose (2-DG) was one of the earliest attempts to inhibit glycolysis in cancer cells. 2-DG is transported by hexose transporters and converted to 2-DG-6-phosphate by hexokinase, which cannot be metabolised further, and its accumulation impedes glycolysis by inhibiting hexokinase and phospho-glucose isomerase. However, 2-DG did not show clinical benefits due to its low therapeutic index (302–304), and 2-DG inhibits the necessary high glucose metabolism in the brain (305). However, studies have shown that the biological effects of 2-DG are not solely due to its metabolic block; therefore, targeting glucose metabolism *via* other means might still have therapeutic benefits (306). For instance, other glycolysis inhibitors such as 3-bromopyruvate (3-BP) (307) and lonidamine (308) are being explored to treat cancers with minimal side-effects like alopecia and bone-marrow suppression observed with conventional chemotherapies (302, 309, 310). Phosphofructokinases and their regulatory gene PFKFB are also dysregulated in transformed cells and primary cancers (311). Upregulation of PFKFB3 promotes glycolysis, helps in cell cycle transition by phosphorylating p27, and is associated with poor overall survival in BC patients making it a potential target for treatment (312, 313). 3-(3-pyridinyl)-1-(4-pyridinyl)-2-propen-1-one (3-PO) is a PFKFB3 inhibitor that interacts with its functional subunit and depletes fructose-2,6-bisphosphate, but has limited application due to its water insolubility. However, PFK15 and PFH158, the functional derivatives of 3-PO, are tested in the clinical trial as PFKFB inhibitors (314, 315). Furthermore, inhibition of glucose uptake by targeting upregulated GLUTs using WZB117, silibinin or cytochalasin B induces metabolic crisis in breast, lung, and colorectal cancer cells (238, 316, 317). Similarly, AR-C155858 and AZD3965, inhibitors of lactate transporters MCT1 and 2, normalise the TME pH and inhibit tumor proliferation (318, 319). LDH upregulation has also been targeted with quinoline-3-sulfonamide, Oxamate and GNE-140 to inhibit conversion of pyruvate into lactate. Specific targeting of mutant enzymes such as IDH1 and IDH2, using enasidenib (AG-221) or ivosidenib (AG-88), is another approach to target glucose metabolism without affecting the function of normal cells. However, the relevance of such mutations to tumor survival and proliferation largely depends on the cancer stage and might not be an effective strategy during later stages.

OXPHOS is generally downregulated in cancer cells (breast cancer, gastric cancer), but due to mutations in mitochondrial DNA, OXPHOS is upregulated in some cancer types (leukaemia, endometrial carcinoma) (38). Targeting OXPHOS requires inhibition of the ETC complexes. Metformin inhibits ETC complex I and reduces tumor growth by reducing NADH oxidation, resulting in ATP depletion and loss of proton gradient across the inner mitochondrial membrane (320, 321). In addition to metformin, drugs such as tamoxifen, Vitamin E derivative α-tocopheryl succinate, and 3-BP target ETC complexes (322). On the other hand, the independence of cancer cells from mitochondrial respiration mediates their resistance to the mitochondria-controlled apoptotic pathway. PDK regulates the activity of PDH to limit the production of acetyl-CoA from pyruvate and is a potential target for cancer therapy. Dichloroacetate inhibits PDK, upregulates the activity of PDH, and restores the mitochondrial dependence of cancer cells (323).

Downregulation of PPP to inhibit nucleotide biosynthesis is another therapeutic approach to limit the proliferation of cancer cells. Dehydroepiandrosterone, an endogenous precursor for steroid hormones, can inhibit G6PD, the key regulator of PPP, but has limited therapeutic application due to its immediate conversion into steroid hormones *in vivo* (324). 6-aminonicotinamide is reported to inhibit G6PD and showed promising results in inhibiting tumor growth *in vitro* (325–327), but its inclusion within tolerable doses in subsequent clinical trials showed no significant benefit (328).

Glutamine addiction is another prominent characteristic of cancer cells that can be targeted therapeutically. Glutaminase1 (GLS1) inhibitor, Bis-2-(5-phenylacetamido-1,3,4-thiadazol-2-yl) ethyl sulphide reduces cancer cell proliferation *in vivo* (329–331). Several other molecules, such as acivicin, azaserine, 6-diazo-5-oxo-I-norleucine (DON), which can inhibit glutaminolysis, can be used for cancer treatment (329, 332). L-asparaginase, which depletes asparagine and glutamine in the microenvironment of ALL cells to inhibit their growth, have been found to be effective in several patients (333). Serine, a precursor for nonessential amino acids glycine and cysteine, is synthesised from glucose or imported from the extracellular environment by the cancer cells to promote their survival (334–336). Upregulation of phosphoglycerate dehydrogenase (PHGD), which converts 3-phosphoglycerate to serine, can be targeted using PHGD inhibitors CBR-588 and NCT-503 in BC and LC (305).

Many cancer types, such as glioblastoma, are dependent on lipid metabolism. In nutrient-deprived conditions, cancer cells use FAO to produce NADH and FADH2 *via* OXPHOS (337). During cancer progression, upregulation of CPT1 increases the transport of fatty acids into the mitochondria. CPT1A inhibitor, etomoxir (ETO), induces growth arrest in bladder cancer cells (338) and increases the effectiveness of hormonal therapeutic drugs like enzalutamide in prostate cancer (339). Further studies identified that etomoxir has off target effects which leads to oxidative stress, therefore, using other CPT1A inhibitor (S1326) might yield better therapeutic results (340, 341). Furthermore, FAO is upregulated due to the increased activity of enzymes like ACLY, ACC1 and ACC2 that are crucial for cancer cell growth and proliferation, making them a potential target for GC, LC, and BC treatment (342). Drugs like SB-204990 and simvastatin inhibit ACLY and decrease acetyl-CoA availability for FAO (82, 343–345). FASN is overexpressed in most cancer types to promote lipogenesis (154, 346).

Like other metabolic pathways nucleotide metabolism is also dysregulated in cancer. Targeting nucleotide metabolic enzymes or using purine and pyrimidine analogs has been long sought therapeutic approach for cancer treatment. Thiopurines (6-mercaptopurine, thioguanine), deoxy-purines (cladribine, Clofarabine), arabinose purine analogs (nelarabine, fludarabine) and base modified purine nucleotides (8-chloro-adenosine, tocladesine and forodesine) are purine analog antimetabolites which are used for cancer treatment (347). 6-mercaptopurine was approved by FDA for the treatment of childhood leukaemia in early 1953 and thioguanine in 1996 for the treatment of non-lymphocytic leukaemia (347–350). Cladribine is approved for the treatment of hairy cell leukaemia and its improved version is clofarabine a second-generation deoxyadenosine analog (351–354). Arabinose purine analogs are used for the treatment of acute and chronic lymphocytic leukaemia and in relapsed T cell lymphoblastic leukaemia, and base modified purine nucleotides have not yet approved by FDA (351–357). Fluorinated pyrimidines (5-Fluro uracil, capecitabine, floxuridine), azanucleosides (decitabine, azacytidine), ribose sugar modified cytidine analogs (gemcitabine), cytarabine are used as a pyrimidine analogue for treatment of cancer (347). 5-FU was approved by FDA in 1960 for hepatic carcinogenesis and is currently under study for gastrointestinal, breast and renal cancer (358–360). Gemcitabine, a ribose sugar modified cytidine analog, disrupts DNA biosynthesis through cell cycle arrest and is currently approved by FDA for the treatment of breast, ovarian, lung and pancreatic cancer (361–363). Azanucleosides induce epigenetic modification (inhibit DNA methylation) to achieve antitumor effects (364–366). Along with the purine and pyrimidine nucleotide analogs there are enzymatic blockers which can disrupt nucleotide metabolism in cancer cells. Mizoribine, merimepodib and mycophenolic mofetil (phase I trial in pancreatic cancer) are inhibitors of IMDPH an enzyme involved in purine metabolism (367–370). Thymidylate synthase (TS) and glycinamide ribonucleotide transformylase (GART) which are involved in thymidine and purine nucleotide synthesis is inhibited by an anti-folate drug called pemetrexed. MLN4924 can be used in melanoma, acute myeloid leukemia and lymphoma, as it inhibits carcinogenesis by inhibiting proteasomal degradation (371, 372). Teriflunomide and leflunomide, inhibitor of DHODH a key enzyme in nucleotide synthesis, have antiproliferative effects in NSCLC, myeloma and neuroblastoma but has not yet been approved by FDA for clinical use (373, 374). TVB-3166, TVB-2640, and omeprazole inhibit the FASN to inhibit cancer proliferation. Several signaling pathways (PI3K/AKT/mTORC, MAPK, PI3K/AKT/FOXO) are also involved in the metabolic alteration of cancer cells, which can be targeted to downregulate the metabolic pathways and ultimately inhibit cancer growth and proliferation.

Dysregulation of GLUT-1/3, HK II, PFK-1 CPT1/2, FASN and GLS1 is common in most cancer types, and the interlinked metabolic pathways compensate for the deprivation of nutrients to maintain cancer growth, proliferation, and metastasis. Therefore, combination therapies targeting more than one metabolic pathway will be more effective with a better therapeutic index to treat cancer. Kalyanaraman et al. reported that targeting both glycolysis and OXPHOS is a promising therapeutic approach and observed good outcomes with a combination of Metformin and 2-DG treatment in pancreatic cancer which depleted the ATP pool and cancer proliferation (375). L-asparaginase can be combined with other glutamine metabolism inhibitors, which can increase the effectiveness of the treatment. Tanaka et al. observed that a combination treatment of mTOR and GLS inhibitors induced tumor cell death in a mice model (376).

Along with metabolic and signaling pathways, targeting TME cells can be a potential therapeutic option by which we can produce a synergistic effect against the cancer cells. CAF present in the microenvironment undergo aerobic glycolysis and produce lactate which is released into the microenvironment by MCT4 and imported by the cancer cells *via* MCT1 to fuel up the OXPHOS during nutrient deprived conditions (377, 378). Syrosingopine, which can inhibit both MCT1 and MCT4 and LDH has been shown to induce higher cytotoxicity than AZD3965 which can only inhibit MCT-1 in liver cancer cells (377). CAFs are also a source of glutamine in the TME so targeting glutamine synthetase (GLUL) in CAFs along with GS in cancer cells leads to synergistic effect which reduces metastatic potential of ovarian cancer (379). Endothelial cells are reprogrammed to undergo excessive glycolysis in the TME leading to their impaired function, so targeting glycolytic activator PFKFB3 with 3-PO, can be a multimodal therapeutic option for cancer treatment (380, 381).

However, caution needs to be exercised as inhibiting certain metabolic pathways can severely impair the immune cell function, which in turn might promote tumor progression. Furthermore, tumor microenvironment components are also being explored as a viable target to disrupt the metabolic cooperation that promotes tumor progression (17) (**Table 1**).

**Table 1:** List of metabolic pathway drugs which were evaluated clinically.

Drug	Target molecule	Mode of action in different cancer types
AZD3965 AR-C155858	MCT1	Inhibition of lactate transporter and glycolysis in CRC (235) and LC (236).
WZB117 Silibinin Cytochalasin B Fasentin, phloretin, STF-3, Dapagliflozin	GLUT SGLT	Downregulates GLUT1 expression in CRC (235), Inhibits glucose uptake in cells preferentially expressing GLUT1 or GLUT4 and inhibits active transport of glucose into cells by SGLT1 and SGLT2 in LC (382–385), Inhibit glucose reabsorption in the kidney in BC (386)
2-DG Lonidamine Bromopyruvate	HK	Sensitizes CRC cells to TRAIL-induced apoptosis (310). *Inhibits* glycolysis in hypoxic cancer cells and inhibits mitochondrial pyruvate oxidation in BC (302) and LC (302, 309, 387).
Metformin, Mito-Metformin	Mitochondrial electron transporter complex 1	AMPK activation, PKM2 inhibition, and ATP depletion in CRC (388, 389) Inactivates phosphorylation of the E1 alpha-subunit of PDC and inhibits alpha-ketoglutarate dehydrogenase in BC (390).
Dichloroacetate AZD7545	PDK	Fostering oxidative phosphorylation in BC (391). Increases oxidative phosphorylation, thereby increasing Krebs cycle intermediate concentration in LC (392, 393)
CPI-613 ME-344	PDH	Inactivate phosphorylation of the E1 alpha-subunit of PDC; and inhibits alpha-ketoglutarate dehydrogenase and decreases mitochondrial ATP production in BC (394, 395).
Indoximod, Epacadostat, BMS-986205, KHK2455, Epacadostat, INCB001158	Indoleamine 2,3 dioxygenase (IDO1)	Increases tryptophan concentration and activates T cell-mediated immunity In BC (106, 396). Induces host immune system mainly T-cell mediated response in BLC (397, 398).
3PO PFK3 PFK158	PFKFB3	Reduces glucose uptake and ATP production in LC (399, 400).
Bromop-yruvate	GAPDH	Inhibit glycolysis, thereby suppressing the production of ATP and inducing cell death in LC (401, 402).
Shikonin	PKM2	Inhibits cell aerobic glycolysis in LC (403).
FX11 Quinoline-3-sulfonamide Oxamate GNE-140 PSTMB	LDH	Inhibits the ability of LDH to convert pyruvate into lactate in LC (237, 404–407).
Enasidenib Ivosedinib GSK864 GSK321	IDH	Potent IDH1 inhibitor used in LC and approved in relapsed/refractory IDH2 mutant AML (408, 409).
Lonidamine SiRNA	VDAC1	Induces apoptosis by rewiring tumor cell metabolism, reduces cancer stem cells, and induces differentiation in LC (410, 411).
Simvastatin	3-hydroxy-3-methylglutaryl coenzyme A reductase	Inhibit conversion of HMG-CoA to mevalonate, the rate limiting step of steroidogenesis in PC (412).
INCB001158	Arginase	Induces proliferation of cytotoxic T-cells and natural killer (NK) cells that kill the tumor cells In BLC (413).
L-NMMA	Nitric oxide synthase	Inhibits NOS in BLC (397).
ADI-PEG20	Arginine deiminase	Starves tumor cells by *arginine* depletion in BC (414).
CB-839	Glutaminase	Inhibits hydrolysis of glutamine in BC (415)
DON, BPTES, Acivicin, Azaserine	GLS1	Inhibits Gln metabolism in CRC (416, 417) and reduces glucose uptake in PC (418).
L-aspartate + Rapamycin	ASNs, mTOR	Inhibits asparagine biosynthesis in CRC (112)
TVB-3166, TVB-2640, Omeprazole, Conjugated Linoleic Acid (CLA), Orlistat, C75, cerulenin, and C93, 3-aryl-4 hydroxyquinoline-2(1H)-one derivatives, GlaxoSmithKline produced GSK837149	FASN	Inhibits de novo palmitate synthesis in CRC (343). Supresses denovo lipogenesis in BC (82, 344, 345). Form a covalent bond with the enzyme FASN and inhibits its function, thereby inhibiting lipogenesis in PC (419–421).
SB-204990 and simvastatin	ATP citrate lyase	RNA interference and inhibits proliferation and survival of tumor cells in PC (422).
Ficlatuzumab	FGF	Inhibits glucose uptake and blocks HGF activity in CRC (106).
RO5126766	RAF/MEK	Downregulates GLUT1 expression in CRC (133).
Metformin, 5-aminoimidazole-4-carboxamide-1-b-ribofuranoside (AICAR), A-769662, PT1, and OSU-53	AMPK	Reduces the expression of gluconeogenic enzymes, decreases AMP/ATP ratio, and activates AMPK in PC (421, 423–425).
Mercaptopurine	Hypoxanthine–guanine phosphoribosyltransferase, amidophosphoribosyltransferase, Inosine-5'-monophosphate dehydrogenase	Acute lymphatic leukemia (347, 348)
Fluorouracil	Thymidylate synthase	Colon, esophageal, gastric, rectum, breast, biliary tract, stomach, head and neck, cervical, pancreas and renal cell cancer (302, 359)
Thioguanine Carboplatin Oxaliplatin	DNA	Acute non-lymphocytic leukemias (347, 350) Testicular tumors, ovarian tumors, and bladder cancer CRC (426)
Gemcitabine	Ribonucleoside-diphosphate reductase, thymidylate synthase, UMP-CMP kinase	Ovarian, lung, breast, and pancreas cancer (361, 362)
Pemetrexed	Thymidylate synthase, Bifunctional purine biosynthesis protein PURH, Dihydrofolate reductase, Trifunctional purine biosynthetic protein adenosine 3	Mesothelioma, NSCLC (427)

## Conclusion

Metabolic dysregulations in cancer cells were first described by Otto Warburg, who identified and hypothesised that excessive glucose uptake and lactate formation is the root cause of tumorigenesis (6). Since then, numerous studies have identified alterations in all major metabolic pathways, including glucose, amino acids, lipid, and nucleotide metabolism.

The current understanding of tumor progression suggests that while metabolic reprogramming does not lead to tumor initiation, it is the most critical driver of tumor progression in later stages. But in some cases, mutation in metabolic enzymes leads to tumorigenesis. Oncometabolites (succinate, fumarate, D-2-hydroxyglutarate) produced from mutations in TCA cycle enzymes succinate dehydrogenase (SDH), fumarate hydratase (FH) and IDH1/2 lead to epigenetic alterations and affect gene expression by inhibition of Jumanji-C-domain containing histone lysine demethylase (KDMs) and ten-eleven translocation (TET) family of 5-methylcytosine (5mC) hydroxylase (428–430). These oncometabolites are observed across different cancer types such as renal cancer, gastrointestinal cancers, leukaemia gliomas and glioblastomas (430). In some cases, risk factors like obesity, may also lead to tumorigenesis, mediated by increased leptin and decreased adiponectin. Leptin leads to the activation of PI3K-AKT-mTOR pathway, whereas downregulation of adiponectin reduces AMPK levels, thereby increasing mTORC1 activity, which induces cell proliferation (431, 432).

Induced glucose fermentation, also referred to as “aerobic glycolysis” or the Warburg effect, provides for the high energy requirements of the cancer cells. The upregulation of glucose consumption forms the basis of tumor screening by fluorodeoxyglucose positron emission tomography (FDG-PET) (433). This increase in glycolytic flux is due to the upregulation of various glucose importers (GLUTs) and downstream metabolising enzymes like HK, PFK, PKM2, and LDH, observed in many cancer types (39, 55, 433, 434). Furthermore, high glycolytic flux is coupled with upregulation of PPP, increasing nucleic acid biosynthesis required for unabated proliferation of cancer cells (59). Cancer cells derive energy *via* oxidative phosphorylation of TCA cycle intermediates produced by amino acid metabolism or *via* beta-oxidation of fatty acids (98, 150, 151). Increased metabolism of glutamine and auxotrophic dependence on other amino acids is a prominent characteristic of cancer cells. The exclusive sensitivity of ALL cells towards L-asparaginase treatment was the first therapeutic intervention that targeted metabolic dysregulation in tumor cells. Since then, various other approaches that limit the availability of amino acids, such as treatment with arginase, inhibition of AA metabolism, such as glutaminase1 inhibitors and inhibition of amino acid transporters like ASCT, have shown promising results in inhibiting tumor growth. Glutaminase inhibitors, IPN60090, a small molecule, and Telaglenastat are in phase 1 clinical trial for CRC, NSCLC and PC (435, 436). There are also other drugs available which are in clinical trial for targeting different cancer types such as- sirpiglenastat an antagonist of glutamine (phase I/II for solid tumors and NSCLC), AZD5965 inhibitor of MCT1 (phase II for solid tumors and lymphoma), IACS-010759 inhibitor of mitochondrial respiratory complex 1 (phase I for AML) (436). Similarly, various aspects of fatty acid metabolism and lipogenesis upregulated in cancer cells are targeted by inhibiting the regulatory enzymes such as FASN. In glioblastoma, cancer cells do not depend on extrinsic sources of fatty acid and instead upregulate *de novo* synthesis of cholesterol. Under such circumstances targeting specific regulators of lipogenesis such as SREBP1 shows high therapeutic specificity and efficacy (167, 169).

Furthermore, the tumor microenvironment comprises various immune cells and tumor-associated stromal cells, which extensively modulate the metabolic state of the cancer cells. The symbiotic relationship between cancer and TME cells supports the metabolic dysregulations resulting in a tumorigenic and metastasis-promoting niche. Various metabolites secreted by cancer cells induce tumor suppressive phenotype in the macrophages and regulatory T-cells. Furthermore, cooperative metabolic coupling within different subpopulations of tumor cells are also observed. Secretion of various metabolites by the TME cells induces signaling pathways such as AMPK/mTORC1, PI3K/AKT and Raf-ERK, which promote the survival of cancer cells.

Therefore, further studies aimed at a better sub-classification of cancers depending on the type of interactions that exist between the cancer cells and its TME might allow targeting of TME-induced metabolic alterations.

## Author contributions

SP, AS, SM and BJ wrote the manuscript and approved the final version of the manuscript. All authors contributed to the article and approved the submitted version.

## References

[1] Sung H Ferlay J Siegel RL Laversanne M Soerjomataram I Jemal A. Global cancer statistics 2020: GLOBOCAN estimates of incidence and mortality worldwide for 36 cancers in 185 countries. CA Cancer J Clin (2021) 71(3):209–49. doi: 10.3322/caac.21660 33538338

[2] Hanahan D Weinberg RA. The hallmarks of cancer. Cell (2000) 100(1):57–70. doi: 10.1016/s0092-8674(00)81683-9 10647931

[3] Faubert B Solmonson A DeBerardinis RJ. Metabolic reprogramming and cancer progression. (2020) 368(6487):eaaw5473. doi: 10.1126/science.aaw5473 PMC7227780 32273439

[4] Hanahan D Weinberg RA. Hallmarks of cancer: the next generation. Cell (2011) 144(5):646–74. doi: 10.1016/j.cell.2011.02.013 21376230

[5] Fernie AR Carrari F Sweetlove LJ. Respiratory metabolism: glycolysis, the TCA cycle and mitochondrial electron transport. Curr Opin Plant Biol (2004) 7(3):254–61. doi: 10.1016/j.pbi.2004.03.007 15134745

[6] Liberti MV Locasale JW. The warburg effect: How does it benefit cancer cells? Trends Biochem Sci (2016) 41(3):211–8. doi: 10.1016/j.tibs.2015.12.001 PMC4783224 26778478

[7] DeBerardinis RJ Mancuso A Daikhin E Nissim I Yudkoff M Wehrli S. Beyond aerobic glycolysis: transformed cells can engage in glutamine metabolism that exceeds the requirement for protein and nucleotide synthesis. Proc Natl Acad Sci U.S.A. (2007) 104(49):19345–50. doi: 10.1073/pnas.0709747104 PMC2148292 18032601

[8] DeBerardinis RJ Chandel NS. Fundamentals of cancer metabolism. Sci Adv (2016) 2(5):e1600200. doi: 10.1126/sciadv.1600200 27386546 PMC4928883

[9] Läsche M Emons G Gründker C. Shedding new light on cancer metabolism: A metabolic tightrope between life and death. Front Oncol (2020) 10:409. doi: 10.3389/fonc.2020.00409 32300553 PMC7145406

[10] Pavlova NN Thompson CB. The emerging hallmarks of cancer metabolism. Cell Metab (2016) 23(1):27–47. doi: 10.1016/j.cmet.2015.12.006 26771115 PMC4715268

[11] Kaelin WG Jr. McKnight SL. Influence of metabolism on epigenetics and disease. Cell (2013) 153(1):56–69. doi: 10.1016/j.cell.2013.03.004 23540690 PMC3775362

[12] Tomlinson IP Alam NA Rowan AJ Barclay E Jaeger EE Kelsell D. Germline mutations in FH predispose to dominantly inherited uterine fibroids, skin leiomyomata and papillary renal cell cancer. Nat Genet (2002) 30(4):406–10. doi: 10.1038/ng849 11865300

[13] Baysal BE Ferrell RE Willett-Brozick JE Lawrence EC Myssiorek D Bosch A. Mutations in SDHD, a mitochondrial complex II gene, in hereditary paraganglioma. Science (2000) 287(5454):848–51. doi: 10.1126/science.287.5454.848 10657297

[14] Liou GY Storz P. Reactive oxygen species in cancer. Free Radic Res (2010) 44(5):479–96. doi: 10.3109/10715761003667554 PMC3880197 20370557

[15] Srinivas US Tan BWQ Vellayappan BA Jeyasekharan AD. ROS and the DNA damage response in cancer. Redox Biol (2019) 25:101084. doi: 10.1016/j.redox.2018.101084 30612957 PMC6859528

[16] Yuneva MO Fan TW Allen TD Higashi RM Ferraris DV Tsukamoto T. The metabolic profile of tumors depends on both the responsible genetic lesion and tissue type. Cell Metab (2012) 15(2):157–70. doi: 10.1016/j.cmet.2011.12.015 PMC3282107 22326218

[17] Gupta S Roy A Dwarakanath BS. Metabolic cooperation and competition in the tumor microenvironment: Implications for therapy. Front Oncol (2017) 7:68. doi: 10.3389/fonc.2017.00068 28447025 PMC5388702

[18] Kalyanaraman B. Teaching the basics of cancer metabolism: Developing antitumor strategies by exploiting the differences between normal and cancer cell metabolism. Redox Biol (2017) 12:833–42. doi: 10.1016/j.redox.2017.04.018 PMC5406543 28448945

[19] Agnihotri S Zadeh G. Metabolic reprogramming in glioblastoma: the influence of cancer metabolism on epigenetics and unanswered questions. Neuro Oncol (2016) 18(2):160–72. doi: 10.1093/neuonc/nov125 PMC4724176 26180081

[20] Burns JE Hurst CD Knowles MA Phillips RM Allison SJ. The warburg effect as a therapeutic target for bladder cancers and intratumoral heterogeneity in associated molecular targets. Cancer Sci (2021) 112(9):3822–34. doi: 10.1111/cas.15047 PMC8409428 34181805

[21] Chen H Wu Q Peng L Cao T Deng M-L Liu Y-W. Mechanism, clinical significance, and treatment strategy of warburg effect in hepatocellular carcinoma. J Nanomaterials (2021) 10. doi: 10.1155/2021/5164100

[22] Fang S Fang X. Advances in glucose metabolism research in colorectal cancer. BioMed Rep (2016) 5(3):289–95. doi: 10.3892/br.2016.719 PMC4998148 27602209

[23] Kalezic A Udicki M Srdic Galic B Aleksic M Korac A Jankovic A. Tissue-specific warburg effect in breast cancer and cancer-associated adipose tissue-relationship between AMPK and glycolysis. Cancers (Basel) (2021) 13(11):2731. doi: 10.3390/cancers13112731 34073074 PMC8198826

[24] Vanhove K Graulus GJ Mesotten L Thomeer M Derveaux E Noben JP. The metabolic landscape of lung cancer: New insights in a disturbed glucose metabolism. Front Oncol (2019) 9:1215. doi: 10.3389/fonc.2019.01215 31803611 PMC6873590

[25] Yuan LW Yamashita H Seto Y. Glucose metabolism in gastric cancer: The cutting-edge. World J Gastroenterol (2016) 22(6):2046–59. doi: 10.3748/wjg.v22.i6.2046 PMC4726677 26877609

[26] Pértega-Gomes N Vizcaíno JR Attig J Jurmeister S Lopes C Baltazar F. A lactate shuttle system between tumour and stromal cells is associated with poor prognosis in prostate cancer. BMC Cancer (2014) 14:352. doi: 10.1186/1471-2407-14-352 24886074 PMC4039335

[27] Schöder H Larson SM. Positron emission tomography for prostate, bladder, and renal cancer. Semin Nucl Med (2004) 34(4):274–92. doi: 10.1053/j.semnuclmed.2004.06.004 15493005

[28] Testa C Pultrone C Manners DN Schiavina R Lodi R. Metabolic imaging in prostate cancer: Where we are. Front Oncol (2016) 6:225. doi: 10.3389/fonc.2016.00225 27882307 PMC5101200

[29] Ghanbari Movahed Z Rastegari-Pouyani M Mohammadi Mh Mansouri K. Cancer cells change their glucose metabolism to overcome increased ROS: One step from cancer cell to cancer stem cell? BioMed Pharmacother (2019) 112:108690. doi: 10.1016/j.biopha.2019.108690 30798124

[30] Zhuang X Chen Y Wu Z Xu Q Chen M Shao M. Mitochondrial miR-181a-5p promotes glucose metabolism reprogramming in liver cancer by regulating the electron transport chain. Carcinogenesis (2020) 41(7):972–83. doi: 10.1093/carcin/bgz174 31628462

[31] Chen CL Uthaya Kumar DB Punj V Xu J Sher L Tahara SM. NANOG metabolically reprograms tumor-initiating stem-like cells through tumorigenic changes in oxidative phosphorylation and fatty acid metabolism. Cell Metab (2016) 23(1):206–19. doi: 10.1016/j.cmet.2015.12.004 PMC4715587 26724859

[32] Soukupova J Malfettone A Hyroššová P Hernández-Alvarez MI Peñuelas-Haro I Bertran E. Role of the transforming growth factor-β in regulating hepatocellular carcinoma oxidative metabolism. Sci Rep (2017) 7(1):12486. doi: 10.1038/s41598-017-12837-y 28970582 PMC5624948

[33] Wu W Zheng X Wang J Yang T Dai W Song S. O-GlcNAcylation on Rab3A attenuates its effects on mitochondrial oxidative phosphorylation and metastasis in hepatocellular carcinoma. Cell Death Dis (2018) 9(10):970. doi: 10.1038/s41419-018-0961-7 30237463 PMC6148238

[34] LeBleu VS O'Connell JT Gonzalez Herrera KN Wikman H Pantel K Haigis MC. PGC-1α mediates mitochondrial biogenesis and oxidative phosphorylation in cancer cells to promote metastasis. Nat Cell Biol (2014) 16(10):992–1003. doi: 10.1038/ncb3039 25241037 PMC4369153

[35] D'Errico I Salvatore L Murzilli S Lo Sasso G Latorre D Martelli N. Peroxisome proliferator-activated receptor-gamma coactivator 1-alpha (PGC1alpha) is a metabolic regulator of intestinal epithelial cell fate. (2011) 108(16):6603–8. doi: 10.1073/pnas.1016354108 PMC3081029 21467224

[36] Bellafante E Morgano A Salvatore L Murzilli S Di Tullio G D'Orazio A. PGC-1β promotes enterocyte lifespan and tumorigenesis in the intestine. Proc Natl Acad Sci U.S.A. (2014) 111(42):E4523–31. doi: 10.1073/pnas.1415279111 PMC4210309 25288742

[37] Reznik E Miller ML Şenbabaoğlu Y Riaz N Sarungbam J Tickoo SK. Mitochondrial DNA copy number variation across human cancers. Elife (2016) 5:1207. doi: 10.7554/eLife.10769 PMC4775221 26901439

[38] Yu M. Generation, function and diagnostic value of mitochondrial DNA copy number alterations in human cancers. Life Sci (2011) 89(3-4):65–71. doi: 10.1016/j.lfs.2011.05.010 21683715

[39] Li XB Gu JD Zhou QH. Review of aerobic glycolysis and its key enzymes - new targets for lung cancer therapy. Thorac Cancer (2015) 6(1):17–24. doi: 10.1111/1759-7714.12148 26273330 PMC4448463

[40] Sonveaux P Vegran F Dewhirst M Feron O. Targeting lactate exchanges in tumours: From basic characterization to new therapeutic applications. Radiother Oncol (2010) 96:S57–S.

[41] Schell JC Olson KA Jiang L Hawkins AJ Van Vranken JG Xie J. A role for the mitochondrial pyruvate carrier as a repressor of the warburg effect and colon cancer cell growth. Mol Cell (2014) 56(3):400–13. doi: 10.1016/j.molcel.2014.09.026 PMC4268416 25458841

[42] Choudhary D Hegde P Voznesensky O Choudhary S Kopsiaftis S Claffey KP. Increased expression of l-selectin (CD62L) in high-grade urothelial carcinoma: A potential marker for metastatic disease. Urol Oncol (2015) 33(9):387.e17–27. doi: 10.1016/j.urolonc.2014.12.009 PMC4510033 25618296

[43] Pate KT Stringari C Sprowl-Tanio S Wang K TeSlaa T Hoverter NP. Wnt signaling directs a metabolic program of glycolysis and angiogenesis in colon cancer. EMBO J (2014) 33(13):1454–73. doi: 10.15252/embj.201488598 PMC4194089 24825347

[44] Woolbright BL Choudhary D Mikhalyuk A Trammel C Shanmugam S Abbott E. The role of pyruvate dehydrogenase kinase-4 (PDK4) in bladder cancer and chemoresistance. Mol Cancer Ther (2018) 17(9):2004–12. doi: 10.1158/1535-7163.Mct-18-0063 PMC6724734 29907593

[45] Le A Lane AN Hamaker M Bose S Gouw A Barbi J. Glucose-independent glutamine metabolism *via* TCA cycling for proliferation and survival in b cells. Cell Metab (2012) 15(1):110–21. doi: 10.1016/j.cmet.2011.12.009 PMC3345194 22225880

[46] Lane AN Fan TW Bousamra M 2nd Higashi RM Yan J Miller DM. Stable isotope-resolved metabolomics (SIRM) Cancer Res Clin Appl to nonsmall Cell Lung cancer. Omics (2011) 15(3):173–82. doi: 10.1089/omi.2010.0088 PMC3125551 21329461

[47] Yang C Wang S Ruan H Li B Cheng Z He J. Downregulation of PDK4 increases lipogenesis and associates with poor prognosis in hepatocellular carcinoma. J Cancer (2019) 10(4):918–26. doi: 10.7150/jca.27226 PMC6400816 30854098

[48] Sellers K Fox MP Bousamra M 2nd Slone SP Higashi RM Miller DM. Pyruvate carboxylase is critical for non-small-cell lung cancer proliferation. J Clin Invest (2015) 125(2):687–98. doi: 10.1172/jci72873 PMC4319441 25607840

[49] Costello LC Franklin RB. The clinical relevance of the metabolism of prostate cancer; zinc and tumor suppression: connecting the dots. Mol Cancer (2006) 5:17. doi: 10.1186/1476-4598-5-17 16700911 PMC1481516

[50] Franklin RB Zou J Yu Z Costello LC. EAAC1 is expressed in rat and human prostate epithelial cells; functions as a high-affinity l-aspartate transporter; and is regulated by prolactin and testosterone. BMC Biochem (2006) 7:10. doi: 10.1186/1471-2091-7-10 16566829 PMC1456973

[51] Uzzo RG Leavis P Hatch W Gabai VL Dulin N Zvartau N. Zinc inhibits nuclear factor-kappa b activation and sensitizes prostate cancer cells to cytotoxic agents. Clin Cancer Res (2002) 8(11):3579–83. doi: 10.1093/carcin/23.11.1963 12429649

[52] Cutruzzolà F Giardina G Marani M Macone A Paiardini A Rinaldo S. Glucose metabolism in the progression of prostate cancer. Front Physiol (2017) 8:97. doi: 10.3389/fphys.2017.00097 28270771 PMC5318430

[53] Makhov PB Golovine KV Kutikov A Canter DJ Rybko VA Roshchin DA. Reversal of epigenetic silencing of AP-2alpha results in increased zinc uptake in DU-145 and LNCaP prostate cancer cells. Carcinogenesis (2011) 32(12):1773–81. doi: 10.1093/carcin/bgr212 PMC3220607 21940908

[54] Kawada K Nakamoto Y Kawada M Hida K Matsumoto T Murakami T. Relationship between 18F-fluorodeoxyglucose accumulation and KRAS/BRAF mutations in colorectal cancer. Clin Cancer Res (2012) 18(6):1696–703. doi: 10.1158/1078-0432.Ccr-11-1909 22282467

[55] Feng Y Xiong Y Qiao T Li X Jia L Han Y. Lactate dehydrogenase a: A key player in carcinogenesis and potential target in cancer therapy. Cancer Med (2018) 7(12):6124–36. doi: 10.1002/cam4.1820 PMC6308051 30403008

[56] Wang J Yuan W Chen Z Wu S Chen J Ge J. Overexpression of G6PD is associated with poor clinical outcome in gastric cancer. Tumour Biol (2012) 33(1):95–101. doi: 10.1007/s13277-011-0251-9 22012600

[57] Satoh K Yachida S Sugimoto M Oshima M Nakagawa T Akamoto S. Global metabolic reprogramming of colorectal cancer occurs at adenoma stage and is induced by MYC. Proc Natl Acad Sci U.S.A. (2017) 114(37):E7697–e706. doi: 10.1073/pnas.1710366114 PMC5604037 28847964

[58] Sebastián C Zwaans BM Silberman DM Gymrek M Goren A Zhong L. The histone deacetylase SIRT6 is a tumor suppressor that controls cancer metabolism. Cell (2012) 151(6):1185–99. doi: 10.1016/j.cell.2012.10.047 PMC3526953 23217706

[59] Jin L Zhou Y. Crucial role of the pentose phosphate pathway in malignant tumors. Oncol Lett (2019) 17(5):4213–21. doi: 10.3892/ol.2019.10112 PMC6444344 30944616

[60] Cheung EC Athineos D Lee P Ridgway RA Lambie W Nixon C. TIGAR is required for efficient intestinal regeneration and tumorigenesis. Dev Cell (2013) 25(5):463–77. doi: 10.1016/j.devcel.2013.05.001 PMC3682186 23726973

[61] Huang Q Tan Y Yin P Ye G Gao P Lu X. Metabolic characterization of hepatocellular carcinoma using nontargeted tissue metabolomics. Cancer Res (2013) 73(16):4992–5002. doi: 10.1158/0008-5472.Can-13-0308 23824744

[62] Satriano L Lewinska M Rodrigues PM Banales JM Andersen JB. Metabolic rearrangements in primary liver cancers: cause and consequences. Nat Rev Gastroenterol Hepatol (2019) 16(12):748–66. doi: 10.1038/s41575-019-0217-8 31666728

[63] Yang J Nie J Ma X Wei Y Peng Y Wei X. Targeting PI3K in cancer: mechanisms and advances in clinical trials. Mol Cancer (2019) 18(1):26. doi: 10.1186/s12943-019-0954-x 30782187 PMC6379961

[64] Long YC Zierath JR. AMP-activated protein kinase signaling in metabolic regulation. J Clin Invest (2006) 116(7):1776–83. doi: 10.1172/jci29044 PMC1483147 16823475

[65] Luo Z Zang M Guo W. AMPK as a metabolic tumor suppressor: control of metabolism and cell growth. Future Oncol (2010) 6(3):457–70. doi: 10.2217/fon.09.174 PMC2854547 20222801

[66] McCubrey JA Steelman LS Chappell WH Abrams SL Montalto G Cervello M. Mutations and deregulation of Ras/Raf/MEK/ERK and PI3K/PTEN/Akt/mTOR cascades which alter therapy response. Oncotarget (2012) 3(9):954–87. doi: 10.18632/oncotarget.652 PMC3660063 23006971

[67] Gingras AC Raught B Sonenberg N. Regulation of translation initiation by FRAP/mTOR. Genes Dev (2001) 15(7):807–26. doi: 10.1101/gad.887201 11297505

[68] Hollander MC Blumenthal GM Dennis PA. PTEN loss in the continuum of common cancers, rare syndromes and mouse models. Nat Rev Cancer (2011) 11(4):289–301. doi: 10.1038/nrc3037 21430697 PMC6946181

[69] Dang CV. Links between metabolism and cancer. Genes Dev (2012) 26(9):877–90. doi: 10.1101/gad.189365.112 PMC3347786 22549953

[70] Gottlob K Majewski N Kennedy S Kandel E Robey RB Hay N. Inhibition of early apoptotic events by Akt/PKB is dependent on the first committed step of glycolysis and mitochondrial hexokinase. Genes Dev (2001) 15(11):1406–18. doi: 10.1101/gad.889901 PMC312709 11390360

[71] DeBerardinis RJ. Is cancer a disease of abnormal cellular metabolism? new angles on an old idea. Genet Med (2008) 10(11):767–77. doi: 10.1097/GIM.0b013e31818b0d9b PMC2782690 18941420

[72] Denko NC. Hypoxia, HIF1 and glucose metabolism in the solid tumour. Nat Rev Cancer (2008) 8(9):705–13. doi: 10.1038/nrc2468 19143055

[73] Phan LM Yeung SC Lee MH. Cancer metabolic reprogramming: importance, main features, and potentials for precise targeted anti-cancer therapies. Cancer Biol Med (2014) 11(1):1–19. doi: 10.7497/j.issn.2095-3941.2014.01.001 24738035 PMC3969803

[74] Vander Heiden MG Cantley LC Thompson CB. Understanding the warburg effect: the metabolic requirements of cell proliferation. Science (2009) 324(5930):1029–33. doi: 10.1126/science.1160809 PMC2849637 19460998

[75] Zahra K Dey T Ashish Mishra SP Pandey U. Pyruvate kinase M2 and cancer: The role of PKM2 in promoting tumorigenesis. Front Oncol (2020) 10:159. doi: 10.3389/fonc.2020.00159 32195169 PMC7061896

[76] Wong N De Melo J Tang D. PKM2, a central point of regulation in cancer metabolism. Int J Cell Biol (2013) 2013:242513. doi: 10.1155/2013/242513 23476652 PMC3586519

[77] Conrad PW Freeman TL Beitner-Johnson D Millhorn DE. EPAS1 trans-activation during hypoxia requires p42/p44 MAPK. J Biol Chem (1999) 274(47):33709–13. doi: 10.1074/jbc.274.47.33709 10559262

[78] Jiang BH Jiang G Zheng JZ Lu Z Hunter T Vogt PK. Phosphatidylinositol 3-kinase signaling controls levels of hypoxia-inducible factor 1. Cell Growth Differ (2001) 12(7):363–9. 11457733

[79] Lv X Li J Zhang C Hu T Li S He S. The role of hypoxia-inducible factors in tumor angiogenesis and cell metabolism. Genes Dis (2017) 4(1):19–24. doi: 10.1016/j.gendis.2016.11.003 30258904 PMC6136595

[80] Semenza G. Signal transduction to hypoxia-inducible factor 1. Biochem Pharmacol (2002) 64(5-6):993–8. doi: 10.1016/s0006-2952(02)01168-1 12213597

[81] Guzy RD Hoyos B Robin E Chen H Liu L Mansfield KD. Mitochondrial complex III is required for hypoxia-induced ROS production and cellular oxygen sensing. Cell Metab (2005) 1(6):401–8. doi: 10.1016/j.cmet.2005.05.001 16054089

[82] Kado K Forsyth A Patel PR Schwartz JA. Dietary supplements and natural products in breast cancer trials. Front Biosci (Elite Ed) (2012) 4(1):546–67. doi: 10.2741/399 22201894

[83] Klimova T Chandel NS. Mitochondrial complex III regulates hypoxic activation of HIF. Cell Death Differ (2008) 15(4):660–6. doi: 10.1038/sj.cdd.4402307 18219320

[84] Sang N Stiehl DP Bohensky J Leshchinsky I Srinivas V Caro J. MAPK signaling up-regulates the activity of hypoxia-inducible factors by its effects on p300. J Biol Chem (2003) 278(16):14013–9. doi: 10.1074/jbc.M209702200 PMC4518846 12588875

[85] Lu J Tan M Cai Q. The warburg effect in tumor progression: mitochondrial oxidative metabolism as an anti-metastasis mechanism. Cancer Lett (2015) 356(2 Pt A):156–64. doi: 10.1016/j.canlet.2014.04.001 PMC4195816 24732809

[86] Kim JW Tchernyshyov I Semenza GL Dang CV. HIF-1-mediated expression of pyruvate dehydrogenase kinase: a metabolic switch required for cellular adaptation to hypoxia. Cell Metab (2006) 3(3):177–85. doi: 10.1016/j.cmet.2006.02.002 16517405

[87] Papandreou I Cairns RA Fontana L Lim AL Denko NC. HIF-1 mediates adaptation to hypoxia by actively downregulating mitochondrial oxygen consumption. Cell Metab (2006) 3(3):187–97. doi: 10.1016/j.cmet.2006.01.012 16517406

[88] Lee M Chen GT Puttock E Wang K Edwards RA Waterman ML. Mathematical modeling links wnt signaling to emergent patterns of metabolism in colon cancer. Mol Syst Biol (2017) 13(2):912. doi: 10.15252/msb.20167386 28183841 PMC5327728

[89] Ahmad F Sun Q Patel D Stommel JM. Cholesterol metabolism: A potential therapeutic target in glioblastoma. Cancers (Basel) (2019) 11(2):146. doi: 10.3390/cancers11020146 30691162 PMC6406281

[90] Sprowl-Tanio S Habowski AN Pate KT McQuade MM Wang K Edwards RA. Lactate/pyruvate transporter MCT-1 is a direct wnt target that confers sensitivity to 3-bromopyruvate in colon cancer. Cancer Metab (2016) 4:1–18. doi: 10.1186/s40170-016-0159-3 PMC5046889 27729975

[91] Mathupala SP Heese C Pedersen PL. Glucose catabolism in cancer cells. the type II hexokinase promoter contains functionally active response elements for the tumor suppressor p53. J Biol Chem (1997) 272(36):22776–80. doi: 10.1074/jbc.272.36.22776 9278438

[92] Schwartzenberg-Bar-Yoseph F Armoni M Karnieli E. The tumor suppressor p53 down-regulates glucose transporters GLUT1 and GLUT4 gene expression. Cancer Res (2004) 64(7):2627–33. doi: 10.1158/0008-5472.can-03-0846 15059920

[93] Zhang C Liu J Liang Y Wu R Zhao Y Hong X. Tumour-associated mutant p53 drives the warburg effect. Nat Commun (2013) 4:2935. doi: 10.1038/ncomms3935 24343302 PMC3969270

[94] Zhou G Wang J Zhao M Xie TX Tanaka N Sano D. Gain-of-function mutant p53 promotes cell growth and cancer cell metabolism *via* inhibition of AMPK activation. Mol Cell (2014) 54(6):960–74. doi: 10.1016/j.molcel.2014.04.024 PMC4067806 24857548

[95] Zhang H Lu C Fang M Yan W Chen M Ji Y. HIF-1α activates hypoxia-induced PFKFB4 expression in human bladder cancer cells. Biochem Biophys Res Commun (2016) 476(3):146–52. doi: 10.1016/j.bbrc.2016.05.026 27181362

[96] Hay N. Reprogramming glucose metabolism in cancer: can it be exploited for cancer therapy? Nat Rev Cancer (2016) 16(10):635–49. doi: 10.1038/nrc.2016.77 PMC5516800 27634447

[97] Moreno-Sánchez R Rodríguez-Enríquez S Marín-Hernández A Saavedra E. Energy metabolism in tumor cells. FEBS J (2007) 274(6):1393–418. doi: 10.1111/j.1742-4658.2007.05686.x 17302740

[98] Wei Z Liu X Cheng C Yu W Yi P. Metabolism of amino acids in cancer. Front Cell Dev Biol (2020) 8:603837. doi: 10.3389/fcell.2020.603837 33511116 PMC7835483

[99] Di Tommaso L Franchi G Park YN Fiamengo B Destro A Morenghi E. Diagnostic value of HSP70, glypican 3, and glutamine synthetase in hepatocellular nodules in cirrhosis. Hepatology (2007) 45(3):725–34. doi: 10.1002/hep.21531 17326147

[100] Long J Wang H Lang Z Wang T Long M Wang B. Expression level of glutamine synthetase is increased in hepatocellular carcinoma and liver tissue with cirrhosis and chronic hepatitis b. Hepatol Int (2011) 5(2):698–706. doi: 10.1007/s12072-010-9230-2 21484108 PMC3090553

[101] Marin-Valencia I Yang C Mashimo T Cho S Baek H Yang XL. Analysis of tumor metabolism reveals mitochondrial glucose oxidation in genetically diverse human glioblastomas in the mouse brain *in vivo* . Cell Metab (2012) 15(6):827–37. doi: 10.1016/j.cmet.2012.05.001 PMC3372870 22682223

[102] Wang J Wang H Liu AF Fang CG Hao JG Wang ZH. Lactate dehydrogenase a negatively regulated by miRNAs promotes aerobic glycolysis and is increased in colorectal cancer. Oncotarget (2015) 6(23):19456–68. doi: 10.18632/oncotarget.3318 PMC4637298 26062441

[103] Altman BJ Stine ZE Dang CV. From Krebs to clinic: glutamine metabolism to cancer therapy. Nat Rev Cancer (2016) 16(10):619–34. doi: 10.1038/nrc.2016.71 PMC5484415 27492215

[104] Curthoys NP Watford M. Regulation of glutaminase activity and glutamine metabolism. Annu Rev Nutr (1995) 15:133–59. doi: 10.1146/annurev.nu.15.070195.001025 8527215

[105] Jiang Z Zhang C Gan L Jia Y Xiong Y Chen Y. iTRAQ-based quantitative proteomics approach identifies novel diagnostic biomarkers that were essential for glutamine metabolism and redox homeostasis for gastric cancer. Proteomics Clin Appl (2019) 13(4):e1800038. doi: 10.1002/prca.201800038 30485682

[106] Song Z Wei B Lu C Li P Chen L. Glutaminase sustains cell survival *via* the regulation of glycolysis and glutaminolysis in colorectal cancer. Oncol Lett (2017) 14(3):3117–23. doi: 10.3892/ol.2017.6538 PMC5588174 28928849

[107] Willems L Jacque N Jacquel A Neveux N Maciel TT Lambert M. Inhibiting glutamine uptake represents an attractive new strategy for treating acute myeloid leukemia. Blood (2013) 122(20):3521–32. doi: 10.1182/blood-2013-03-493163 PMC3829119 24014241

[108] Jiménez-González V Ogalla-García E García-Quintanilla M García-Quintanilla A. Deciphering GRINA/Lifeguard1: Nuclear location, Ca(2+) homeostasis and vesicle transport. Int J Mol Sci (2019) 20(16):4005. doi: 10.3390/ijms20164005 31426446 PMC6719933

[109] Xu DH Li Q Hu H Ni B Liu X Huang C. Transmembrane protein GRINA modulates aerobic glycolysis and promotes tumor progression in gastric cancer. J Exp Clin Cancer Res (2018) 37(1):308. doi: 10.1186/s13046-018-0974-1 30541591 PMC6292005

[110] Adebayo Michael AO Ko S Tao J Moghe A Yang H Xu M. Inhibiting glutamine-dependent mTORC1 activation ameliorates liver cancers driven by β-catenin mutations. Cell Metab (2019) 29(5):1135–50.e6. doi: 10.1016/j.cmet.2019.01.002 30713111 PMC6506359

[111] Chen Q Ye L Fan J Zhang X Wang H Liao S. Autophagy suppression potentiates the anti-glioblastoma effect of asparaginase *in vitro* and *in vivo* . Oncotarget (2017) 8(53):91052–66. doi: 10.18632/oncotarget.19409 PMC5710905 29207624

[112] Toda K Kawada K Iwamoto M Inamoto S Sasazuki T Shirasawa S. Metabolic alterations caused by KRAS mutations in colorectal cancer contribute to cell adaptation to glutamine depletion by upregulation of asparagine synthetase. Neoplasia (2016) 18(11):654–65. doi: 10.1016/j.neo.2016.09.004 PMC5071549 27764698

[113] Jiang J Batra S Zhang J. Asparagine: A metabolite to be targeted in cancers. Metabolites (2021) 11(6):402. doi: 10.3390/metabo11060402 34205460 PMC8234323

[114] Liu J Xia X Huang P. xCT: A critical molecule that links cancer metabolism to redox signaling. Mol Ther (2020) 28(11):2358–66. doi: 10.1016/j.ymthe.2020.08.021 PMC7647670 32931751

[115] Zhang B Dong LW Tan YX Zhang J Pan YF Yang C. Asparagine synthetase is an independent predictor of surgical survival and a potential therapeutic target in hepatocellular carcinoma. Br J Cancer (2013) 109(1):14–23. doi: 10.1038/bjc.2013.293 23764751 PMC3708586

[116] Karpel-Massler G Ramani D Shu C Halatsch ME Westhoff MA Bruce JN. Metabolic reprogramming of glioblastoma cells by l-asparaginase sensitizes for apoptosis *in vitro* and *in vivo* . Oncotarget (2016) 7(23):33512–28. doi: 10.18632/oncotarget.9257 PMC5085099 27172899

[117] Amelio I Cutruzzolá F Antonov A Agostini M Melino G. Serine and glycine metabolism in cancer. Trends Biochem Sci (2014) 39(4):191–8. doi: 10.1016/j.tibs.2014.02.004 PMC3989988 24657017

[118] Mehrmohamadi M Mentch LK Clark AG Locasale JW. Integrative modelling of tumour DNA methylation quantifies the contribution of metabolism. Nat Commun (2016) 7:13666. doi: 10.1038/ncomms13666 27966532 PMC5171841

[119] Puccini A Berger MD Naseem M Tokunaga R Battaglin F Cao S. Colorectal cancer: epigenetic alterations and their clinical implications. Biochim Biophys Acta Rev Cancer (2017) 1868(2):439–48. doi: 10.1016/j.bbcan.2017.09.003 PMC5757873 28939182

[120] De Marchi T Timmermans MA Sieuwerts AM Smid M Look MP Grebenchtchikov N. Phosphoserine aminotransferase 1 is associated to poor outcome on tamoxifen therapy in recurrent breast cancer. Sci Rep (2017) 7(1):2099. doi: 10.1038/s41598-017-02296-w 28522855 PMC5437008

[121] Vié N Copois V Bascoul-Mollevi C Denis V Bec N Robert B. Overexpression of phosphoserine aminotransferase PSAT1 stimulates cell growth and increases chemoresistance of colon cancer cells. Mol Cancer (2008) 7(1):14. doi: 10.1186/1476-4598-7-14 18221502 PMC2245978

[122] Ma L Tao Y Duran A Llado V Galvez A Barger JF. Control of nutrient stress-induced metabolic reprogramming by PKCζ in tumorigenesis. Cell (2013) 152(3):599–611. doi: 10.1016/j.cell.2012.12.028 23374352 PMC3963830

[123] Qiu F Huang J Sui M. Targeting arginine metabolism pathway to treat arginine-dependent cancers. Cancer Lett (2015) 364(1):1–7. doi: 10.1016/j.canlet.2015.04.020 25917076

[124] White RM Cech J Ratanasirintrawoot S Lin CY Rahl PB Burke CJ. DHODH modulates transcriptional elongation in the neural crest and melanoma. Nature (2011) 471(7339):518–22. doi: 10.1038/nature09882 PMC3759979 21430780

[125] Hsueh EC Knebel SM Lo WH Leung YC Cheng PN Hsueh CT. Deprivation of arginine by recombinant human arginase in prostate cancer cells. J Hematol Oncol (2012) 5:17. doi: 10.1186/1756-8722-5-17 22546217 PMC3403903

[126] Kim RH Coates JM Bowles TL McNerney GP Sutcliffe J Jung JU. Arginine deiminase as a novel therapy for prostate cancer induces autophagy and caspase-independent apoptosis. Cancer Res (2009) 69(2):700–8. doi: 10.1158/0008-5472.Can-08-3157 PMC2629384 19147587

[127] Tomlinson BK Thomson JA Bomalaski JS Diaz M Akande T Mahaffey N. Phase I trial of arginine deprivation therapy with ADI-PEG 20 plus docetaxel in patients with advanced malignant solid tumors. Clin Cancer Res (2015) 21(11):2480–6. doi: 10.1158/1078-0432.Ccr-14-2610 PMC4452427 25739672

[128] Hattori A Tsunoda M Konuma T Kobayashi M Nagy T Glushka J. Cancer progression by reprogrammed BCAA metabolism in myeloid leukaemia. Nature (2017) 545(7655):500–4. doi: 10.1038/nature22314 PMC5554449 28514443

[129] Wang Y Zhang J Ren S Sun D Huang HY Wang H. Branched-chain amino acid metabolic reprogramming orchestrates drug resistance to EGFR tyrosine kinase inhibitors. Cell Rep (2019) 28(2):512–25.e6. doi: 10.1016/j.celrep.2019.06.026 31291585

[130] Zhang W Trachootham D Liu J Chen G Pelicano H Garcia-Prieto C. Stromal control of cystine metabolism promotes cancer cell survival in chronic lymphocytic leukaemia. Nat Cell Biol (2012) 14(3):276–86. doi: 10.1038/ncb2432 PMC3290742 22344033

[131] Phillips CM Zatarain JR Nicholls ME Porter C Widen SG Thanki K. Upregulation of cystathionine-β-Synthase in colonic epithelia reprograms metabolism and promotes carcinogenesis. Cancer Res (2017) 77(21):5741–54. doi: 10.1158/0008-5472.Can-16-3480 PMC5668191 28923859

[132] Gao W Chen L Ma Z Du Z Zhao Z Hu Z. Isolation and phenotypic characterization of colorectal cancer stem cells with organ-specific metastatic potential. Gastroenterology (2013) 145(3):636–46.e5. doi: 10.1053/j.gastro.2013.05.049 23747337

[133] Wu Z Wei D Gao W Xu Y Hu Z Ma Z. TPO-induced metabolic reprogramming drives liver metastasis of colorectal cancer CD110+ tumor-initiating cells. Cell Stem Cell (2015) 17(1):47–59. doi: 10.1016/j.stem.2015.05.016 26140605

[134] Munn DH Sharma MD Baban B Harding HP Zhang Y Ron D. GCN2 kinase in T cells mediates proliferative arrest and anergy induction in response to indoleamine 2,3-dioxygenase. Immunity (2005) 22(5):633–42. doi: 10.1016/j.immuni.2005.03.013 15894280

[135] Ravishankar B Liu H Shinde R Chaudhary K Xiao W Bradley J. The amino acid sensor GCN2 inhibits inflammatory responses to apoptotic cells promoting tolerance and suppressing systemic autoimmunity. Proc Natl Acad Sci U.S.A. (2015) 112(34):10774–9. doi: 10.1073/pnas.1504276112 PMC4553766 26261340

[136] Fallarino F Grohmann U You S McGrath BC Cavener DR Vacca C. The combined effects of tryptophan starvation and tryptophan catabolites down-regulate T cell receptor zeta-chain and induce a regulatory phenotype in naive T cells. J Immunol (2006) 176(11):6752–61. doi: 10.4049/jimmunol.176.11.6752 16709834

[137] Mishra P Ambs S. Metabolic signatures of human breast cancer. Mol Cell Oncol (2015) 2(3):e992217. doi: 10.4161/23723556.2014.992217 26005711 PMC4438683

[138] Mor A Tankiewicz-Kwedlo A Pawlak D. Kynurenines as a novel target for the treatment of malignancies. Pharm (Basel) (2021) 14(7):606. doi: 10.3390/ph14070606 PMC8308824 34201791

[139] Dossou AS Basu A. The emerging roles of mTORC1 in macromanaging autophagy. Cancers (Basel) (2019) 11(10):1422. doi: 10.3390/cancers11101422 31554253 PMC6826502

[140] Conde E Suarez-Gauthier A García-García E Lopez-Rios F Lopez-Encuentra A García-Lujan R. Specific pattern of LKB1 and phospho-acetyl-CoA carboxylase protein immunostaining in human normal tissues and lung carcinomas. Hum Pathol (2007) 38(9):1351–60. doi: 10.1016/j.humpath.2007.01.022 17521700

[141] Budanov AV Karin M. p53 target genes sestrin1 and sestrin2 connect genotoxic stress and mTOR signaling. Cell (2008) 134(3):451–60. doi: 10.1016/j.cell.2008.06.028 PMC2758522 18692468

[142] Yoshida S Pacitto R Inoki K Swanson J. Macropinocytosis, mTORC1 and cellular growth control. Cell Mol Life Sci (2018) 75(7):1227–39. doi: 10.1007/s00018-017-2710-y PMC5843684 29119228

[143] Han JM Jeong SJ Park MC Kim G Kwon NH Kim HK. Leucyl-tRNA synthetase is an intracellular leucine sensor for the mTORC1-signaling pathway. Cell (2012) 149(2):410–24. doi: 10.1016/j.cell.2012.02.044 22424946

[144] Jung J Genau HM Behrends C. Amino acid-dependent mTORC1 regulation by the lysosomal membrane protein SLC38A9. Mol Cell Biol (2015) 35(14):2479–94. doi: 10.1128/mcb.00125-15 PMC4475919 25963655

[145] Rebsamen M Pochini L Stasyk T de Araújo ME Galluccio M Kandasamy RK. SLC38A9 is a component of the lysosomal amino acid sensing machinery that controls mTORC1. Nature (2015) 519(7544):477–81. doi: 10.1038/nature14107 PMC4376665 25561175

[146] Wang S Tsun ZY Wolfson RL Shen K Wyant GA Plovanich ME. Metabolism. lysosomal amino acid transporter SLC38A9 signals arginine sufficiency to mTORC1. Science (2015) 347(6218):188–94. doi: 10.1126/science.1257132 PMC4295826 25567906

[147] Chantranupong L Scaria SM Saxton RA Gygi MP Shen K Wyant GA. The CASTOR proteins are arginine sensors for the mTORC1 pathway. Cell (2016) 165(1):153–64. doi: 10.1016/j.cell.2016.02.035 PMC4808398 26972053

[148] Gu X Orozco JM Saxton RA Condon KJ Liu GY Krawczyk PA. SAMTOR is an s-adenosylmethionine sensor for the mTORC1 pathway. Science (2017) 358(6364):813–8. doi: 10.1126/science.aao3265 PMC5747364 29123071

[149] Saxton RA Sabatini DM. mTOR signaling in growth, metabolism, and disease. Cell (2017) 168(6):960–76. doi: 10.1016/j.cell.2017.02.004 PMC5394987 28283069

[150] Baenke F Peck B Miess H Schulze A. Hooked on fat: the role of lipid synthesis in cancer metabolism and tumour development. Dis Model Mech (2013) 6(6):1353–63. doi: 10.1242/dmm.011338 PMC3820259 24203995

[151] Beloribi-Djefaflia S Vasseur S Guillaumond F. Lipid metabolic reprogramming in cancer cells. Oncogenesis (2016) 5(1):e189. doi: 10.1038/oncsis.2015.49 26807644 PMC4728678

[152] Notarnicola M Tutino V Calvani M Lorusso D Guerra V Caruso MG. Serum levels of fatty acid synthase in colorectal cancer patients are associated with tumor stage. J Gastrointest Cancer (2012) 43(3):508–11. doi: 10.1007/s12029-011-9300-2 21727995

[153] Accioly MT Pacheco P Maya-Monteiro CM Carrossini N Robbs BK Oliveira SS. Lipid bodies are reservoirs of cyclooxygenase-2 and sites of prostaglandin-E2 synthesis in colon cancer cells. Cancer Res (2008) 68(6):1732–40. doi: 10.1158/0008-5472.Can-07-1999 18339853

[154] Menendez JA Lupu R. Fatty acid synthase and the lipogenic phenotype in cancer pathogenesis. Nat Rev Cancer (2007) 7(10):763–77. doi: 10.1038/nrc2222 17882277

[155] Tennant DA Durán RV Gottlieb E. Targeting metabolic transformation for cancer therapy. Nat Rev Cancer (2010) 10(4):267–77. doi: 10.1038/nrc2817 20300106

[156] Calvisi DF Wang C Ho C Ladu S Lee SA Mattu S. Increased lipogenesis, induced by AKT-mTORC1-RPS6 signaling, promotes development of human hepatocellular carcinoma. Gastroenterology (2011) 140(3):1071–83. doi: 10.1053/j.gastro.2010.12.006 PMC3057329 21147110

[157] Zaidi N Lupien L Kuemmerle NB Kinlaw WB Swinnen JV Smans K. Lipogenesis and lipolysis: the pathways exploited by the cancer cells to acquire fatty acids. Prog Lipid Res (2013) 52(4):585–9. doi: 10.1016/j.plipres.2013.08.005 PMC4002264 24001676

[158] Dai XY Zhuang LH Wang DD Zhou TY Chang LL Gai RH. Nuclear translocation and activation of YAP by hypoxia contributes to the chemoresistance of SN38 in hepatocellular carcinoma cells. Oncotarget (2016) 7(6):6933–47. doi: 10.18632/oncotarget.6903 PMC4872759 26771844

[159] Bathaie SZ Ashrafi M Azizian M Tamanoi F. Mevalonate pathway and human cancers. Curr Mol Pharmacol (2017) 10(2):77–85. doi: 10.2174/1874467209666160112123205 26758953

[160] Hashimoto A Oikawa T Hashimoto S Sugino H Yoshikawa A Otsuka Y. P53- and mevalonate pathway-driven malignancies require Arf6 for metastasis and drug resistance. J Cell Biol (2016) 213(1):81–95. doi: 10.1083/jcb.201510002 27044891 PMC4828690

[161] Chushi L Wei W Kangkang X Yongzeng F Ning X Xiaolei C. HMGCR is up-regulated in gastric cancer and promotes the growth and migration of the cancer cells. Gene (2016) 587(1):42–7. doi: 10.1016/j.gene.2016.04.029 27085483

[162] Shimano H Sato R. SREBP-regulated lipid metabolism: convergent physiology - divergent pathophysiology. Nat Rev Endocrinol (2017) 13(12):710–30. doi: 10.1038/nrendo.2017.91 28849786

[163] Mashima T Seimiya H Tsuruo T. *De novo* fatty-acid synthesis and related pathways as molecular targets for cancer therapy. Br J Cancer (2009) 100(9):1369–72. doi: 10.1038/sj.bjc.6605007 PMC2694429 19352381

[164] Yang YA Han WF Morin PJ Chrest FJ Pizer ES. Activation of fatty acid synthesis during neoplastic transformation: role of mitogen-activated protein kinase and phosphatidylinositol 3-kinase. Exp Cell Res (2002) 279(1):80–90. doi: 10.1006/excr.2002.5600 12213216

[165] Deep G Schlaepfer IR. Aberrant lipid metabolism promotes prostate cancer: Role in cell survival under hypoxia and extracellular vesicles biogenesis. Int J Mol Sci (2016) 17(7):1061. doi: 10.3390/ijms17071061 27384557 PMC4964437

[166] Geng F Cheng X Wu X Yoo JY Cheng C Guo JY. Inhibition of SOAT1 suppresses glioblastoma growth *via* blocking SREBP-1-Mediated lipogenesis. Clin Cancer Res (2016) 22(21):5337–48. doi: 10.1158/1078-0432.Ccr-15-2973 PMC5093025 27281560

[167] Barber CN Raben DM. Lipid metabolism crosstalk in the brain: Glia and neurons. Front Cell Neurosci (2019) 13:212. doi: 10.3389/fncel.2019.00212 31164804 PMC6536584

[168] Pirmoradi L Seyfizadeh N Ghavami S Zeki AA Shojaei S. Targeting cholesterol metabolism in glioblastoma: a new therapeutic approach in cancer therapy. J Investig Med (2019) 67(4):715–9. doi: 10.1136/jim-2018-000962 30765502

[169] Lewis CA Brault C Peck B Bensaad K Griffiths B Mitter R. SREBP maintains lipid biosynthesis and viability of cancer cells under lipid- and oxygen-deprived conditions and defines a gene signature associated with poor survival in glioblastoma multiforme. Oncogene (2015) 34(40):5128–40. doi: 10.1038/onc.2014.439 25619842

[170] Patel D Ahmad F Kambach DM Sun Q Halim AS Kramp T. LXRβ controls glioblastoma cell growth, lipid balance, and immune modulation independently of ABCA1. Sci Rep (2019) 9(1):15458. doi: 10.1038/s41598-019-51865-8 31664073 PMC6820787

[171] Lin H Patel S Affleck VS Wilson I Turnbull DM Joshi AR. Fatty acid oxidation is required for the respiration and proliferation of malignant glioma cells. Neuro Oncol (2017) 19(1):43–54. doi: 10.1093/neuonc/now128 27365097 PMC5193020

[172] Hale JS Otvos B Sinyuk M Alvarado AG Hitomi M Stoltz K. Cancer stem cell-specific scavenger receptor CD36 drives glioblastoma progression. Stem Cells (2014) 32(7):1746–58. doi: 10.1002/stem.1716 PMC4063873 24737733

[173] Grube S Göttig T Freitag D Ewald C Kalff R Walter J. Selection of suitable reference genes for expression analysis in human glioma using RT-qPCR. J Neurooncol (2015) 123(1):35–42. doi: 10.1007/s11060-015-1772-7 25862007

[174] Lancaster MA Renner M Martin CA Wenzel D Bicknell LS Hurles ME. Cerebral organoids model human brain development and microcephaly. Nature (2013) 501(7467):373–9. doi: 10.1038/nature12517 PMC3817409 23995685

[175] D'Erchia AM Tullo A Lefkimmiatis K Saccone C Sbisà E. The fatty acid synthase gene is a conserved p53 family target from worm to human. Cell Cycle (2006) 5(7):750–8. doi: 10.4161/cc.5.7.2622 16582625

[176] Lu S Archer MC. Sp1 coordinately regulates *de novo* lipogenesis and proliferation in cancer cells. Int J Cancer (2010) 126(2):416–25. doi: 10.1002/ijc.24761 19621387

[177] Martel PM Bingham CM McGraw CJ Baker CL Morganelli PM Meng ML. S14 protein in breast cancer cells: direct evidence of regulation by SREBP-1c, superinduction with progestin, and effects on cell growth. Exp Cell Res (2006) 312(3):278–88. doi: 10.1016/j.yexcr.2005.10.022 16300755

[178] Graner E Tang D Rossi S Baron A Migita T Weinstein LJ. The isopeptidase USP2a regulates the stability of fatty acid synthase in prostate cancer. Cancer Cell (2004) 5(3):253–61. doi: 10.1016/s1535-6108(04)00055-8 15050917

[179] Yoon S Lee MY Park SW Moon JS Koh YK Ahn YH. Up-regulation of acetyl-CoA carboxylase alpha and fatty acid synthase by human epidermal growth factor receptor 2 at the translational level in breast cancer cells. J Biol Chem (2007) 282(36):26122–31. doi: 10.1074/jbc.M702854200 17631500

[180] Luo DX Peng XH Xiong Y Liao DF Cao D Li L. Dual role of insulin-like growth factor-1 in acetyl-CoA carboxylase-alpha activity in human colon cancer cells HCT-8: downregulating its expression and phosphorylation. Mol Cell Biochem (2011) 357(1-2):255–62. doi: 10.1007/s11010-011-0896-0 21638027

[181] Wang C Shi M Ji J Cai Q Zhao Q Jiang J. Stearoyl-CoA desaturase 1 (SCD1) facilitates the growth and anti-ferroptosis of gastric cancer cells and predicts poor prognosis of gastric cancer. Aging (Albany N Y) (2020) 12(15):15374–91. doi: 10.18632/aging.103598 PMC7467382 32726752

[182] Hardie DG Ross FA Hawley SA. AMPK: a nutrient and energy sensor that maintains energy homeostasis. Nat Rev Mol Cell Biol (2012) 13(4):251–62. doi: 10.1038/nrm3311 PMC5726489 22436748

[183] Wang YN Zeng ZL Lu J Wang Y Liu ZX He MM. CPT1A-mediated fatty acid oxidation promotes colorectal cancer cell metastasis by inhibiting anoikis. Oncogene (2018) 37(46):6025–40. doi: 10.1038/s41388-018-0384-z 29995871

[184] Csanadi A Kayser C Donauer M Gumpp V Aumann K Rawluk J. Prognostic value of malic enzyme and ATP-citrate lyase in non-small cell lung cancer of the young and the elderly. PloS One (2015) 10(5):e0126357. doi: 10.1371/journal.pone.0126357 25962060 PMC4427316

[185] Beyaz S Mana MD Roper J Kedrin D Saadatpour A Hong SJ. High-fat diet enhances stemness and tumorigenicity of intestinal progenitors. Nature (2016) 531(7592):53–8. doi: 10.1038/nature17173 PMC4846772 26935695

[186] Kim KH Shin HJ Kim K Choi HM Rhee SH Moon HB. Hepatitis b virus X protein induces hepatic steatosis *via* transcriptional activation of SREBP1 and PPARgamma. Gastroenterology (2007) 132(5):1955–67. doi: 10.1053/j.gastro.2007.03.039 17484888

[187] Yang F Yan S He Y Wang F Song S Guo Y. Expression of hepatitis b virus proteins in transgenic mice alters lipid metabolism and induces oxidative stress in the liver. J Hepatol (2008) 48(1):12–9. doi: 10.1016/j.jhep.2007.06.021 18037187

[188] Luo X Cheng C Tan Z Li N Tang M Yang L. Emerging roles of lipid metabolism in cancer metastasis. Mol Cancer (2017) 16(1):1–10. doi: 10.1186/s12943-017-0646-3 28399876 PMC5387196

[189] Nomura DK Long JZ Niessen S Hoover HS Ng SW Cravatt BF. Monoacylglycerol lipase regulates a fatty acid network that promotes cancer pathogenesis. Cell (2010) 140(1):49–61. doi: 10.1016/j.cell.2009.11.027 20079333 PMC2885975

[190] Miyake T Parsons SJ. Functional interactions between choline kinase α, epidermal growth factor receptor and c-src in breast cancer cell proliferation. Oncogene (2012) 31(11):1431–41. doi: 10.1038/onc.2011.332 PMC3213328 21822308

[191] Paris L Cecchetti S Spadaro F Abalsamo L Lugini L Pisanu ME. Inhibition of phosphatidylcholine-specific phospholipase c downregulates HER2 overexpression on plasma membrane of breast cancer cells. Breast Cancer Res (2010) 12(3):1–16. doi: 10.1186/bcr2575 PMC2917016 20462431

[192] Thapa N Choi S Tan X Wise T Anderson RA. Phosphatidylinositol phosphate 5-kinase iγ and phosphoinositide 3-Kinase/Akt signaling couple to promote oncogenic growth. J Biol Chem (2015) 290(30):18843–54. doi: 10.1074/jbc.M114.596742 PMC4513138 26070568

[193] Uehara T Kikuchi H Miyazaki S Iino I Setoguchi T Hiramatsu Y. Overexpression of lysophosphatidylcholine acyltransferase 1 and concomitant lipid alterations in gastric cancer. Ann Surg Oncol (2016) 23 Suppl 2:S206–13. doi: 10.1245/s10434-015-4459-6 25752890

[194] Tao L Yu H Liang R Jia R Wang J Jiang K. Rev-erbα inhibits proliferation by reducing glycolytic flux and pentose phosphate pathway in human gastric cancer cells. Oncogenesis (2019) 8(10):57. doi: 10.1038/s41389-019-0168-5 31591390 PMC6779746

[195] Wang W Bai L Li W Cui J. The lipid metabolic landscape of cancers and new therapeutic perspectives. Front Oncol (2020) 10:605154. doi: 10.3389/fonc.2020.605154 33364199 PMC7753360

[196] Zhang F Du G. Dysregulated lipid metabolism in cancer. World J Biol Chem (2012) 3(8):167–74. doi: 10.4331/wjbc.v3.i8.167 PMC3430731 22937213

[197] Camici M Garcia-Gil M Pesi R Allegrini S Tozzi MG. Purine-metabolising enzymes and apoptosis in cancer. Cancers (Basel) (2019) 11(9):1354. doi: 10.3390/cancers11091354 31547393 PMC6769685

[198] Cinquin O Demongeot J. Roles of positive and negative feedback in biological systems. C R Biol (2002) 325(11):1085–95. doi: 10.1016/s1631-0691(02)01533-0 12506722

[199] Harris JC. Lesch-nyhan syndrome and its variants: examining the behavioral and neurocognitive phenotype. Curr Opin Psychiatry (2018) 31(2):96–102. doi: 10.1097/yco.0000000000000388 29227296

[200] Vanella A Barcellona ML Serra I Ragusa N Avola R Avitabile M. Effect of undernutrition on some enzymes involved in the salvage pathway of purine nucleotides in different regions of developing rat brain. Neurochem Res (1983) 8(2):151–8. doi: 10.1007/bf00963915 6856022

[201] Wang L. Mitochondrial purine and pyrimidine metabolism and beyond. Nucleosides Nucleotides Nucleic Acids (2016) 35(10-12):578–94. doi: 10.1080/15257770.2015.1125001 27906631

[202] Welin M Nordlund P. Understanding specificity in metabolic pathways–structural biology of human nucleotide metabolism. Biochem Biophys Res Commun (2010) 396(1):157–63. doi: 10.1016/j.bbrc.2010.04.054 20494131

[203] Christian S Merz C Evans L Gradl S Seidel H Friberg A. The novel dihydroorotate dehydrogenase (DHODH) inhibitor BAY 2402234 triggers differentiation and is effective in the treatment of myeloid malignancies. Leukemia (2019) 33(10):2403–15. doi: 10.1038/s41375-019-0461-5 30940908

[204] Fang H Liu H Chen N Zhang C Xie X Xu Q. Site-directed mutagenesis studies on the uridine monophosphate binding sites of feedback inhibition in carbamoyl phosphate synthetase and effects on cytidine production by bacillus amyloliquefaciens. Can J Microbiol (2013) 59(6):374–9. doi: 10.1139/cjm-2012-0758 23750951

[205] Gorrell A Wang W Underbakke E Hou Z Honzatko RB Fromm HJ. Determinants of l-aspartate and IMP recognition in escherichia coli adenylosuccinate synthetase. J Biol Chem (2002) 277(11):8817–21. doi: 10.1074/jbc.M111810200 11781326

[206] Sarwono AEY Mitsuhashi S Kabir MHB Shigetomi K Okada T Ohsaka F. Repurposing existing drugs: identification of irreversible IMPDH inhibitors by high-throughput screening. J Enzyme Inhib Med Chem (2019) 34(1):171–8. doi: 10.1080/14756366.2018.1540474 PMC6249553 30451014

[207] Tang W Li X Zhu Z Tong S Li X Zhang X. Expression, purification, crystallization and preliminary X-ray diffraction analysis of human phosphoribosyl pyrophosphate synthetase 1 (PRS1). Acta Crystallogr Sect F Struct Biol Cryst Commun (2006) 62(Pt 5):432–4. doi: 10.1107/s1744309106009067 PMC2219982 16682768

[208] Yamaoka T Itakura M. [Metabolism of purine nucleotides and the production of uric acid]. Nihon Rinsho (1996) 54(12):3188–94. 8976090

[209] Aird KM Zhang R. Nucleotide metabolism, oncogene-induced senescence and cancer. Cancer Lett (2015). doi: 10.1016/j.canlet.2014.01.017 PMC4115046 24486217

[210] Eberhardy SR Farnham PJ. C-myc mediates activation of the cad promoter *via* a post-RNA polymerase II recruitment mechanism. J Biol Chem (2001) 276(51):48562–71. doi: 10.1074/jbc.M109014200 11673469

[211] Liu YC Li F Handler J Huang CR Xiang Y Neretti N. Global regulation of nucleotide biosynthetic genes by c-myc. PloS One (2008) 3(7):e2722. doi: 10.1371/journal.pone.0002722 18628958 PMC2444028

[212] Mannava S Grachtchouk V Wheeler LJ Im M Zhuang D Slavina EG. Direct role of nucleotide metabolism in c-MYC-dependent proliferation of melanoma cells. Cell Cycle (2008) 7(15):2392–400. doi: 10.4161/cc.6390 PMC3744895 18677108

[213] Ben-Sahra I Hoxhaj G Ricoult SJH Asara JM Manning BD. mTORC1 induces purine synthesis through control of the mitochondrial tetrahydrofolate cycle. Science (2016) 351(6274):728–33. doi: 10.1126/science.aad0489 PMC4786372 26912861

[214] Real S Meo-Evoli N Espada L Tauler A. E2F1 regulates cellular growth by mTORC1 signaling. PloS One (2011) 6(1):e16163. doi: 10.1371/journal.pone.0016163 21283628 PMC3026008

[215] Ben-Sahra I Howell JJ Asara JM Manning BD. Stimulation of *de novo* pyrimidine synthesis by growth signaling through mTOR and S6K1. Science (2013) 339(6125):1323–8. doi: 10.1126/science.1228792 PMC3753690 23429703

[216] Robitaille AM Christen S Shimobayashi M Cornu M Fava LL Moes S. Quantitative phosphoproteomics reveal mTORC1 activates *de novo* pyrimidine synthesis. Science (2013) 339(6125):1320–3. doi: 10.1126/science.1228771 23429704

[217] Santana-Codina N Roeth AA Zhang Y Yang A Mashadova O Asara JM. Oncogenic KRAS supports pancreatic cancer through regulation of nucleotide synthesis. Nat Commun (2018) 9(1):4945. doi: 10.1038/s41467-018-07472-8 30470748 PMC6251888

[218] Wang H Wang X Xu L Zhang J Cao H. High expression levels of pyrimidine metabolic rate-limiting enzymes are adverse prognostic factors in lung adenocarcinoma: a study based on the cancer genome atlas and gene expression omnibus datasets. Purinergic Signal (2020) 16(3):347–66. doi: 10.1007/s11302-020-09711-4 PMC7524999 32638267

[219] Yu Y Ding J Zhu S Alptekin A Dong Z Yan C. Therapeutic targeting of both dihydroorotate dehydrogenase and nucleoside transport in MYCN-amplified neuroblastoma. Cell Death Dis (2021) 12(9):821. doi: 10.1038/s41419-021-04120-w 34462431 PMC8405683

[220] Kollareddy M Dimitrova E Vallabhaneni KC Chan A Le T Chauhan KM. Regulation of nucleotide metabolism by mutant p53 contributes to its gain-of-function activities. Nat Commun (2015) 6:7389. doi: 10.1038/ncomms8389 26067754 PMC4467467

[221] Kreuzaler P Panina Y Segal J Yuneva M. Adapt and conquer: Metabolic flexibility in cancer growth, invasion and evasion. Mol Metab (2020) 33:83–101. doi: 10.1016/j.molmet.2019.08.021 31668988 PMC7056924

[222] Abbaszadeh Z Çeşmeli S Biray Avcı Ç.. Crucial players in glycolysis: Cancer progress. Gene (2020) 726:144158. doi: 10.1016/j.gene.2019.144158 31629815

[223] Moldogazieva NT Mokhosoev IM Terentiev AA. Metabolic heterogeneity of cancer cells: An interplay between HIF-1, GLUTs, and AMPK. Cancers (Basel) (2020) 12(4):862. doi: 10.3390/cancers12040862 32252351 PMC7226606

[224] Zhou CH Zhang XP Liu F Wang W. Modeling the interplay between the HIF-1 and p53 pathways in hypoxia. Sci Rep (2015) 5:13834. doi: 10.1038/srep13834 26346319 PMC4561886

[225] Bouchez CL Hammad N Cuvellier S Ransac S Rigoulet M Devin A. The warburg effect in yeast: Repression of mitochondrial metabolism is not a prerequisite to promote cell proliferation. Front Oncol (2020) 10:1333. doi: 10.3389/fonc.2020.01333 32974131 PMC7466722

[226] Warburg O. On the origin of cancer cells. Science (1956) 123(3191):309–14. doi: 10.1126/science.123.3191.309 13298683

[227] Takeuchi Y Nakayama Y Fukusaki E Irino Y. Glutamate production from ammonia *via* glutamate dehydrogenase 2 activity supports cancer cell proliferation under glutamine depletion. Biochem Biophys Res Commun (2018) 495(1):761–7. doi: 10.1016/j.bbrc.2017.11.088 29146184

[228] Wise DR DeBerardinis RJ Mancuso A Sayed N Zhang XY Pfeiffer HK. Myc regulates a transcriptional program that stimulates mitochondrial glutaminolysis and leads to glutamine addiction. Proc Natl Acad Sci U.S.A. (2008) 105(48):18782–7. doi: 10.1073/pnas.0810199105 PMC2596212 19033189

[229] Alfarouk KO Ahmed SBM Elliott RL Benoit A Alqahtani SS Ibrahim ME. The pentose phosphate pathway dynamics in cancer and its dependency on intracellular pH. Metabolites (2020) 10(7):777. doi: 10.3390/metabo10070285 PMC7407102 32664469

[230] Jiang P Du W Wu M. Regulation of the pentose phosphate pathway in cancer. Protein Cell (2014) 5(8):592–602. doi: 10.1007/s13238-014-0082-8 25015087 PMC4112277

[231] Shuvalov O Petukhov A Daks A Fedorova O Vasileva E Barlev NA. One-carbon metabolism and nucleotide biosynthesis as attractive targets for anticancer therapy. Oncotarget (2017) 8(14):23955–77. doi: 10.18632/oncotarget.15053 PMC5410357 28177894

[232] Koundouros N Poulogiannis G. Reprogramming of fatty acid metabolism in cancer. Br J Cancer (2020) 122(1):4–22. doi: 10.1038/s41416-019-0650-z 31819192 PMC6964678

[233] Maan M Peters JM Dutta M Patterson AD. Lipid metabolism and lipophagy in cancer. Biochem Biophys Res Commun (2018) 504(3):582–9. doi: 10.1016/j.bbrc.2018.02.097 PMC6086774 29438712

[234] Santos CR Schulze A. Lipid metabolism in cancer. FEBS J (2012) 279(15):2610–23. doi: 10.1111/j.1742-4658.2012.08644.x 22621751

[235] Misale S Yaeger R Hobor S Scala E Janakiraman M Liska D. Emergence of KRAS mutations and acquired resistance to anti-EGFR therapy in colorectal cancer. Nature (2012) 486(7404):532–6. doi: 10.1038/nature11156 PMC3927413 22722830

[236] Morrell JA Orme J Butlin RJ Roche TE Mayers RM Kilgour E. AZD7545 is a selective inhibitor of pyruvate dehydrogenase kinase 2. Biochem Soc Trans (2003) 31(Pt 6):1168–70. doi: 10.1042/bst0311168 14641019

[237] Le A Cooper CR Gouw AM Dinavahi R Maitra A Deck LM. Inhibition of lactate dehydrogenase a induces oxidative stress and inhibits tumor progression. Proc Natl Acad Sci U.S.A. (2010) 107(5):2037–42. doi: 10.1073/pnas.0914433107 PMC2836706 20133848

[238] Chang L Fang S Gu W. The molecular mechanism of metabolic remodeling in lung cancer. J Cancer (2020) 11(6):1403–11. doi: 10.7150/jca.31406 PMC6995370 32047547

[239] Luo D Shan Z Liu Q Cai S Li Q Li X. A novel seventeen-gene metabolic signature for predicting prognosis in colon cancer. BioMed Res Int (2020) 2020:12. doi: 10.1155/2020/4845360 PMC7685801 33282950

[240] Qiu Y Cai G Zhou B Li D Zhao A Xie G. A distinct metabolic signature of human colorectal cancer with prognostic potential. Clin Cancer Res (2014) 20(8):2136–46. doi: 10.1158/1078-0432.CCR-13-1939 PMC5902798 24526730

[241] Zhong DS Xiong L Liu TR Liu XJ Liu XG Chen J. The glycolytic inhibitor 2-deoxyglucose activates multiple prosurvival pathways through IGF1R. J Biol Chem (2009) 284(35):23225–33. doi: 10.1074/jbc.M109.005280 PMC2749096 19574224

[242] Colangelo T Polcaro G Muccillo L D'Agostino G Rosato V Ziccardi P. Friend or foe? the tumour microenvironment dilemma in colorectal cancer. Biochim Biophys Acta Rev Cancer (2017) 1867(1):1–18. doi: 10.1016/j.bbcan.2016.11.001 27864070

[243] Fang T Lv H Lv G Li T Wang C Han Q. Tumor-derived exosomal miR-1247-3p induces cancer-associated fibroblast activation to foster lung metastasis of liver cancer. Nat Commun (2018) 9(1):191. doi: 10.1038/s41467-017-02583-0 29335551 PMC5768693

[244] Costa A Kieffer Y Scholer-Dahirel A Pelon F Bourachot B Cardon M. Fibroblast heterogeneity and immunosuppressive environment in human breast cancer. Cancer Cell (2018) 33(3):463–79.e10. doi: 10.1016/j.ccell.2018.-01.011 29455927

[245] Özdemir BC Pentcheva-Hoang T Carstens JL Zheng X Wu CC Simpson TR. Depletion of carcinoma-associated fibroblasts and fibrosis induces immunosuppression and accelerates pancreas cancer with reduced survival. Cancer Cell (2014) 25(6):719–34. doi: 10.1016/j.ccr.2014.04.005 PMC4180632 24856586

[246] Bertolini G D'Amico L Moro M Landoni E Perego P Miceli R. Microenvironment-modulated metastatic CD133+/CXCR4+/EpCAM- lung cancer-initiating cells sustain tumor dissemination and correlate with poor prognosis. Cancer Res (2015) 75(17):3636–49. doi: 10.1158/0008-5472.Can-14-3781 26141860

[247] Peiris-Pagès M Sotgia F Lisanti MP. Chemotherapy induces the cancer-associated fibroblast phenotype, activating paracrine hedgehog-GLI signalling in breast cancer cells. Oncotarget (2015) 6(13):10728–45. doi: 10.18632/oncotarget.3828 PMC4484415 25915429

[248] Gorchs L Hellevik T Bruun JA Camilio KA Al-Saad S Stuge TB. Cancer-associated fibroblasts from lung tumors maintain their immunosuppressive abilities after high-dose irradiation. Front Oncol (2015) 5:87. doi: 10.3389/fonc.2015.00087 26029659 PMC4429237

[249] Becker LM O'Connell JT Vo AP Cain MP Tampe D Bizarro L. Epigenetic reprogramming of cancer-associated fibroblasts deregulates glucose metabolism and facilitates progression of breast cancer. Cell Rep (2020) 31(9):107701. doi: 10.1016/j.celrep.2020.107701 32492417 PMC7339325

[250] Wen YA Xing X Harris JW Zaytseva YY Mitov MI Napier DL. Adipocytes activate mitochondrial fatty acid oxidation and autophagy to promote tumor growth in colon cancer. Cell Death Dis (2017) 8(2):e2593. doi: 10.1038/cddis.2017.21 28151470 PMC5386470

[251] Pavlides S Whitaker-Menezes D Castello-Cros R Flomenberg N Witkiewicz AK Frank PG. The reverse warburg effect: aerobic glycolysis in cancer associated fibroblasts and the tumor stroma. Cell Cycle (2009) 8(23):3984–4001. doi: 10.4161/cc.8.23.10238 19923890

[252] Fu Y Liu S Yin S Niu W Xiong W Tan M. The reverse warburg effect is likely to be an achilles' heel of cancer that can be exploited for cancer therapy. Oncotarget (2017) 8(34):57813–25. doi: 10.18632/oncotarget.18175 PMC5593685 28915713

[253] Wilde L Roche M Domingo-Vidal M Tanson K Philp N Curry J. Metabolic coupling and the reverse warburg effect in cancer: Implications for novel biomarker and anticancer agent development. Semin Oncol (2017) 44(3):198–203. doi: 10.1053/j.seminoncol.2017.10.004 29248131 PMC5737780

[254] Seyfried TN. Cancer as a mitochondrial metabolic disease. Front Cell Dev Biol (2015) 3:43. doi: 10.3389/fcell.2015.00043 26217661 PMC4493566

[255] Sonveaux P Végran F Schroeder T Wergin MC Verrax J Rabbani ZN. Targeting lactate-fueled respiration selectively kills hypoxic tumor cells in mice. J Clin Invest (2008) 118(12):3930–42. doi: 10.1172/jci36843 PMC2582933 19033663

[256] Pisarsky L Bill R Fagiani E Dimeloe S Goosen RW Hagmann J. Targeting metabolic symbiosis to overcome resistance to anti-angiogenic therapy. Cell Rep (2016) 15(6):1161–74. doi: 10.1016/j.celrep.2016.04.028 PMC4870473 27134168

[257] Bar EE Lin A Mahairaki V Matsui W Eberhart CG. Hypoxia increases the expression of stem-cell markers and promotes clonogenicity in glioblastoma neurospheres. Am J Pathol (2010) 177(3):1491–502. doi: 10.2353/ajpath.2010.091021 PMC2928980 20671264

[258] Pistollato F Chen HL Schwartz PH Basso G Panchision DM. Oxygen tension controls the expansion of human CNS precursors and the generation of astrocytes and oligodendrocytes. Mol Cell Neurosci (2007) 35(3):424–35. doi: 10.1016/j.mcn.2007.04.003 17498968

[259] Semenza GL. HIF-1: upstream and downstream of cancer metabolism. Curr Opin Genet Dev (2010) 20(1):51–6. doi: 10.1016/j.gde.2009.10.009 PMC2822127 19942427

[260] Reina-Campos M Moscat J Diaz-Meco M. Metabolism shapes the tumor microenvironment. Curr Opin Cell Biol (2017) 48:47–53. doi: 10.1016/j.ceb.2017.05.006 28605656 PMC5650101

[261] Hirata E Girotti MR Viros A Hooper S Spencer-Dene B Matsuda M. Intravital imaging reveals how BRAF inhibition generates drug-tolerant microenvironments with high integrin β1/FAK signaling. Cancer Cell (2015) 27(4):574–88. doi: 10.1016/j.ccell.2015.03.008 PMC4402404 25873177

[262] Qin J Li R Raes J Arumugam M Burgdorf KS Manichanh C. A human gut microbial gene catalogue established by metagenomic sequencing. Nature (2010) 464(7285):59–65. doi: 10.1038/nature08821 20203603 PMC3779803

[263] Keku TO Dulal S Deveaux A Jovov B Han X. The gastrointestinal microbiota and colorectal cancer. Am J Physiol Gastrointest Liver Physiol (2015) 308(5):G351–63. doi: 10.1152/ajpgi.00360.2012 PMC4346754 25540232

[264] Mittal S Brown NJ Holen I. The breast tumor microenvironment: role in cancer development, progression and response to therapy. Expert Rev Mol Diagn (2018) 18(3):227–43. doi: 10.1080/14737159.2018.1439382 29424261

[265] Liu D Chang C Lu N Wang X Lu Q Ren X. Comprehensive proteomics analysis reveals metabolic reprogramming of tumor-associated macrophages stimulated by the tumor microenvironment. J Proteome Res (2017) 16(1):288–97. doi: 10.1021/acs.jproteome.6b00604 27809537

[266] Lee DC Sohn HA Park ZY Oh S Kang YK Lee KM. A lactate-induced response to hypoxia. Cell (2015) 161(3):595–609. doi: 10.1016/j.cell.2015.03.011 25892225

[267] Lin S Sun L Lyu X Ai X Du D Su N. Lactate-activated macrophages induced aerobic glycolysis and epithelial-mesenchymal transition in breast cancer by regulation of CCL5-CCR5 axis: a positive metabolic feedback loop. Oncotarget (2017) 8(66):110426–43. doi: 10.18632/oncotarget.22786 PMC5746394 29299159

[268] Miricescu D Totan A Stanescu S II Badoiu SC Stefani C Greabu M. PI3K/AKT/mTOR signaling pathway in breast cancer: From molecular landscape to clinical aspects. Int J Mol Sci (2020) 22(1):173. doi: 10.3390/ijms22010173 33375317 PMC7796017

[269] Wenes M Shang M Di Matteo M Goveia J Martín-Pérez R Serneels J. Macrophage metabolism controls tumor blood vessel morphogenesis and metastasis. Cell Metab (2016) 24(5):701–15. doi: 10.1016/j.cmet.2016.09.008 27773694

[270] Hollmén M Roudnicky F Karaman S Detmar M. Characterization of macrophage–cancer cell crosstalk in estrogen receptor positive and triple-negative breast cancer. Sci Rep (2015) 5:9188. doi: 10.1038/srep09188 25776849 PMC4361875

[271] Rath M Müller I Kropf P Closs EI Munder M. Metabolism *via* arginase or nitric oxide synthase: Two competing arginine pathways in macrophages. Front Immunol (2014) 5:532. doi: 10.3389/fimmu.2014.00532 25386178 PMC4209874

[272] Bantug GR Galluzzi L Kroemer G Hess C. The spectrum of T cell metabolism in health and disease. Nat Rev Immunol (2018) 18(1):19–34. doi: 10.1038/nri.2017.99 28944771

[273] Brand A Singer K Koehl GE Kolitzus M Schoenhammer G Thiel A. LDHA-associated lactic acid production blunts tumor immunosurveillance by T and NK cells. Cell Metab (2016) 24(5):657–71. doi: 10.1016/j.cmet.2016.08.011 27641098

[274] Jian SL Chen WW Su YC Su YW Chuang TH Hsu SC. Glycolysis regulates the expansion of myeloid-derived suppressor cells in tumor-bearing hosts through prevention of ROS-mediated apoptosis. Cell Death Dis (2017) 8(5):e2779. doi: 10.1038/cddis.2017.192 28492541 PMC5520713

[275] Boutté AM McDonald WH Shyr Y Yang L Lin PC. Characterization of the MDSC proteome associated with metastatic murine mammary tumors using label-free mass spectrometry and shotgun proteomics. PloS One (2011) 6(8):e22446. doi: 10.1371/journal.pone.0022446 21853032 PMC3154190

[276] Altorki NK Markowitz GJ Gao D Port JL Saxena A Stiles B. The lung microenvironment: an important regulator of tumour growth and metastasis. Nat Rev Cancer (2019) 19(1):9–31. doi: 10.1038/s41568-018-0081-9 30532012 PMC6749995

[277] Schmall A Al-Tamari HM Herold S Kampschulte M Weigert A Wietelmann A. Macrophage and cancer cell cross-talk *via* CCR2 and CX3CR1 is a fundamental mechanism driving lung cancer. Am J Respir Crit Care Med (2015) 191(4):437–47. doi: 10.1164/rccm.201406-1137OC 25536148

[278] Lewis ND Asim M Barry DP de Sablet T Singh K Piazuelo MB. Immune evasion by helicobacter pylori is mediated by induction of macrophage arginase II. J Immunol (2011) 186(6):3632–41. doi: 10.4049/jimmunol.1003431 PMC3069806 21296975

[279] Rodriguez PC Quiceno DG Zabaleta J Ortiz B Zea AH Piazuelo MB. Arginase I production in the tumor microenvironment by mature myeloid cells inhibits T-cell receptor expression and antigen-specific T-cell responses. Cancer Res (2004) 64(16):5839–49. doi: 10.1158/0008-5472.Can-04-0465 15313928

[280] Zhao F Xiao C Evans KS Theivanthiran T DeVito N Holtzhausen A. Paracrine Wnt5a-β-Catenin signaling triggers a metabolic program that drives dendritic cell tolerization. Immunity (2018) 48(1):147–60.e7. doi: 10.1016/j.immuni.2017.12.004 29343435 PMC5777287

[281] Leone RD Zhao L Englert JM Sun IM Oh MH Sun IH. Glutamine blockade induces divergent metabolic programs to overcome tumor immune evasion. Science (2019) 366(6468):1013–21. doi: 10.1126/science.aav2588 PMC7023461 31699883

[282] Sprinzl MF Reisinger F Puschnik A Ringelhan M Ackermann K Hartmann D. Sorafenib perpetuates cellular anticancer effector functions by modulating the crosstalk between macrophages and natural killer cells. Hepatology (2013) 57(6):2358–68. doi: 10.1002/hep.26328 23424039

[283] Zhang Q Wang H Mao C Sun M Dominah G Chen L. Fatty acid oxidation contributes to IL-1β secretion in M2 macrophages and promotes macrophage-mediated tumor cell migration. Mol Immunol (2018) 94:27–35. doi: 10.1016/j.molimm.2017.12.011 29248877 PMC5801116

[284] Borregaard N Herlin T. Energy metabolism of human neutrophils during phagocytosis. J Clin Invest (1982) 70(3):550–7. doi: 10.1172/jci110647 PMC370256 7107894

[285] Fossati G Moulding DA Spiller DG Moots RJ White MR Edwards SW. The mitochondrial network of human neutrophils: role in chemotaxis, phagocytosis, respiratory burst activation, and commitment to apoptosis. J Immunol (2003) 170(4):1964–72. doi: 10.4049/jimmunol.170.4.1964 12574365

[286] Rice CM Davies LC Subleski JJ Maio N Gonzalez-Cotto M Andrews C. Tumour-elicited neutrophils engage mitochondrial metabolism to circumvent nutrient limitations and maintain immune suppression. Nat Commun (2018) 9(1):5099. doi: 10.1038/s41467-018-07505-2 30504842 PMC6269473

[287] Macintyre AN Gerriets VA Nichols AG Michalek RD Rudolph MC Deoliveira D. The glucose transporter Glut1 is selectively essential for CD4 T cell activation and effector function. Cell Metab (2014) 20(1):61–72. doi: 10.1016/j.cmet.2014.05.004 24930970 PMC4079750

[288] Michalek RD Gerriets VA Jacobs SR Macintyre AN MacIver NJ Mason EF. Cutting edge: distinct glycolytic and lipid oxidative metabolic programs are essential for effector and regulatory CD4+ T cell subsets. J Immunol (2011) 186(6):3299–303. doi: 10.4049/jimmunol.1003613 PMC3198034 21317389

[289] Shi LZ Wang R Huang G Vogel P Neale G Green DR. HIF1alpha-dependent glycolytic pathway orchestrates a metabolic checkpoint for the differentiation of TH17 and treg cells. J Exp Med (2011) 208(7):1367–76. doi: 10.1084/jem.20110278 PMC3135370 21708926

[290] Zeng H Chi H. mTOR signaling in the differentiation and function of regulatory and effector T cells. Curr Opin Immunol (2017) 46:103–11. doi: 10.1016/j.coi.2017.04.005 PMC5554750 28535458

[291] Dey P Kundu A Sachan R Park JH Ahn MY Yoon K. PKM2 knockdown induces autophagic cell death *via* AKT/mTOR pathway in human prostate cancer cells. Cell Physiol Biochem (2019) 52(6):1535–52. doi: 10.33594/000000107 31135122

[292] Dias AS Almeida CR Helguero LA Duarte IF. Metabolic crosstalk in the breast cancer microenvironment. Eur J Cancer (2019) 121:154–71. doi: 10.1016/j.ejca.2019.09.002 31581056

[293] Hambardzumyan D Gutmann DH Kettenmann H. The role of microglia and macrophages in glioma maintenance and progression. Nat Neurosci (2016) 19(1):20–7. doi: 10.1038/nn.4185 PMC4876023 26713745

[294] Martinez-Outschoorn U Sotgia F Lisanti MP. Tumor microenvironment and metabolic synergy in breast cancers: critical importance of mitochondrial fuels and function. Semin Oncol (2014) 41(2):195–216. doi: 10.1053/j.seminoncol.2014.03.002 24787293

[295] Quail DF Joyce JA. The microenvironmental landscape of brain tumors. Cancer Cell (2017) 31(3):326–41. doi: 10.1016/j.ccell.2017.02.009 PMC5424263 28292436

[296] Sevic I Spinelli FM Cantero MJ Reszegi A Kovalszky I García MG. The role of the tumor microenvironment in the development and progression of hepatocellular carcinoma. Hepatocellular Carcinoma (2019). 31664802

[297] Wroblewski LE Peek RM Jr.. Helicobacter pylori in gastric carcinogenesis: mechanisms. Gastroenterol Clin North Am (2013) 42(2):285–98. doi: 10.1016/j.gtc.2013.01.006 PMC3648881 23639641

[298] Xia Y Brown ZJ Huang H Tsung A. Metabolic reprogramming of immune cells: Shaping the tumor microenvironment in hepatocellular carcinoma. Cancer Med (2021) 10(18):6374–83. doi: 10.1002/cam4.4177 PMC8446566 34390203

[299] Zhao L Liu Y Zhang S Wei L Cheng H Wang J. Impacts and mechanisms of metabolic reprogramming of tumor microenvironment for immunotherapy in gastric cancer. Cell Death Dis (2022) 13(4):378. doi: 10.1038/s41419-022-04821-w 35444235 PMC9021207

[300] Zheng X Mansouri S Krager A Grimminger F Seeger W Pullamsetti SS. Metabolism in tumour-associated macrophages: a quid pro quo with the tumour microenvironment. Eur Respir Rev (2020) 29(157):200134. doi: 10.1183/16000617.0134-2020 33004525 PMC9488699

[301] Zhou W Wahl DR. Metabolic abnormalities in glioblastoma and metabolic strategies to overcome treatment resistance. Cancers (Basel) (2019) 11(9):1231. doi: 10.3390/cancers11091231 31450721 PMC6770393

[302] Raez LE Papadopoulos K Ricart AD Chiorean EG Dipaola RS Stein MN. A phase I dose-escalation trial of 2-deoxy-D-glucose alone or combined with docetaxel in patients with advanced solid tumors. Cancer Chemother Pharmacol (2013) 71(2):523–30. doi: 10.1007/s00280-012-2045-1 23228990

[303] Stein M Lin H Jeyamohan C Dvorzhinski D Gounder M Bray K. Targeting tumor metabolism with 2-deoxyglucose in patients with castrate-resistant prostate cancer and advanced malignancies. Prostate (2010) 70(13):1388–94. doi: 10.1002/pros.21172 PMC4142700 20687211

[304] Zhang D Li J Wang F Hu J Wang S Sun Y. 2-Deoxy-D-glucose targeting of glucose metabolism in cancer cells as a potential therapy. Cancer Lett (2014) 355(2):176–83. doi: 10.1016/j.canlet.2014.09.003 25218591

[305] Luengo A Gui DY Vander Heiden MG. Targeting metabolism for cancer therapy. Cell Chem Biol (2017) 24(9):1161–80. doi: 10.1016/j.chembiol.2017.08.028 PMC5744685 28938091

[306] Ralser M Wamelink MM Struys EA Joppich C Krobitsch S Jakobs C. A catabolic block does not sufficiently explain how 2-deoxy-D-glucose inhibits cell growth. Proc Natl Acad Sci U.S.A. (2008) 105(46):17807–11. doi: 10.1073/pnas.0803090105 PMC2584745 19004802

[307] Fan T Sun G Sun X Zhao L Zhong R Peng Y. Tumor energy metabolism and potential of 3-bromopyruvate as an inhibitor of aerobic glycolysis: Implications in tumor treatment. Cancers (Basel) (2019) 11(3):317. doi: 10.3390/cancers11030317 30845728 PMC6468516

[308] Huang Y Sun G Sun X Li F Zhao L Zhong R. The potential of lonidamine in combination with chemotherapy and physical therapy in cancer treatment. Cancers (Basel) (2020) 12(11):3332. doi: 10.3390/cancers12113332 33187214 PMC7696079

[309] Gatzemeier U Cavalli F Haussinger K Kaukel E Koschel G Martinelli G. Phase III trial with and without lonidamine in non-small cell lung cancer. Semin Oncol (1991) 18(2 Suppl 4):42–8. 1851577

[310] Xiang L Mou J Shao B Wei Y Liang H Takano N. Glutaminase 1 expression in colorectal cancer cells is induced by hypoxia and required for tumor growth, invasion, and metastatic colonization. Cell Death Dis (2019) 10(2):40. doi: 10.1038/s41419-018-1291-5 30674873 PMC6426853

[311] Vora S Halper JP Knowles DM. Alterations in the activity and isozymic profile of human phosphofructokinase during malignant transformation *in vivo* and *in vitro*: transformation- and progression-linked discriminants of malignancy. Cancer Res (1985) 45(7):2993–3001. 3159473

[312] Wang X Gorospe M Huang Y Holbrook NJ. p27Kip1 overexpression causes apoptotic death of mammalian cells. Oncogene (1997) 15(24):2991–7. doi: 10.1038/sj.onc.1201450 9416843

[313] Yalcin A Clem BF Imbert-Fernandez Y Ozcan SC Peker S O'Neal J. 6-Phosphofructo-2-kinase (PFKFB3) promotes cell cycle progression and suppresses apoptosis *via* Cdk1-mediated phosphorylation of p27. Cell Death Dis (2014) 5(7):e1337. doi: 10.1038/cddis.2014.292 25032860 PMC4123086

[314] Shi L Pan H Liu Z Xie J Han W. Roles of PFKFB3 in cancer. Signal Transduct Target Ther (2017) 2:17044. doi: 10.1038/sigtrans.2017.44 29263928 PMC5701083

[315] Zhu W Ye L Zhang J Yu P Wang H Ye Z. PFK15, a small molecule inhibitor of PFKFB3, induces cell cycle arrest, apoptosis and inhibits invasion in gastric cancer. PloS One (2016) 11(9):e0163768. doi: 10.1371/journal.pone.0163768 27669567 PMC5036843

[316] Kapoor K Finer-Moore JS Pedersen BP Caboni L Waight A Hillig RC. Mechanism of inhibition of human glucose transporter GLUT1 is conserved between cytochalasin b and phenylalanine amides. Proc Natl Acad Sci U.S.A. (2016) 113(17):4711–6. doi: 10.1073/pnas.1603735113 PMC4855560 27078104

[317] Rastogi S Banerjee S Chellappan S Simon GR. Glut-1 antibodies induce growth arrest and apoptosis in human cancer cell lines. Cancer Lett (2007) 257(2):244–51. doi: 10.1016/j.canlet.2007.07.021 17910902

[318] Bola BM Chadwick AL Michopoulos F Blount KG Telfer BA Williams KJ. Inhibition of monocarboxylate transporter-1 (MCT1) by AZD3965 enhances radiosensitivity by reducing lactate transport. Mol Cancer Ther (2014) 13(12):2805–16. doi: 10.1158/1535-7163.Mct-13-1091 PMC4258406 25281618

[319] Payen VL Mina E Van Hée VF Porporato PE Sonveaux P. Monocarboxylate transporters in cancer. Mol Metab (2020) 33:48–66. doi: 10.1016/j.molmet.2019.07.006 31395464 PMC7056923

[320] Kowalik MA Columbano A Perra A. Emerging role of the pentose phosphate pathway in hepatocellular carcinoma. Front Oncol (2017) 7:87. doi: 10.3389/fonc.2017.00087 28553614 PMC5425478

[321] Lonardo E Cioffi M Sancho P Sanchez-Ripoll Y Trabulo SM Dorado J. Metformin targets the metabolic achilles heel of human pancreatic cancer stem cells. PloS One (2013) 8(10):e76518. doi: 10.1371/journal.pone.0076518 24204632 PMC3799760

[322] Dong L Neuzil J. Targeting mitochondria as an anticancer strategy. Cancer Commun (Lond) (2019) 39(1):63. doi: 10.1186/s40880-019-0412-6 31653274 PMC6815053

[323] Michelakis ED Sutendra G Dromparis P Webster L Haromy A Niven E. Metabolic modulation of glioblastoma with dichloroacetate. Sci Transl Med (2010) 2(31):31ra4. doi: 10.1126/scitranslmed.3000677 20463368

[324] Mele L Paino F Papaccio F Regad T Boocock D Stiuso P. A new inhibitor of glucose-6-phosphate dehydrogenase blocks pentose phosphate pathway and suppresses malignant proliferation and metastasis in vivo. Cell Death Dis (2018) 9(5):572. doi: 10.1038/s41419-018-0635-5 29760380 PMC5951921

[325] Cai H Scott E Kholghi A Andreadi C Rufini A Karmokar A. Cancer chemoprevention: Evidence of a nonlinear dose response for the protective effects of resveratrol in humans and mice. Sci Transl Med (2015) 7(298):298ra117. doi: 10.1126/scitranslmed.aaa7619 PMC4827609 26223300

[326] Köhler E Barrach H Neubert D. Inhibition of NADP dependent oxidoreductases by the 6-aminonicotinamide analogue of NADP. FEBS Lett (1970) 6(3):225–8. doi: 10.1016/0014-5793(70)80063-1 11947380

[327] Liu H Zhao S Zhang Y Wu J Peng H Fan J. Reactive oxygen species-mediated endoplasmic reticulum stress and mitochondrial dysfunction contribute to polydatin-induced apoptosis in human nasopharyngeal carcinoma CNE cells. J Cell Biochem (2011) 112(12):3695–703. doi: 10.1002/jcb.23303 21815196

[328] Herter FP Weissman SG Thompson HG Jr. Hyman G Martin DS. Clinical experience with 6-aminonicotinamide. Cancer Res (1961) 21(1):31–7. 13713803

[329] Lukey MJ Wilson KF Cerione RA. Therapeutic strategies impacting cancer cell glutamine metabolism. Future Med Chem (2013) 5(14):1685–700. doi: 10.4155/fmc.13.130 PMC4154374 24047273

[330] Robinson MM McBryant SJ Tsukamoto T Rojas C Ferraris DV Hamilton SK. Novel mechanism of inhibition of rat kidney-type glutaminase by bis-2-(5-phenylacetamido-1,2,4-thiadiazol-2-yl)ethyl sulfide (BPTES). (2007) 406(3):407–14. doi: 10.1042/bj20070039 PMC2049044 17581113

[331] Shukla K Ferraris DV Thomas AG Stathis M Duvall B Delahanty G. Design, synthesis, and pharmacological evaluation of bis-2-(5-phenylacetamido-1,2,4-thiadiazol-2-yl)ethyl sulfide 3 (BPTES) analogs as glutaminase inhibitors. J Med Chem (2012) 55(23):10551–63. doi: 10.1021/jm301191p PMC3539823 23151085

[332] Akins NS Nielson TC Le HV. Inhibition of glycolysis and glutaminolysis: An emerging drug discovery approach to combat cancer. Curr Top Med Chem (2018) 18(6):494–504. doi: 10.2174/1568026618666180523111351 29788892 PMC6110043

[333] Grigoryan RS Panosyan EH Seibel NL Gaynon PS Avramis IA Avramis VI. Changes of amino acid serum levels in pediatric patients with higher-risk acute lymphoblastic leukemia (CCG-1961). In Vivo (2004) 18(2):107–12. 15113036

[334] Dolfi SC Chan LL Qiu J Tedeschi PM Bertino JR Hirshfield KM. The metabolic demands of cancer cells are coupled to their size and protein synthesis rates. Cancer Metab (2013) 1(1):20. doi: 10.1186/2049-3002-1-20 24279929 PMC4178206

[335] Jain M Nilsson R Sharma S Madhusudhan N Kitami T Souza AL. Metabolite profiling identifies a key role for glycine in rapid cancer cell proliferation. Science (2012) 336(6084):1040–4. doi: 10.1126/science.1218595 PMC3526189 22628656

[336] Mattaini KR Sullivan MR Vander Heiden MG. The importance of serine metabolism in cancer. J Cell Biol (2016) 214(3):249–57. doi: 10.1083/jcb.201604085 PMC4970329 27458133

[337] Qu Q Zeng F Liu X Wang QJ Deng F. Fatty acid oxidation and carnitine palmitoyltransferase I: emerging therapeutic targets in cancer. Cell Death Dis (2016) 7(5):e2226. doi: 10.1038/cddis.2016.132 27195673 PMC4917665

[338] Cheng S Wang G Wang Y Cai L Qian K Ju L. Fatty acid oxidation inhibitor etomoxir suppresses tumor progression and induces cell cycle arrest *via* PPARγ-mediated pathway in bladder cancer. Clin Sci (Lond) (2019) 133(15):1745–58. doi: 10.1042/cs20190587 31358595

[339] Flaig TW Salzmann-Sullivan M Su LJ Zhang Z Joshi M Gijón MA. Lipid catabolism inhibition sensitizes prostate cancer cells to antiandrogen blockade. Oncotarget (2017) 8(34):56051–65. doi: 10.18632/oncotarget.17359 PMC5593544 28915573

[340] Gugiatti E Tenca C Ravera S Fabbi M Ghiotto F Mazzarello AN. A reversible carnitine palmitoyltransferase (CPT1) inhibitor offsets the proliferation of chronic lymphocytic leukemia cells. Haematologica (2018) 103(11):e531–e6. doi: 10.3324/haematol.2017.175414 PMC6278982 29930162

[341] O'Connor RS Guo L Ghassemi S Snyder NW Worth AJ Weng L. The CPT1a inhibitor, etomoxir induces severe oxidative stress at commonly used concentrations. Sci Rep (2018) 8(1):6289. doi: 10.1038/s41598-018-24676-6 29674640 PMC5908836

[342] Fang W Cui H Yu D Chen Y Wang J Yu G. Increased expression of phospho-acetyl-CoA carboxylase protein is an independent prognostic factor for human gastric cancer without lymph node metastasis. Med Oncol (2014) 31(7):15. doi: 10.1007/s12032-014-0015-7 24924473

[343] Jia Y Ma Z Liu X Zhou W He S Xu X. Metformin prevents DMH-induced colorectal cancer in diabetic rats by reversing the warburg effect. Cancer Med (2015) 4(11):1730–41. doi: 10.1002/cam4.521 PMC4674000 26376762

[344] Park JK Coffey NJ Limoges A Le A. The heterogeneity of lipid metabolism in cancer. Adv Exp Med Biol (2021) 1311:39–56. doi: 10.1007/978-3-030-65768-0_3 34014533 PMC9703268

[345] Sardesai SD Thomas A Gallagher C Lynce F Ottaviano YL Ballinger TJ. Inhibiting fatty acid synthase in operable triple negative breast cancer. J Clin Oncol (2020) 38(15_suppl):584. doi: 10.1200/JCO.2020.38.15_suppl.584 31821065

[346] Wu X Dong Z Wang CJ Barlow LJ Fako V Serrano MA. FASN regulates cellular response to genotoxic treatments by increasing PARP-1 expression and DNA repair activity *via* NF-κB and SP1. Proc Natl Acad Sci U.S.A. (2016) 113(45):E6965–e73. doi: 10.1073/pnas.1609934113 PMC5111708 27791122

[347] Karran P Attard N. Thiopurines in current medical practice: molecular mechanisms and contributions to therapy-related cancer. Nat Rev Cancer (2008) 8(1):24–36. doi: 10.1038/nrc2292 18097462

[348] Brandalise SR Pinheiro VR Aguiar SS Matsuda EI Otubo R Yunes JA. Benefits of the intermittent use of 6-mercaptopurine and methotrexate in maintenance treatment for low-risk acute lymphoblastic leukemia in children: randomized trial from the Brazilian childhood cooperative group–protocol ALL-99. J Clin Oncol (2010) 28(11):1911–8. doi: 10.1200/jco.2009.25.6115 20212252

[349] Parker WB. Enzymology of purine and pyrimidine antimetabolites used in the treatment of cancer. Chem Rev (2009) 109(7):2880–93. doi: 10.1021/cr900028p PMC2827868 19476376

[350] Vora A Mitchell CD Lennard L Eden TO Kinsey SE Lilleyman J. Toxicity and efficacy of 6-thioguanine versus 6-mercaptopurine in childhood lymphoblastic leukaemia: a randomised trial. Lancet (2006) 368(9544):1339–48. doi: 10.1016/s0140-6736(06)69558-5 17046466

[351] Bonate PL Arthaud L Cantrell WR Jr. Stephenson K Secrist JA 3rd Weitman S. Discovery and development of clofarabine: a nucleoside analogue for treating cancer. Nat Rev Drug Discovery (2006) 5(10):855–63. doi: 10.1038/nrd2055 17016426

[352] Faderl S Wetzler M Rizzieri D Schiller G Jagasia M Stuart R. Clofarabine plus cytarabine compared with cytarabine alone in older patients with relapsed or refractory acute myelogenous leukemia: results from the CLASSIC I trial. J Clin Oncol (2012) 30(20):2492–9. doi: 10.1200/jco.2011.37.9743 PMC4874149 22585697

[353] Juliusson G Christiansen I Hansen MM Johnson S Kimby E Elmhorn-Rosenborg A. Oral cladribine as primary therapy for patients with b-cell chronic lymphocytic leukemia. J Clin Oncol (1996) 14(7):2160–6. doi: 10.1200/jco.1996.14.7.2160 8683250

[354] Lindemalm S Liliemark J Juliusson G Larsson R Albertioni F. Cytotoxicity and pharmacokinetics of cladribine metabolite, 2-chloroadenine in patients with leukemia. Cancer Lett (2004) 210(2):171–7. doi: 10.1016/j.canlet.2004.03.007 15183532

[355] Gandhi V Keating MJ Bate G Kirkpatrick P. Nelarabine. Nat Rev Drug Discovery (2006) 5(1):17–8. doi: 10.1038/nrd1933 16485343

[356] Herling CD Coombes KR Benner A Bloehdorn J Barron LL Abrams ZB. Time-to-progression after front-line fludarabine, cyclophosphamide, and rituximab chemoimmunotherapy for chronic lymphocytic leukaemia: a retrospective, multicohort study. Lancet Oncol (2019) 20(11):1576–86. doi: 10.1016/s1470-2045(19)30503-0 PMC7147008 31582354

[357] Holowiecki J Grosicki S Giebel S Robak T Kyrcz-Krzemien S Kuliczkowski K. Cladribine, but not fludarabine, added to daunorubicin and cytarabine during induction prolongs survival of patients with acute myeloid leukemia: a multicenter, randomized phase III study. J Clin Oncol (2012) 30(20):2441–8. doi: 10.1200/jco.2011.37.1286 22508825

[358] Heidelberger C Chaudhuri NK Danneberg P Mooren D Griesbach L Duschinsky R. Fluorinated pyrimidines, a new class of tumour-inhibitory compounds. Nature (1957) 179(4561):663–6. doi: 10.1038/179663a0 13418758

[359] Rich TA Shepard RC Mosley ST. Four decades of continuing innovation with fluorouracil: current and future approaches to fluorouracil chemoradiation therapy. J Clin Oncol (2004) 22(11):2214–32. doi: 10.1200/jco.2004.08.009 15169811

[360] Vaitkevicius VK Brennan MJ Beckett VL Kelly JE Talley RW. Clinical evaluation of cancer chemotherapy with 5-fluorouracil. Cancer (1961) 14:131–52. doi: 10.1002/1097-0142(196101/02)14:1<131::aid-cncr2820140118>3.0.co;2-3 13779639

[361] Conroy T Hammel P Hebbar M Ben Abdelghani M Wei AC Raoul JL. FOLFIRINOX or gemcitabine as adjuvant therapy for pancreatic cancer. N Engl J Med (2018) 379(25):2395–406. doi: 10.1056/NEJMoa1809775 30575490

[362] Earl HM Hiller L Howard HC Dunn JA Young J Bowden SJ. Addition of gemcitabine to paclitaxel, epirubicin, and cyclophosphamide adjuvant chemotherapy for women with early-stage breast cancer (tAnGo): final 10-year follow-up of an open-label, randomised, phase 3 trial. Lancet Oncol (2017) 18(6):755–69. doi: 10.1016/s1470-2045(17)30319-4 28479233

[363] Mini E Nobili S Caciagli B Landini I Mazzei T. Cellular pharmacology of gemcitabine. Ann Oncol (2006) 17(Suppl 5):v7–12. doi: 10.1093/annonc/mdj941 16807468

[364] Elion GB. The purine path to chemotherapy. Science (1989) 244(4900):41–7. doi: 10.1126/science.2649979 2649979

[365] Heczey A Louis CU Savoldo B Dakhova O Durett A Grilley B. CAR T cells administered in combination with lymphodepletion and PD-1 inhibition to patients with neuroblastoma. Mol Ther (2017) 25(9):2214–24. doi: 10.1016/j.ymthe.2017.05.012 PMC5589058 28602436

[366] Wei AH Döhner H Pocock C Montesinos P Afanasyev B Dombret H. Oral azacitidine maintenance therapy for acute myeloid leukemia in first remission. N Engl J Med (2020) 383(26):2526–37. doi: 10.1056/NEJMoa2004444 33369355

[367] Takebe N Cheng X Wu S Bauer K Goloubeva OG Fenton RG. Phase I clinical trial of the inosine monophosphate dehydrogenase inhibitor mycophenolate mofetil (cellcept) in advanced multiple myeloma patients. Clin Cancer Res (2004) 10(24):8301–8. doi: 10.1158/1078-0432.Ccr-04-0747 15623606

[368] Tong X Smith J Bukreyeva N Koma T Manning JT Kalkeri R. Merimepodib, an IMPDH inhibitor, suppresses replication of zika virus and other emerging viral pathogens. Antiviral Res (2018) 149:34–40. doi: 10.1016/j.antiviral.2017.11.004 29126899

[369] Turka LA Dayton J Sinclair G Thompson CB Mitchell BS. Guanine ribonucleotide depletion inhibits T cell activation. Mech Action immunosuppressive Drug mizoribine. J Clin Invest (1991) 87(3):940–8. doi: 10.1172/jci115101 PMC329885 1999502

[370] Valvezan AJ Turner M Belaid A Lam HC Miller SK McNamara MC. mTORC1 couples nucleotide synthesis to nucleotide demand resulting in a targetable metabolic vulnerability. Cancer Cell (2017) 32(5):624–38.e5. doi: 10.1016/j.ccell.2017.09.013 29056426 PMC5687294

[371] Nawrocki ST Griffin P Kelly KR Carew JS. MLN4924: a novel first-in-class inhibitor of NEDD8-activating enzyme for cancer therapy. Expert Opin Investig Drugs (2012) 21(10):1563–73. doi: 10.1517/13543784.2012.707192 22799561

[372] Soucy TA Smith PG Milhollen MA Berger AJ Gavin JM Adhikari S. An inhibitor of NEDD8-activating enzyme as a new approach to treat cancer. Nature (2009) 458(7239):732–6. doi: 10.1038/nature07884 19360080

[373] Baumann P Mandl-Weber S Völkl A Adam C Bumeder I Oduncu F. Dihydroorotate dehydrogenase inhibitor A771726 (leflunomide) induces apoptosis and diminishes proliferation of multiple myeloma cells. Mol Cancer Ther (2009) 8(2):366–75. doi: 10.1158/1535-7163.Mct-08-0664 19174558

[374] Madak JT Bankhead A 3rd Cuthbertson CR Showalter HD Neamati N. Revisiting the role of dihydroorotate dehydrogenase as a therapeutic target for cancer. Pharmacol Ther (2019) 195:111–31. doi: 10.1016/j.pharmthera.2018.10.012 30347213

[375] Kalyanaraman B Cheng G Hardy M Ouari O Lopez M Joseph J. A review of the basics of mitochondrial bioenergetics, metabolism, and related signaling pathways in cancer cells: Therapeutic targeting of tumor mitochondria with lipophilic cationic compounds. Redox Biol (2018) 14:316–27. doi: 10.1016/j.redox.2017.09.020 PMC5633086 29017115

[376] Tanaka K Sasayama T Irino Y Takata K Nagashima H Satoh N. Compensatory glutamine metabolism promotes glioblastoma resistance to mTOR inhibitor treatment. J Clin Invest (2015) 125(4):1591–602. doi: 10.1172/jci78239 PMC4396477 25798620

[377] Benjamin D Robay D Hindupur SK Pohlmann J Colombi M El-Shemerly MY. Dual inhibition of the lactate transporters MCT1 and MCT4 is synthetic lethal with metformin due to NAD+ depletion in cancer cells. Cell Rep (2018) 25(11):3047–58.e4. doi: 10.1016/j.celrep.2018.11.043 30540938 PMC6302548

[378] Witkiewicz AK Whitaker-Menezes D Dasgupta A Philp NJ Lin Z Gandara R. Using the "reverse warburg effect" to identify high-risk breast cancer patients: stromal MCT4 predicts poor clinical outcome in triple-negative breast cancers. Cell Cycle (2012) 11(6):1108–17. doi: 10.4161/cc.11.6.19530 PMC3335917 22313602

[379] Yang L Achreja A Yeung TL Mangala LS Jiang D Han C. Targeting stromal glutamine synthetase in tumors disrupts tumor microenvironment-regulated cancer cell growth. Cell Metab (2016) 24(5):685–700. doi: 10.1016/j.cmet.2016.10.011 27829138 PMC7329194

[380] Cantelmo AR Conradi LC Brajic A Goveia J Kalucka J Pircher A. Inhibition of the glycolytic activator PFKFB3 in endothelium induces tumor vessel normalization, impairs metastasis, and improves chemotherapy. Cancer Cell (2016) 30(6):968–85. doi: 10.1016/j.ccell.2016.10.006 PMC5675554 27866851

[381] De Bock K Georgiadou M Schoors S Kuchnio A Wong BW Cantelmo AR. Role of PFKFB3-driven glycolysis in vessel sprouting. Cell (2013) 154(3):651–63. doi: 10.1016/j.cell.2013.06.037 23911327

[382] Chan DA Sutphin PD Nguyen P Turcotte S Lai EW Banh A. Targeting GLUT1 and the warburg effect in renal cell carcinoma by chemical synthetic lethality. Sci Transl Med (2011) 3(94):94ra70. doi: 10.1126/scitranslmed.3002394 PMC3683134 21813754

[383] Liu Y Cao Y Zhang W Bergmeier S Qian Y Akbar H. A small-molecule inhibitor of glucose transporter 1 downregulates glycolysis, induces cell-cycle arrest, and inhibits cancer cell growth *in vitro* and in vivo. Mol Cancer Ther (2012) 11(8):1672–82. doi: 10.1158/1535-7163.MCT-12-0131 22689530

[384] Wood TE Dalili S Simpson CD Hurren R Mao X Saiz FS. A novel inhibitor of glucose uptake sensitizes cells to FAS-induced cell death. Mol Cancer Ther (2008) 7(11):3546–55. doi: 10.1158/1535-7163.MCT-08-0569 19001437

[385] Wu KH Ho CT Chen ZF Chen LC Whang-Peng J Lin TN. The apple polyphenol phloretin inhibits breast cancer cell migration and proliferation *via* inhibition of signals by type 2 glucose transporter. J Food Drug Anal (2018) 26(1):221–31. doi: 10.1016/j.jfda.2017.03.009 PMC9332637 29389559

[386] Andrianesis V Glykofridi S Doupis J. The renal effects of SGLT2 inhibitors and a mini-review of the literature. Ther Adv Endocrinol Metab (2016) 7(5-6):212–28. doi: 10.1177/2042018816676239 PMC5298360 28203358

[387] Shoshan MC. 3-bromopyruvate: targets and outcomes. J Bioenerg Biomembr (2012) 44(1):7–15. doi: 10.1007/s10863-012-9419-2 22298255

[388] Tomimoto A Endo H Sugiyama M Fujisawa T Hosono K Takahashi H. Metformin suppresses intestinal polyp growth in ApcMin/+ mice. Cancer Sci (2008) 99(11):2136–41. doi: 10.1111/j.1349-7006.2008.00933.x PMC11159964 18803638

[389] Zhang ZJ Zheng ZJ Kan H Song Y Cui W Zhao G. Reduced risk of colorectal cancer with metformin therapy in patients with type 2 diabetes: a meta-analysis. Diabetes Care (2011) 34(10):2323–8. doi: 10.2337/dc11-0512 PMC3177711 21949223

[390] Fontaine E. Metformin-induced mitochondrial complex I inhibition: Facts, uncertainties, and consequences. Front Endocrinol (Lausanne) (2018) 9:753. doi: 10.3389/fendo.2018.00753 30619086 PMC6304344

[391] Garon EB Christofk HR Hosmer W Britten CD Bahng A Crabtree MJ. Dichloroacetate should be considered with platinum-based chemotherapy in hypoxic tumors rather than as a single agent in advanced non-small cell lung cancer. J Cancer Res Clin Oncol (2014) 140(3):443–52. doi: 10.1007/s00432-014-1583-9 PMC3939783 24442098

[392] Bonnet S Archer SL Allalunis-Turner J Haromy A Beaulieu C Thompson R. A mitochondria-k+ channel axis is suppressed in cancer and its normalization promotes apoptosis and inhibits cancer growth. Cancer Cell (2007) 11(1):37–51. doi: 10.1016/j.ccr.2006.10.020 17222789

[393] Dunbar EM Coats BS Shroads AL Langaee T Lew A Forder JR. Phase 1 trial of dichloroacetate (DCA) in adults with recurrent malignant brain tumors. Invest New Drugs (2014) 32(3):452–64. doi: 10.1007/s10637-013-0047-4 PMC4455946 24297161

[394] Pardee TS Luther S Buyse M Powell BL Cortes J. Devimistat in combination with high dose cytarabine and mitoxantrone compared with high dose cytarabine and mitoxantrone in older patients with relapsed/refractory acute myeloid leukemia: ARMADA 2000 phase III study. Future Oncol (2019) 15(28):3197–208. doi: 10.2217/fon-2019-0201 31512500

[395] Quintela-Fandino M Apala JV Salgado AC Mouron SA Guerra JA Cortes MG. Abrogation of resistance against bevacizumab (Bev) by mitochondrial inhibition: A phase 0 randomized trial of bev plus ME344 or placebo in early HER2-negative breast cancer (HERNEBC). J Clin Oncol (2018) 36(15_suppl):2552. doi: 10.1200/JCO.2018.36.15_suppl.2552

[396] Hamid O Bauer TM Spira AI Olszanski AJ Patel SP Wasser JS. Epacadostat plus pembrolizumab in patients with SCCHN: Preliminary phase I/II results from ECHO-202/KEYNOTE-037. J Clin Oncol (2017) 35(15_suppl):6010. doi: 10.1200/JCO.2017.35.15_suppl.6010

[397] Scholtes MP de Jong FC Zuiverloon TCM Theodorescu D. Role of bladder cancer metabolic reprogramming in the effectiveness of immunotherapy. Cancers (Basel) (2021) 13(2):288. doi: 10.3390/cancers13020288 33466735 PMC7830378

[398] Tabernero J Luke JJ Joshua AM Varga AI Moreno V Desai J. BMS-986205, an indoleamine 2,3-dioxygenase 1 inhibitor (IDO1i), in combination with nivolumab (NIVO): Updated safety across all tumor cohorts and efficacy in pts with advanced bladder cancer (advBC). J Clin Oncol (2018) 36(15_suppl):4512. doi: 10.1200/JCO.2018.36.15_suppl.4512

[399] Clem BF O'Neal J Tapolsky G Clem AL Imbert-Fernandez Y Kerr DA. Targeting 6-Phosphofructo-2-Kinase ( PFKFB3) as a therapeutic strategy against cancer. Mol Cancer Ther (2013) 12(8):1461–70. doi: 10.1158/1535-7163.Mct-13-0097 PMC3742633 23674815

[400] Mondal S Roy D Sarkar Bhattacharya S Jin L Jung D Zhang S. Therapeutic targeting of PFKFB3 with a novel glycolytic inhibitor PFK158 promotes lipophagy and chemosensitivity in gynecologic cancers. Int J Cancer (2019) 144(1):178–89. doi: 10.1002/ijc.31868 PMC6261695 30226266

[401] Chong DL Ma LY Liu F Zhang ZR Zhao SR Huo Q. Synergistic antitumor effect of 3-bromopyruvate and 5-fluorouracil against human colorectal cancer through cell cycle arrest and induction of apoptosis. Anticancer Drugs (2017) 28(8):831–40. doi: 10.1097/Cad.0000000000000517 28816773

[402] Azevedo-Silva J Queirós O Baltazar F Ułaszewski S Goffeau A Ko YH. The anticancer agent 3-bromopyruvate: a simple but powerful molecule taken from the lab to the bedside. J Bioenerg Biomembr (2016) 48(4):349–62. doi: 10.1007/s10863-016-9670-z 27457582

[403] Kwiatkowska E Wojtala M Gajewska A Soszynski M Bartosz G Sadowska-Bartosz I. Effect of 3-bromopyruvate acid on the redox equilibrium in non-invasive MCF-7 and invasive MDA-MB-231 breast cancer cells. J Bioenerg Biomembr (2016) 48(1):23–32. doi: 10.1007/s10863-015-9637-5 26715289

[404] Billiard J Dennison JB Briand J Annan RS Chai D Colon M. Quinoline 3-sulfonamides inhibit lactate dehydrogenase a and reverse aerobic glycolysis in cancer cells. Cancer Metab (2013) 1(1):19. doi: 10.1186/2049-3002-1-19 24280423 PMC4178217

[405] Kim E-Y Chung T-W Han CW Park SY Park KH Jang SB. A novel lactate dehydrogenase inhibitor, 1-(Phenylseleno)-4-(Trifluoromethyl) benzene, suppresses tumor growth through apoptotic cell death. Sci Rep (2019) 9(1):3969. doi: 10.1038/s41598-019-40617-3 30850682 PMC6408513

[406] Purkey HE Robarge K Chen J Chen Z Corson LB Ding CZ. Cell active hydroxylactam inhibitors of human lactate dehydrogenase with oral bioavailability in mice. ACS Med Chem Lett (2016) 7(10):896–901. doi: 10.1021/acsmedchemlett.6b00190 27774125 PMC5066143

[407] Zhou M Zhao Y Ding Y Liu H Liu Z Fodstad O. Warburg effect in chemosensitivity: targeting lactate dehydrogenase-a re-sensitizes taxol-resistant cancer cells to taxol. Mol Cancer (2010) 9(1):33. doi: 10.1186/1476-4598-9-33 20144215 PMC2829492

[408] Dhillon S. Ivosidenib: First global approval. Drugs (2018) 78(14):1509–16. doi: 10.1007/s40265-018-0978-3 PMC6315051 30209701

[409] Kim ES. Enasidenib: First global approval. Drugs (2017) 77(15):1705–11. doi: 10.1007/s40265-017-0813-2 28879540

[410] Arif T Krelin Y Nakdimon I Benharroch D Paul A Dadon-Klein D. VDAC1 is a molecular target in glioblastoma, with its depletion leading to reprogrammed metabolism and reversed oncogenic properties. Neuro Oncol (2017) 19(7):951–64. doi: 10.1093/neuonc/now297 PMC5570220 28339833

[411] Ravagnan L Marzo I Costantini P Susin SA Zamzami N Petit PX. Lonidamine triggers apoptosis *via* a direct, bcl-2-inhibited effect on the mitochondrial permeability transition pore. Oncogene (1999) 18(16):2537–46. doi: 10.1038/sj.onc.1202625 10353597

[412] Kochuparambil ST Al-Husein B Goc A Soliman S Somanath PR. Anticancer efficacy of simvastatin on prostate cancer cells and tumor xenografts is associated with inhibition of akt and reduced prostate-specific antigen expression. J Pharmacol Exp Ther (2011) 336(2):496–505. doi: 10.1124/jpet.110.174870 21059805

[413] Massari F Di Nunno V Cubelli M Santoni M Fiorentino M Montironi R. Immune checkpoint inhibitors for metastatic bladder cancer. Cancer Treat Rev (2018) 64:11–20. doi: 10.1016/j.ctrv.2017.12.007 29407369

[414] Zarei M Rahbar MR Morowvat MH Nezafat N Negahdaripour M Berenjian A. Arginine deiminase: Current understanding and applications. Recent Pat Biotechnol (2019) 13(2):124–36. doi: 10.2174/1872208313666181220121400 30569861

[415] Harding JJ Telli ML Munster PN Le MH Molineaux C Bennett MK. Safety and tolerability of increasing doses of CB-839, a first-in-class, orally administered small molecule inhibitor of glutaminase, in solid tumors. J Clin Oncol (2015) 33(15_suppl):2512. doi: 10.1200/jco.2015.33.15_suppl.2512

[416] Aguilera O Munoz-Sagastibelza M Torrejon B Borrero-Palacios A Del Puerto-Nevado L Martinez-Useros J. Vitamin c uncouples the warburg metabolic switch in KRAS mutant colon cancer. Oncotarget (2016) 7(30):47954–65. doi: 10.18632/oncotarget.10087 PMC5216991 27323830

[417] Yun J Mullarky E Lu C Bosch KN Kavalier A Rivera K. Vitamin c selectively kills KRAS and BRAF mutant colorectal cancer cells by targeting GAPDH. Science (2015) 350(6266):1391–6. doi: 10.1126/science.aaa5004 PMC4778961 26541605

[418] Pan T Gao L Wu G Shen G Xie S Wen H. Elevated expression of glutaminase confers glucose utilization *via* glutaminolysis in prostate cancer. Biochem Biophys Res Commun (2015) 456(1):452–8. doi: 10.1016/j.bbrc.2014.11.105 25482439

[419] Flavin R Peluso S Nguyen PL Loda M. Fatty acid synthase as a potential therapeutic target in cancer. Future Oncol (2010) 6(4):551–62. doi: 10.2217/Fon.10.11 PMC3197858 20373869

[420] Flavin R Zadra G Loda M. Metabolic alterations and targeted therapies in prostate cancer. J Pathol (2011) 223(2):283–94. doi: 10.1002/path.2809 PMC3197856 21125681

[421] Pemble C Johnson LC Kridel SJ Lowther WT. Crystal structure of the thioesterase domain of human fatty acid synthase inhibited by orlistat. Nat Struct Mol Biol (2007) 14(8):704–9. doi: 10.1038/nsmb1265 17618296

[422] Hatzivassiliou G Zhao FP Bauer DE Andreadis C Shaw AN Dhanak D. ATP citrate lyase inhibition can suppress tumor cell growth. Cancer Cell (2005) 8(4):311–21. doi: 10.1016/j.ccr.2005.09.008 16226706

[423] Xiang XQ Saha AK Wen R Ruderman NB Luo ZJ. AMP-activated protein kinase activators can inhibit the growth of prostate cancer cells by multiple mechanisms. Biochem Biophys Res Commun (2004) 321(1):161–7. doi: 10.1016/j.bbrc.2004.06.133 15358229

[424] Xiao B Heath R Saiu P Leiper FC Leone P Jing C. Structural basis for AMP binding to mammalian AMP-activated protein kinase. Nature (2007) 449(7161):496–500. doi: 10.1038/nature06161 17851531

[425] Zadra G Photopoulos C Loda M. The fat side of prostate cancer. Biochim Biophys Acta (2013) 1831(10):1518–32. doi: 10.1016/j.bbalip.2013.03.010 PMC3766375 23562839

[426] Rozencweig M von Hoff DD Slavik M Muggia FM. Cis-diamminedichloroplatinum (II). a new anticancer drug. Ann Intern Med (1977) 86(6):803–12. doi: 10.7326/0003-4819-86-6-803 326117

[427] Zhou C Chen G Huang Y Zhou J Lin L Feng J. Camrelizumab plus carboplatin and pemetrexed versus chemotherapy alone in chemotherapy-naive patients with advanced non-squamous non-small-cell lung cancer (CameL): a randomised, open-label, multicentre, phase 3 trial. Lancet Respir Med (2021) 9(3):305–14. doi: 10.1016/s2213-2600(20)30365-9 33347829

[428] MacKenzie ED Selak MA Tennant DA Payne LJ Crosby S Frederiksen CM. Cell-permeating alpha-ketoglutarate derivatives alleviate pseudohypoxia in succinate dehydrogenase-deficient cells. Mol Cell Biol (2007) 27(9):3282–9. doi: 10.1128/mcb.01927-06 PMC1899954 17325041

[429] Pietrak B Zhao H Qi H Quinn C Gao E Boyer JG. A tale of two subunits: how the neomorphic R132H IDH1 mutation enhances production of αHG. Biochemistry (2011) 50(21):4804–12. doi: 10.1021/bi200499m 21524095

[430] Sciacovelli M Frezza C. Oncometabolites: Unconventional triggers of oncogenic signalling cascades. Free Radic Biol Med (2016) 100:175–81. doi: 10.1016/j.freeradbiomed.2016.04.025 PMC5145802 27117029

[431] Fazolini NP Cruz AL Werneck MB Viola JP Maya-Monteiro CM Bozza PT. Leptin activation of mTOR pathway in intestinal epithelial cell triggers lipid droplet formation, cytokine production and increased cell proliferation. Cell Cycle (2015) 14(16):2667–76. doi: 10.1080/15384101.2015.1041684 PMC4614828 26017929

[432] Jardé T Perrier S Vasson MP Caldefie-Chézet F. Molecular mechanisms of leptin and adiponectin in breast cancer. Eur J Cancer (2011) 47(1):33–43. doi: 10.1016/j.ejca.2010.09.005 20889333

[433] Zhu A Lee D Shim H. Metabolic positron emission tomography imaging in cancer detection and therapy response. Semin Oncol (2011) 38(1):55–69. doi: 10.1053/j.seminoncol.2010.11.012 21362516 PMC3075495

[434] Zhou Y Zhou Y Shingu T Feng L Chen Z Ogasawara M. Metabolic alterations in highly tumorigenic glioblastoma cells: preference for hypoxia and high dependency on glycolysis. J Biol Chem (2011) 286(37):32843–53. doi: 10.1074/jbc.M111.260935 PMC3173179 21795717

[435] Soth MJ Le K Di Francesco ME Hamilton MM Liu G Burke JP. Discovery of IPN60090, a clinical stage selective glutaminase-1 (GLS-1) inhibitor with excellent pharmacokinetic and physicochemical properties. J Med Chem (2020) 63(21):12957–77. doi: 10.1021/acs.jmedchem.0c01398 PMC9007139 33118821

[436] Lemberg KM Gori SS Tsukamoto T Rais R Slusher BS. Clinical development of metabolic inhibitors for oncology. J Clin Invest (2022) 132(1):16. doi: 10.1172/jci148550 PMC8718137 34981784

